# Efficacy of Hongjing I granule, an herbal medicine, in patients with mild to moderate erectile dysfunction in a randomized controlled trial

**DOI:** 10.3389/fphar.2024.1367812

**Published:** 2024-12-24

**Authors:** Run-Nan Xu, Jun Guo, Chun-He Zhang, Qing Zhou, Qiang Gen, Fu Wang, Yu Zhao, Xin-Yun Luo, Yan-Feng Li, Yi-Jia Fu, Xin Zhang, Wen-Zhi Wang, Jian-Xiong Ma, Jian Wang, Xiao-Jun Huang, Wen-Jie Huang, Bo-Dong Lv

**Affiliations:** ^1^ Department of Urology, School of Medicine, The Second Affiliated Hospital, Zhejiang University, Hangzhou, Zhejiang, China; ^2^ Key Laboratory of Integrative Chinese and Western Medicine for Prevention and Treatment of Sexual Dysfunction of Zhejiang Province, Hangzhou, China; ^3^ Department of Andrology, Xiyuan Hospital, China Academy of Chinese Medical Sciences, Beijing, China; ^4^ Department of Urology and Andrology, Yunnan Provincial Hospital of Traditional Chinese Medicine, Kunming, China; ^5^ Department of Andrology, The First Affiliated Hospital of Hunan University of Traditional Chinese Medicine, Changsha, China; ^6^ National Clinical Research Center for Chinese Medicine Acupuncture and Moxibustion, Tianjin, China; ^7^ Department of Andrology, The First Affiliated Hospital of Tianjin University of Traditional Chinese Medicine, Tianjin, China; ^8^ Department of Urology and Andrology, The Second Affiliated Hospital of Zhejiang Chinese Medical University, Hangzhou, China

**Keywords:** Chinese herbal formula, randomized control trial, Hongjing I granule, erectile dysfunction, traditional Chinese medicine (TCM)

## Abstract

**Background:**

HJIG is a potential treatment for erectile dysfunction (ED) that has been used in China for over 20 years. We conducted a multi-center, double-blind, randomized, placebo-controlled trial to evaluate the effectiveness and safety of the Chinese Herbal Medicine, Hongjing I granule (HJIG), in patients with mild to moderate erectile dysfunction (ED).

**Methods:**

This study is structured as a randomized, double-blind, placebo-controlled trial, executed across multiple centers. The recruitment strategy is primarily oriented towards patients demonstrating a pronounced preference for solely leveraging traditional Chinese medicine (TCM) interventions, a preference that is widely observed within TCM healthcare settings. A total of 100 patients, presenting with mild to moderate ED, specifically linked to the traditional diagnostic criteria of qi deficiency and blood stasis, will be enrolled. These participants will be randomly distributed between the HJIG (N = 50) and placebo (N = 50) arms. The designated treatment period is set at 8 weeks. Primary outcome measures encompass the International Index of Erectile Function-Erectile Function domain (IIEF-EF) score, the Sexual Encounter Profile (SEP), and scores derived from the traditional Chinese medicine symptom evaluation.

**Results:**

Of the 122 men enrolled, the baseline IIEF-EF score averaged 16.00 [IQR: 13.00, 18.00]. Eight weeks post-randomization, the HJIG group demonstrated a mean change in IIEF-EF scores of 7.80 (±3.25), compared to 3.33 (±3.90) in the placebo group, signifying a marked difference (*P* < 0.001). The median alterations in SEP3 scores were 0.50 [IQR: 0.36, 0.75] for the HJIG group and 0.50 [0.20, 0.67] for the placebo group, revealing a statistically relevant distinction (*P* = 0.05). In both primary outcomes, HJIG proved superior to the placebo. Additionally, improvements in TCM symptom scores were notably greater in the HJIG group relative to the placebo, with no adverse events reported across both groups.

**Conclusion:**

The Hongjing I granule significantly improved symptoms in patients with mild to moderate ED. However, to validate these findings, further extended randomized trials are warranted.

**Clinical Trial Registration:**

The study has been registered in the Chinese Clinical Trial Registry (ChiCTR) and the registration number was ChiCTR2000041127.

## Graphical Abstract

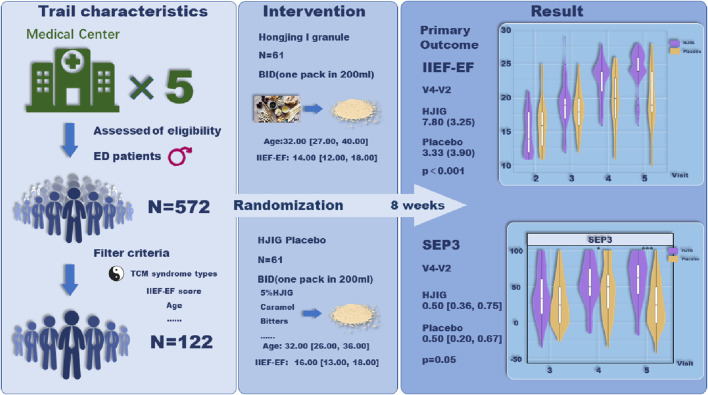

## Introduction

Erectile Dysfunction (ED) stands as a predominant sexual dysfunction in men, manifesting as a consistent inability to achieve or maintain an erection conducive to satisfactory sexual intercourse (Najari and Kashanian, 2016). Current estimates indicate that over 150 million men globally grapple with varying intensities of ED (Travison et al., 2007). Utilizing assessment tools such as IIEF, IIEF-5, and their related variants, there’s a discernible year-on-year escalation in ED incidence, mirroring the global aging demographic trend. Projections suggest that by 2025, the worldwide ED-afflicted populace might approach 322 million (Costa and Potempa, 2012).

The mainstay therapeutic intervention remains the Type 5 Phosphodiesterase inhibitors (PDE5i), yet its efficacy hovers between 60% and 70% (Ji et al., 2023). This has driven a substantial fraction of patients towards alternative medicine, predominantly herbal therapies (Muncey et al., 2021). Herbal formulations have a long-standing history and are widely used across Asia, particularly in China, Korea, and Japan, where they form an integral part of traditional medicinal practices (Bailly, 2024). The efficacy of herbal remedies or herbal formulations in treating erectile dysfunction has been corroborated by numerous studies (Qi et al., 2004; Kamenov et al., 2017; Borrelli et al., 2018; Choi et al., 2019; Chung et al., 2023). A burgeoning corpus of research intimates that Traditional Chinese Medicine (TCM) offers tangible benefits for ED (Wang et al., 2020; Wu et al., 2023), though some studies proffer equivocal conclusions (Xiong et al., 2014). Hence, there’s a pressing advocacy for rigorously orchestrated Randomized Controlled Trials (RCTs) to delve deeper into the subject.

Hongjing I granule (HJIG) is a traditional Chinese medicine prescribed by experienced physicians from the Second Affiliated Hospital of Zhejiang University School of Medicine and Dongzhimen Hospital of Beijing University of Chinese Medicine, with nearly 20 years of clinical application.

The traditional Chinese medicine theoretical basis for its application is elucidated as follows: The theory of Qi and Blood originates from the “Huangdi Neijing,” where the “Su Wen·Tiao Jing Lun” states, “Diseases arise and transform when the Qi and Blood are in disharmony.” Based on this, the harmony of Qi and Blood is closely associated with the onset and progression of diseases. Zhang Zhongjing, in his creation of the Six Meridian Diagnosis and Treatment System, also reflected the academic notion that all diseases are ultimately governed by the regulation of Qi and Blood. The “Zheng Zhi Gai Yao” mentions, “The penis, being constituted by sinews, relies on the nourishment of Qi and the moistening of Blood to become strong and vigorous.” This indicates that the penis is “structured by sinews and functions through the utility of Qi and Blood.” (Zhang G. et al., 2017). In a clinical study, 80 patients with mild to moderate ED were randomized into two groups: one receiving Tadalafil (5 mg qd) and the other a combination of Tadalafil and HJIG (1 pack bid) for 3 months. Evaluations of penile length, circumference, rigidity, IIEF-5 scores, partner satisfaction, and CT cavernosography showed post-treatment improvements in both groups (*P* < 0.05), with the combination therapy outperforming Tadalafil alone. Specifically, in the Tadalafil group, 33 cases showed venous leakage improvement with 7 cases completely resolved; the combination group had 17 cases improved and 23 cases fully resolved, indicating a statistically significant difference (χ2 = 13.65, *P* < 0.05) (Ping et al., 2021).

Additionally, 76 patients with Qi deficiency and blood stasis type mild to moderate ED received either Tadalafil (5 mg nightly for 12 weeks) or a combined Tadalafil and HJIG treatment (45 cases) with a step-down Tadalafil regimen. Both treatments significantly enhanced IIEF-5 and EHS scores (*P* < 0.05). The combined treatment group showed more substantial improvements in IIEF-5 and EHS scores after 4 weeks of medication and 4 weeks post-discontinuation compared to the Tadalafil only group (*P* < 0.01) (Zhang G. et al., 2017). Animal models have corroborated HJIG’s potential to promote nerve regeneration, attenuate hypoxia, curtail phenotype transformation, and stymie tissue fibrosis (Yang et al., 2014; Ye et al., 2019; Ma et al., 2020).

However, the evidence supporting the efficacy of HJIG in treating ED remains limited, and many studies on Chinese herbal formulations suffer from a lack of correlation assessment with their traditional use. It is essential to conduct high-quality clinical research to evaluate the therapeutic effectiveness of HJIG for ED and the validity of its application within traditional Chinese medicine frameworks.

HJIG is comprised of nine traditional Chinese plants, including Rhodiola crenulata (Hook. f. et Thoms.) H. Ohba [Crassulaceae; Rhodiola crenulata (Hook. f. et Thomas.) H. Ohba] (红景天), Astragalus mongholicus Bunge [Fabaceae; astragali radix praeparata cum melle] (黄芪), Codonopsis pilosula (Franch.) Nannf. [Campanulaceae; Codonopsis pilosula (Franch.) Nannf.] (党参), Salvia miltiorrhiza Bunge [Lamiaceae; Salvia miltiorrhiza Bge.] (丹参), Angelica sinensis (Oliv.) Diels [Apiaceae; Angelica sinensis (Oliv.) Diels] (全当归), Paeonia lactiflora Pall. [Paeoniaceae; Paeonia lactiflora Pall.] (白芍), Cyathula officinalis K.C.Kuan [Amaranthaceae; Cyathula officinalis Kuan] (川牛膝), Lycium chinense Mill. [Solanaceae; Lycium chinense Mill.] (枸杞), and Epimedium brevicornu Maxim. [Berberidaceae; Epimedium brevicornu Maxim.] (淫羊藿). The pharmacopoeial names are all referenced from the Chinese Pharmacopoeia 2015. The botanical names have been verified with MPNS (http://mpns.kew.org) (Access date: January 4, 2024).

Therefore, we designed this multicenter, double-blind, placebo-controlled randomized study. In addition to strict inclusion and exclusion criteria, we have also incorporated Traditional Chinese Medicine (TCM) syndrome scoring to evaluate whether the traditional medical basis of using HJIG (TCM syndrome) aligns with improvements in patient erectile function.

## Materials and methods

### Study design

This study is a multicenter, double-blind, placebo-controlled trial conducted in China, enrolling 122 ED patients seeking traditional Chinese medicine treatment from five distinct hospitals. Participants will be equally allocated to the HJIG herbal treatment group and a placebo group. The trial strictly adheres to the SPIRIT guidelines (Calvert et al., 2018), the Helsinki Declaration (Goodyear et al., 2007), and Good Clinical Practice (Guideline, 2001). In reporting details of the trial, we followed the recommendations of CONSORT Extension for Chinese Herbal Medicine Formulas 2017 (Cheng et al., 2017). Ethical approval for this study was obtained from the Ethics Committee of the Second Affiliated Hospital of Zhejiang Chinese Medical University (Approval ID: 2019-KL-012-01; see Supplementary Data Sheet S2). The trial is registered with ChiCTR (registration number: ChiCTR2000041127) and is coordinated by the Second Affiliated Hospital of Zhejiang Chinese Medical University, in partnership with four renowned institutions: The First Affiliated Hospital of Hunan University of Chinese Medicine, the First Affiliated Hospital of Tianjin University of Traditional Chinese Medicine, Yunnan Provincial Hospital of Traditional Chinese Medicine, and Xiyuan Hospital of the China Academy of Chinese Medical Sciences.

All participants provided written informed consent before randomization.

The more detailed research process of this trail can be retrieved from PubMed (Xu et al., 2023) (Supplementary Data Sheet S1).

### Selection criteria

#### Inclusion criteria

The predefined inclusion criteria were as follows:

(1) Diagnosis of erectile dysfunction based on traditional Chinese medicine standards: Erectile dysfunction is defined as an adult male’s inability to achieve or maintain an erection suitable for intercourse, or the rapid loss of an erection after achieving one. This condition must persist for a duration exceeding 3 months, as referenced by the T/CACM 2015-BZ076 standard from the China Association of Chinese Medicine.
(2) Diagnosed with “Qi deficiency” and “Blood stasis” in accordance with traditional Chinese medicine criteria, as outlined in the “Clinical Terminology of Traditional Chinese Medicine” publication by the National Technical Supervision Bureau, National Standard GB/16751.2-1997.
(3) Age ranging between 22 and 65 years.
(4) Demonstrated mild to moderate erectile dysfunction with an IIEF-5 score of >7 but ≤21.
(5) Engaged in a consistent heterosexual relationship for a minimum of 3 months.
(6) Willingness to attempt sexual intercourse a minimum of four times within each 4-week span throughout the study.
(7) At the preliminary assessment, made at least four sexual intercourse attempts, and recorded an IIEF-EF score between 11 and 25 during the second visit (a score of 17–25 indicates mild dysfunction, 11-16 points to moderate dysfunction, and ≤10 signifies severe dysfunction).
(8) Voluntary participation in the study with provided informed consent.

#### Exclusion criteria

Exclusion criteria included:

(1) Diabetes that’s uncontrolled with fasting glucose >120% of the normal upper limit.
(2) Erectile dysfunction resulting from spinal/neural injuries or surgeries related to prostate cancer.
(3) Penile deformities or risk of priapism (e.g., from sickle cell anemia).
(4) Presence of penile implants.
(5) Severe psychological disorders and impulse control problems.
(6) Untreated significant endocrine disorders, such as hypogonadism.
(7) Receipt of testosterone therapy within the last 3 months.
(8) History of significant cardiovascular events/issues within the past 6 months.
(9) Levels of AST, ALT, or creatinine that are more than twice the normal upper limit.
(10) History of bleeding disorders or active ulcers.
(11) Extreme blood pressure measurements (<90/50 or >170/100 mmHg).
(12) History of alcohol or drug abuse in the past 6 months, exceeding >14 units/week.
(13) An inability to maintain required study records.
(14) Presence of severe comorbid diseases.

### Drugs

Investigational Product: “Hongjing I Granules”, manufactured by Zhejiang Jolly Pharmaceutical Co., Ltd. It has been prescribed for the treatment of male erectile dysfunction by the Department of Urology at the Second Affiliated Hospital of Zhejiang Chinese Medical University. The details of the herbal formula are elaborated in Supplementary Data Sheet S3. The preparation process of HJIG is as follows: (1) Extraction: Qualified medicinal materials are pre-processed, and the prepared herbs and slices are extracted according to the specified process to obtain an extract; (2) Concentration: The extract filtrate is vacuum-concentrated to the required specific gravity to produce a clarified paste; (3) Spray Drying: The clear paste is sieved and spray-dried, with the powder being promptly collected to yield an intermediate product; (4) Sieving and Mixing: The intermediate product is sieved and mixed for 30 min to ensure uniformity; (5) Granulation: The mixture is dried and granulated into pellets. Packaging is conducted using blank aluminum foil bags with quality control reports and batch numbers of each herbal granule provided in Supplementary Data Sheet S3.

Placebo: A corresponding placebo of the herbal granules, also produced by Zhejiang Jolly Pharmaceutical Co., Ltd. It has been designed to mimic the appearance, color, and aroma of the “Hongjing I Granules” as shown in Supplementary Data Sheet S3.

We hereby confirm that the collection and processing of plant materials for this study fully comply with the Nagoya Protocol, CITES, all associated treaties including phytosanitary regulations, as well as the laws and regulations of China and the requirements of the Chinese Pharmacopoeia.

### Randomization and intervention

Patients with erectile dysfunction seeking traditional Chinese medicine treatment at five centers were invited to participate in this study. Those who expressed interest underwent comprehensive medical and sexual history evaluations, physical examinations, and the required laboratory tests. Four weeks post-evaluation, qualifying patients were stratified and block-randomized. Initially, stratification was based on centers, each hosting 20 participants subdivided into a control and trial group. Subsequent block randomization took into account appointment timing; every four consecutive patients were grouped.

The specific steps included: (1) determining block size and potential permutations for both groups, (2) allocating sample numbers to each permutation, and (3) randomly organizing block assignment numbers through a computer-generated random number table. These steps were followed to ensure that each participant had an equal chance of being assigned to either the treatment group or the placebo group. Upon inclusion confirmation and assignment of participant numbers, medication numbers based on statistical unit-provided random digits were designated. Throughout the trial, participants consistently used medication with identical numbers. Any medication with a specific number, if not wholly utilized, was not given to other participants.

Granules (either active drug or placebo, depending on the group assignment) provided by Zhejiang Jolly Pharmaceutical Co., Ltd., were dissolved in 200 mL hot water and taken twice daily. Both the primary medication, HJIG, and its placebo were indistinguishable in appearance, smell, and taste. They were packaged in identical bags and boxes. Consequently, physicians, data collectors, and patients remained blind to group allocations throughout the study. Treatment compliance was monitored through patient self-reports, regular follow-ups, and medication return checks, with detailed records of medication usage, treatment responses, and any adverse events to ensure data integrity and accuracy.

### Outcomes

All participants underwent an 8-week treatment, followed by a 12-week follow-up. Five visits were scheduled: at Week −4 (run-in), Week 0 (baseline), Week 4 (mid-treatment), Week 8 (end of treatment), and Week 12 (end of follow-up).

The primary outcome was designed to evaluate the improvements induced by HJIG in two composite endpoints. These composite endpoints reflected changes between Visit 2 and Visit 4 in:

(1) The percentage change in successful erections leading to ejaculation throughout all sexual encounters (Sexual Encounter Profile question 3, SEP3).
(2) The variation in IIEF-EF (International Index of Erectile Function - Erectile Function) specific scores. Differences between the HJIG group and the placebo group were assessed separately, and the primary outcome was considered achieved if both endpoint measures in the HJIG group were superior to those in the placebo group.

Secondary outcomes included:

(1) Stratified subgroup analysis of IIEF-EF and SEP results at Visit 4 based on participants’ baseline characteristics, such as age (>32 years old, ≤32 years old) and duration of ED (<12 months, ≥12 months but <36 months, and ≥36 months).
(2) Percentage change in successful erections that led to vaginal penetration at Visits 3 and 4 compared to Visit 2 (SEP2).
(3) Percentage variation in SEP3 and IIEF-EF scores from Visit 3 to Visit 2.
(4) Shift in Traditional Chinese Medicine symptom scores between Visit 3 or Visit 4 and Visit 2.
(5) Alteration in scores reflecting sexual desire (frequency of sexual activities) and overall satisfaction with sexual life between Visit 3 or Visit 4 and Visit 2.
(6) The proportion of participants with normalized IIEF-EF scores at Visit 4 relative to Visit 2.

The safety of HJIG was gauged by noting adverse events (AEs) and analyzing clinical laboratory results.

### Sample size calculation

This trail represents the first randomized controlled trial (RCT) to explore the efficacy of the traditional Chinese medicine formula, Hongjing I granule (HJIG), as a sole therapeutic agent for erectile dysfunction (ED). In 2017, we conducted a pilot trial involving patients seeking traditional Chinese medicine remedies for mild to moderate ED at the urology outpatient clinic of Zhejiang Province Xinhua Hospital. Upon diagnosis of Qi deficiency and Blood stasis by the primary physician, a secondary specialist in Chinese medicine was consulted to corroborate the diagnosis. If confirmed, the patient’s case number was documented, and an assessment using the International Index of Erectile Function - Erectile Function (IIEF-EF) was executed. Patients who then consumed Hongjing I granule for over 8 weeks underwent a follow-up IIEF-EF scoring. Those achieving a Minimal Clinically Important Difference of 5 points (Rosen et al., 2011) were deemed responsive. The effectiveness rate, reported by three physicians, ranged between 60% and 70% for their initial visit patients. This is congruent with the efficacy rate deduced by our andrology experts based on combined treatment clinical research findings and empirical observations. Thus, we postulated a 65% efficacy rate for the HJIG monotherapy. The core hypothesis for this investigation is the superiority of the HJIG cohort over the placebo cohort. The metrics for assessing this superiority encompass: 1. Changes in the Sexual Encounter Profile Question 3 (SEP3) and 2. Variations in specific scores of IIEF-EF. All superiority assessments are statistical, devoid of a defined superiority benchmark. As per pertinent literature, the test level is pegged at 0.05, with a test assurance degree of 0.9. The calculation for superiority, applicable for a 1:1 allocation between the test and control cohorts, is outlined below:

$$n=\frac{{\left(Z_{\alpha }+Z_{\beta }\right)}^{2}\left[P_{C}\left(1-P_{C}\right)+P_{T}\left(1-P_{T}\right)\right]}{{\left(P_{T}-P_{C}\right)}^{2}}$$

In this equation, PC denotes the efficacy rate of the control cohort, and PT represents the efficacy rate of the test cohort. α signifies a Type I error, conventionally set at α = 0.05, yielding Z_α_ = 1.645; β stands for a Type II error, typically marked at β = 0.10, leading to Z_β_ = 1.282. Drawing from available research datasets, the clinical efficacy rate of a placebo in ED studies stands at PC = 0.33 (Feys et al., 2014). Based on our projected efficacy rate of HJIG at PT = 0.65, and considering α = 0.05 and β = 0.10, the relevant data was integrated into the superiority equation, producing an n ≈ 40. Factoring in a 20% attrition rate, an estimated 50 subjects per group are required, summing to a minimum of 100 subjects altogether. Given potential variations between the pilot trial and actual therapeutic outcomes, we petitioned the ethics committee for a potential enlargement of the sample size during the study duration (2021.1–2022.12). This was contingent on available funds to counteract dropouts and study discontinuations. We preemptively arranged for 200 trial drug allocations (100 for placebo and 100 for HJIG) to offset potential attrition. In the end, a total of 122 patients were enrolled.

### Statistical analysis

For continuous variables, if the data do not follow a normal distribution, they will be presented using the median [interquartile range]; if they are normally distributed, they will be expressed as the mean (standard deviation). Categorical variables will be presented as the number of cases (percentage). Before comparing differences in various indicators between the experimental and control groups, the Shapiro-Wilk test will be applied to check the normality of continuous variables. If the variables are normally distributed, a t-test will be applied; otherwise, the Wilcoxon test will be used. For categorical variable comparisons, the Chi-square test will be employed. All analyses will be conducted using R software (version 4.2.3), and a *P*-value <0.05 will be considered statistically significant (Zhang et al., 2023).

## Results

### Patient characteristics

**FIGURE 1:**
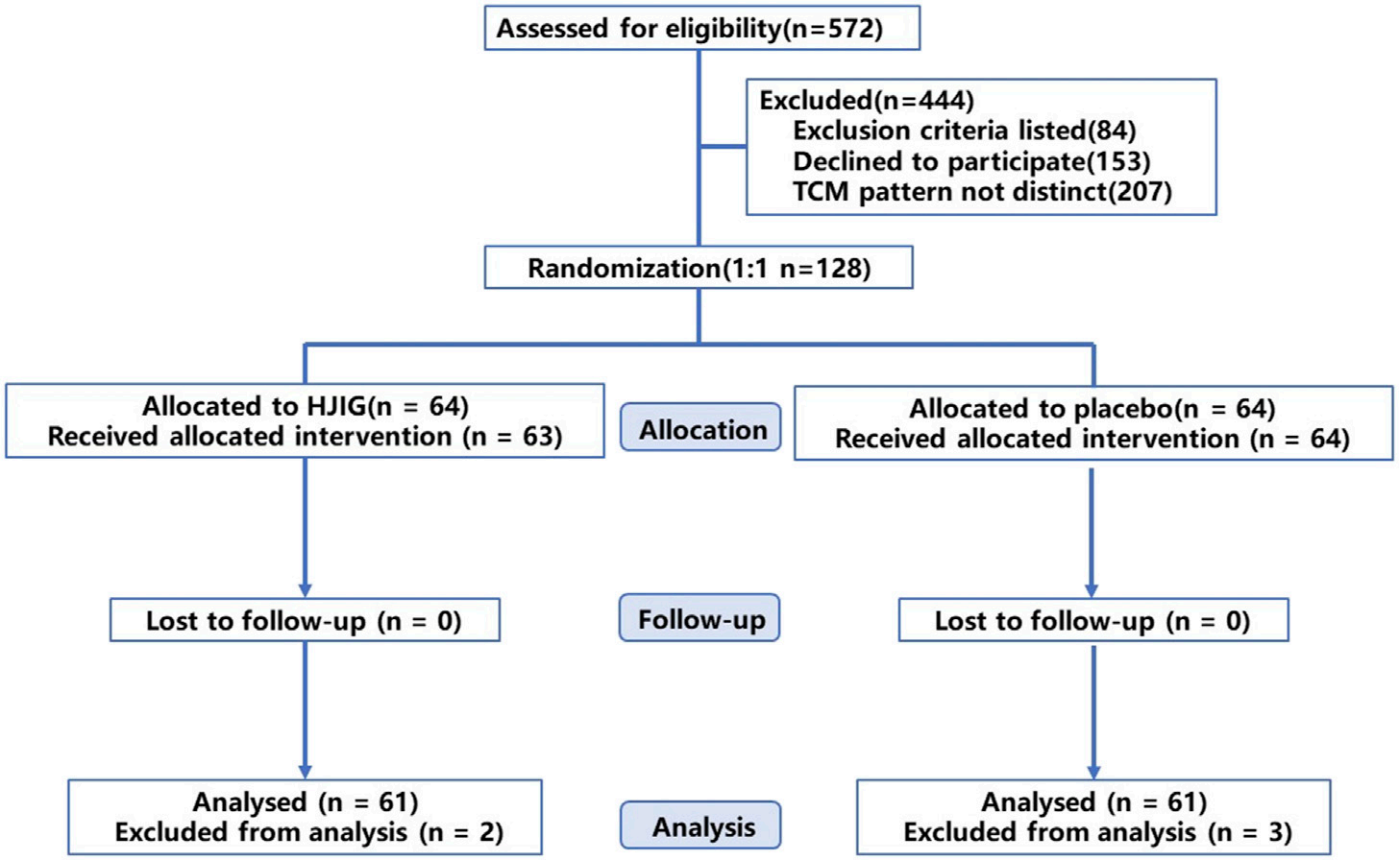
Patient flow chart.

Between January 2021 and December 2022, we screened a total of 572 potential participants. Of these, 128 were randomly assigned to either the HJIG or placebo groups, with 64 participants in each group. Six participants withdrew from the study: one due to dissatisfaction with the results, and five due to self-administration of PDE5i prior to engaging in sexual activity. Among those who completed the study, 100% (122/122) adhered to at least 80% of the prescribed dosage, as determined by calculating the returned medication. The participant flow is illustrated in Figure 1. Baseline characteristics were well-balanced between the two groups (*P* > 0.05), as detailed in Table 1.

**TABLE 1:** Baseline characteristics of participants.

	Total	HJIG	placebo	*P*
n	122	61	61	
Age	32.00 [26.00, 38.00]	32.00 [27.00, 40.00]	32.00 [26.00, 36.00]	0.159
Height	173.00 [170.00, 176.00]	173.00 [170.00, 175.00]	173.00 [170.00, 177.00]	0.994
Weight	72.00 [68.00, 77.50]	73.00 [68.00, 75.00]	71.00 [68.00, 78.00]	0.873
History of ED	12.00 [6.00, 33.00]	12.00 [6.00, 36.00]	12.00 [6.00, 24.00]	0.485
IIEF-EF-Total	16.00 [13.00, 18.00]	14.00 [12.00, 18.00]	16.00 [13.00, 18.00]	0.027
IIEF-EF-q1	3.00 [2.00, 3.00]	3.00 [2.00, 3.00]	3.00 [2.00, 3.00]	0.456
IIEF-EF-q2	3.00 [2.00, 3.00]	3.00 [2.00, 3.00]	3.00 [2.00, 3.00]	0.060
IIEF-EF-q3	3.00 [2.00, 3.00]	3.00 [2.00, 3.00]	3.00 [2.00, 3.00]	0.093
IIEF-EF-q4	3.00 [2.00, 3.00]	2.00 [2.00, 3.00]	3.00 [2.00, 3.00]	0.159
IIEF-EF-q5	3.00 [2.00, 3.00]	3.00 [2.00, 3.00]	3.00 [2.00, 3.00]	0.336
IIEF-EF-q6	2.00 [2.00, 3.00]	2.00 [2.00, 2.00]	2.00 [2.00, 3.00]	0.001
Primary Symptoms of Qi Deficiency	12.00 [8.00, 14.00]	12.00 [10.00, 16.00]	12.00 [8.00, 14.00]	0.057
Secondary Symptoms of Qi Deficiency	4.00 [2.25, 5.00]	4.00 [2.00, 5.00]	4.00 [3.00, 5.00]	0.600
Primary Symptoms of Blood Stasis	18.00 [12.00, 20.00]	18.00 [12.00, 20.00]	14.00 [12.00, 20.00]	0.848
Secondary Symptoms of Blood Stasis	4.00 [2.25, 5.00]	4.00 [3.00, 6.00]	4.00 [2.00, 5.00]	0.603
SEP1	1.00 [0.75, 1.00]	1.00 [0.75, 1.00]	1.00 [0.75, 1.00]	0.110
SEP2	0.55 [0.40, 0.75]	0.50 [0.29, 0.75]	0.75 [0.50, 1.00]	0.106
SEP3	0.25 [0.00, 0.50]	0.25 [0.00, 0.40]	0.25 [0.00, 0.50]	0.953
SEP4	0.00 [0.00, 0.16]	0.00 [0.00, 0.12]	0.00 [0.00, 0.25]	0.527
SEP5	0.00 [0.00, 0.25]	0.00 [0.00, 0.25]	0.00 [0.00, 0.25]	0.689
Severity Distribution (%)				0.268
Mild Moderate	49 (40.2%) 73 (59.8%)	21 (34.4%) 40 (65.6%)	28 (45.9%) 33 (54.1%)	
History of diabetes (%)	3 (2.5%)	0 (0%)	3 (5%)	
Hyperlipidemia (%)	2 (1.64%)	2 (3.2%)	0 (0%)	

Age: Patient’s age (years), median [interquartile range]; Height: Patient’s height (cm), median [interquartile range]; Weight: Patient’s weight (kg), median [interquartile range]; Duration of Erectile Dysfunction History: Length of erectile dysfunction history (years), median [interquartile range]; IIEF-EF-Total: International Index of Erectile Function - Erectile Function Domain total score, median [interquartile range], IIEF-EF-q1∼6: IIEF-EF, item 1∼6 score, median [interquartile range]; Primary Symptoms of Qi Deficiency: Main symptoms of Qi deficiency, median [interquartile range]; Secondary Symptoms of Qi Deficiency: Secondary symptoms of Qi deficiency, median [interquartile range]; Primary Symptoms of Blood Stasis: Main symptoms of blood stasis, median [interquartile range], Secondary Symptoms of Blood Stasis: Secondary symptoms of blood stasis, median [interquartile range]; SEP1∼5: Percentage of “yes” responses to SEP, question 1∼5 after sexual encounters, median [interquartile range]; Severity Distribution (%): Percentage of patients with mild/moderate ED, Number of patients (percentage).

### Primary outcome assessment

**FIGURE 2:**
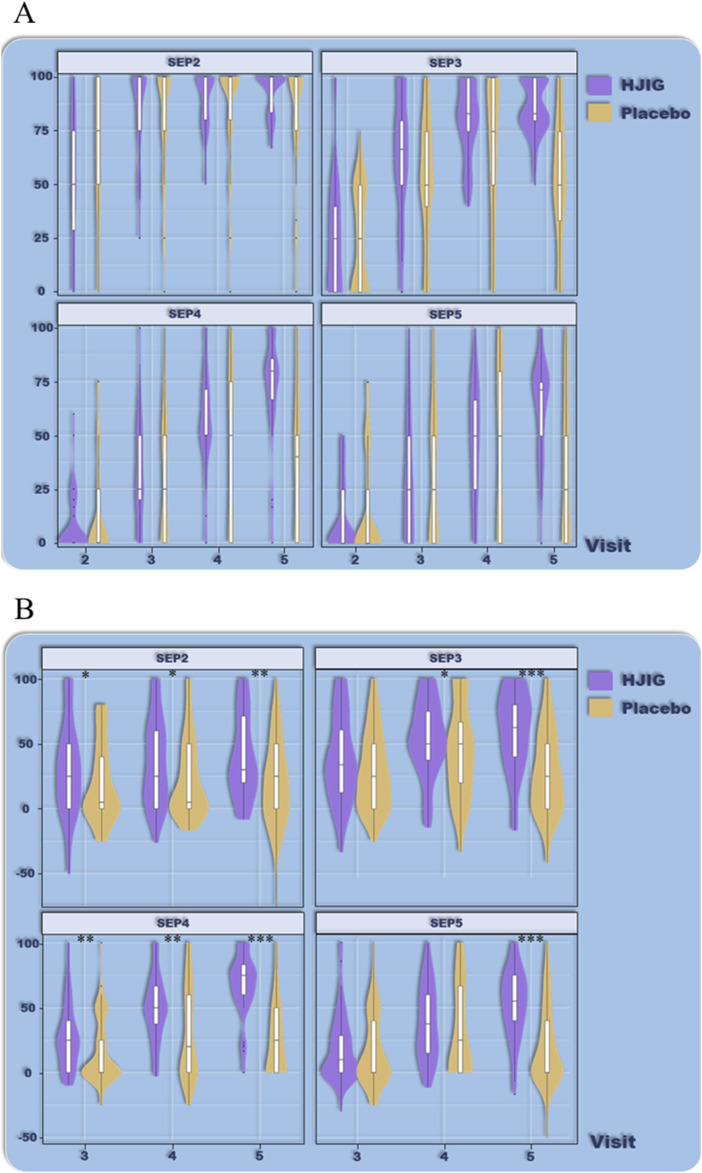
**(A)** Percentage of “Yes” responses to SEP questions 2–4 at each Visit point. **(B)** Difference in percentage of “Yes” responses to SEP questions 2–4 between visits 3–5 and visit 2.

**FIGURE 3:**
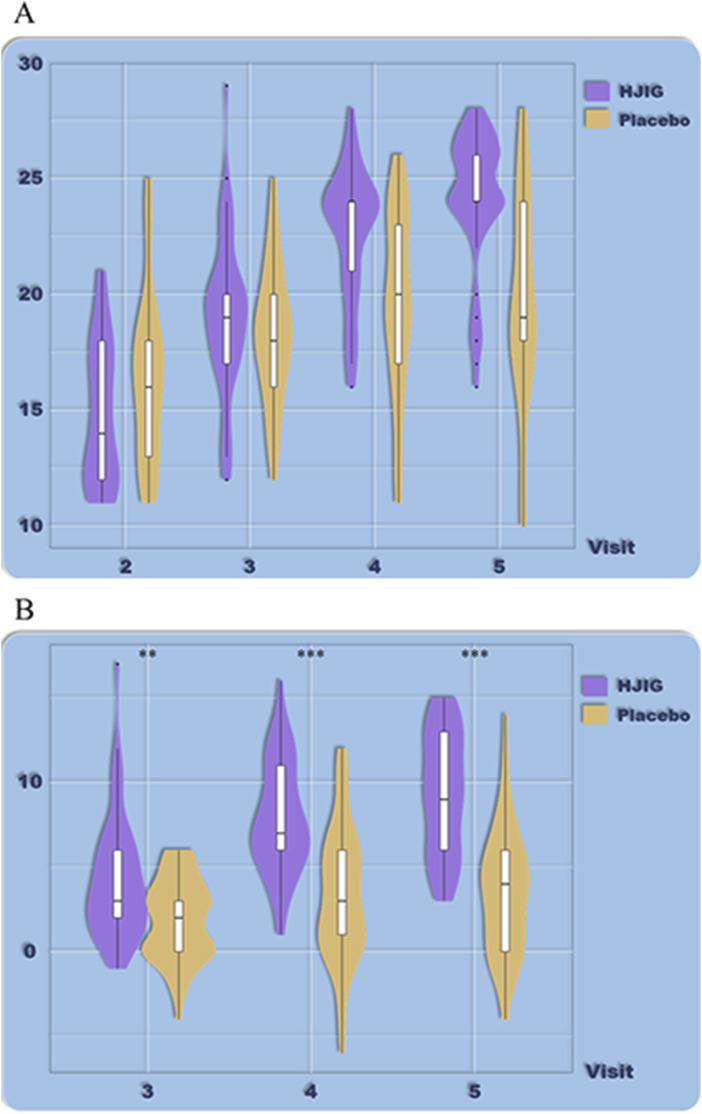
**(A)** Total IIEF-EF scores at each visit. **(B)** Differences in IIEF-EF scores between visits 3–5 and visit 2.

At Visit 4, the percentage change in SEP3 for both patient groups was evaluated relative to Visit 2 (Figure 2). For the HJIG group, the median change was 0.50 [IQR: 0.36, 0.75], whereas for the placebo group, it was 0.50 [0.20, 0.67]. The statistical difference between the two groups is significant (*P* = 0.05) (Karppinen et al., 2001; Romano, 2002), HJIG treatment led to an increase in the proportion of successful ejaculations during sexual encounters compared to the placebo group; further details are provided in Table 2. The IIEF-EF scores for both groups demonstrated an increasing trend with prolonged treatment. Notably, the HJIG group showed a more pronounced increase and higher scores than the placebo group subsequent to Visit 2 (Figure 3). Eight weeks post-randomization, the mean change in the IIEF-EF score for the HJIG group was 7.80 (±3.25) compared to 3.33 (±3.90) for the placebo group. The difference between the two groups was statistically significant (*P* < 0.001), HJIG treatment resulted in a significant improvement in erectile function, which was superior to the placebo group.

**TABLE 2:** Changes in scores when comparing visits 3, 4, and 5 to visit 2.

	HJIG	placebo	*P*
n	61	61	
IIEF-EF (v3-v2)	3.00 [2.00, 6.00]	2.00 [0.00, 3.00]	<0.001
Severity Distribution (%)			0.266
Normal	1 (1.6%)	0 (0.0%)	
Mild	50 (82.0%)	45 (73.8%)	
Moderate	10 (16.4%)	16 (26.2%)	
SEP1 (v3-v2)	0.00 [0.00, 0.25]	0.00 [0.00, 0.25]	0.132
SEP2 (v3-v2)	0.25 [0.00, 0.50]	0.00 [0.00, 0.50]	0.022
SEP3 (v3-v2)	0.35 [0.14, 0.75]	0.25 [0.00, 0.50]	0.062
SEP4 (v3-v2)	0.25 [0.00, 0.40]	0.00 [0.00, 0.25]	0.006
SEP5 (v3-v2)	0.10 [0.00, 0.33]	0.00 [0.00, 0.40]	0.694
IIEF-EF (v4-v2)	7.80 (±3.25)	3.33 (±3.90)	<0.001
Severity Distribution (%)			0.001
Normal	7 (11.5%)	1 (1.6%)	
Mild	52 (85.2%)	46 (75.4%)	
Moderate	2 (3.3%)	14 (23.0%)	
SEP1 (v4-v2)	0.00 [0.00, 0.25]	0.00 [0.00, 0.25]	0.128
SEP2 (v4-v2)	0.25 [0.08, 0.58]	0.05 [0.00, 0.50]	0.023
SEP3 (v4-v2)	0.50 [0.36, 0.75]	0.50 [0.20, 0.67]	0.050
SEP4 (v4-v2)	0.50 [0.35, 0.67]	0.20 [0.00, 0.62]	0.004
SEP5 (v4-v2)	0.29 [0.14, 0.60]	0.25 [0.00, 0.67]	0.232
IIEF-EF (v5-v2)	9.00 [6.00, 13.00]	4.00 [0.00, 6.00]	<0.001
Severity Distribution (%)			0.001
Normal	24 (39.3%)	8 (13.1%)	
Mild	36 (59.0%)	41 (67.2%)	
Moderate	1 (1.6%)	9 (14.8%)	
Severe	0 (0.0%)	3 (4.9%)	
SEP1 (v5-v2)	0.00 [0.00, 0.25]	0.00 [0.00, 0.25]	0.153
SEP2 (v5-v2)	0.30 [0.20, 0.71]	0.25 [0.00, 0.50]	0.003
SEP3 (v5-v2)	0.63 [0.40, 0.80]	0.25 [0.00, 0.50]	<0.001
SEP4 (v5-v2)	0.75 [0.60, 0.83]	0.25 [0.00, 0.50]	<0.001
SEP5 (v5-v2)	0.55 [0.40, 0.75]	0.00 [0.00, 0.40]	<0.001

IIEF-EF (v3∼5-v2): The difference in the total score of the 6 questions of the IIEF-EF, between each visit point (V3∼V5) and visit 2, median [interquartile range](Data not normally distributed), Mean (standard deviation) (Data normally distributed); SEP1∼5 (v3∼5-v2): The difference in the percentage of “yes” responses to each SEP, question after sexual encounters between each visit point (V3∼V5) and visit 2, median [interquartile range]; Severity Distribution (%): Percentage of patients with normal/mild/moderate/severe ED, Number of patients (percentage).

Refer to Table 2 for a detailed depiction.

### Secondary outcome assessment

#### IIEF-EF and SEP results in subgroup analysis

##### Age-stratified subgroup analysis (>32 years old, ≤32 years old)

**FIGURE 4:**
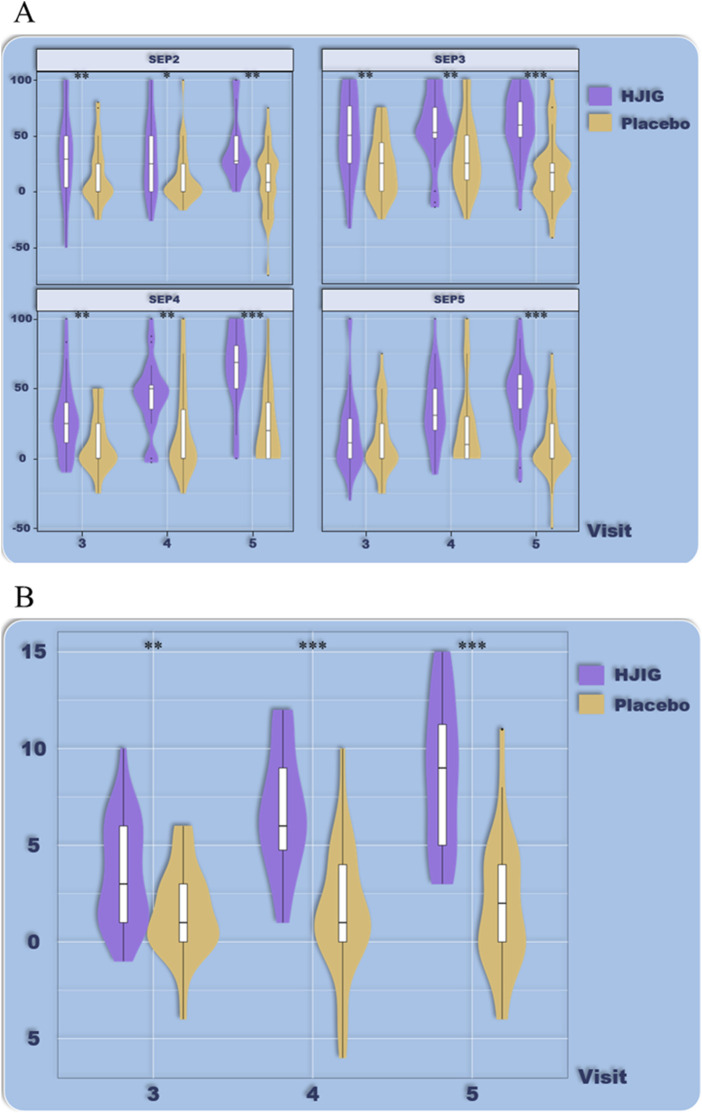
Age-stratified subgroup analysis (age >32), **(A)** Percentage of “Yes” responses to SEP questions 2–4 at each visit point, **(B)** Differences in IIEF-EF scores between visits 3–5 and visit 2.

**FIGURE 5:**
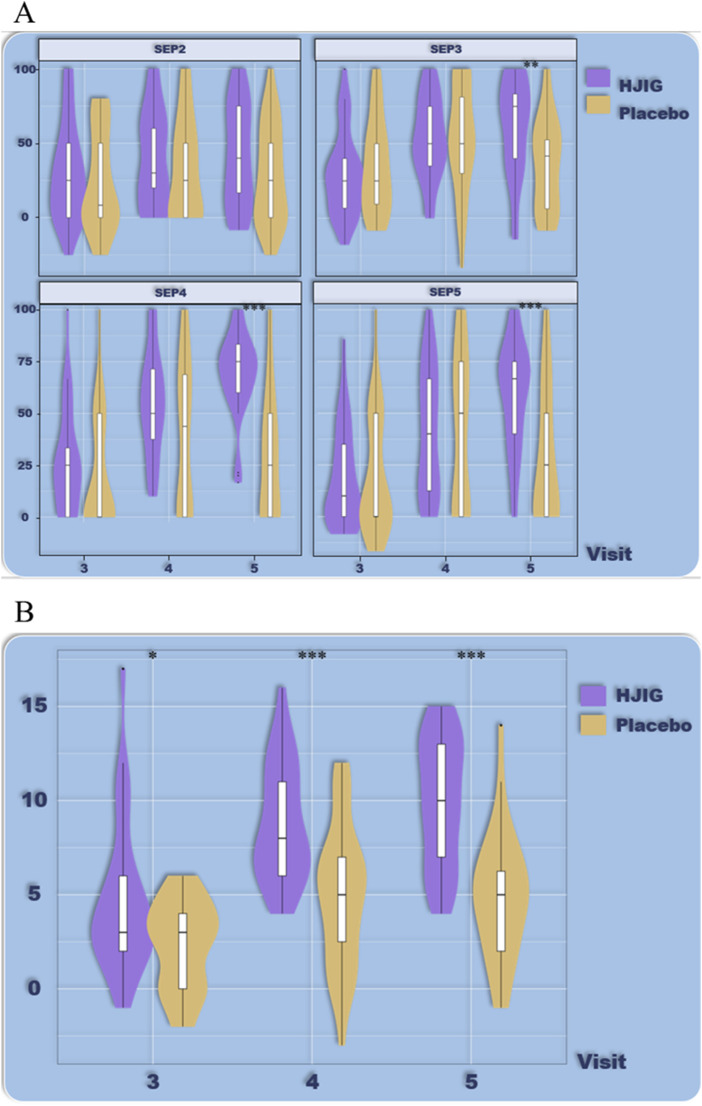
Age-stratified subgroup analysis (age ≤32), **(A)** Percentage of “Yes” responses to SEP questions 2–4 at each visit point, **(B)** Differences in IIEF-EF scores between visits 3–5 and visit 2.

Based on the IIEF-EF scores, there was no significant difference in efficacy between patients older than 32 (Figure 4) and those younger than 32 (Figure 5). The improvements in IIEF-EF scores for patients treated with HJIG from visits 3–5 were consistently higher than those in the placebo group, suggesting that HJIG treatment effectively enhances erectile function. Additionally, a higher proportion of patients achieved normalization in the HJIG group. The results of the SEP questions exhibited certain differences after stratifying by age. For patients older than 32 years, the percentage difference in SEP3 “yes” responses per total sexual encounters was significantly higher in the treatment group compared to the control group by visit 3, indicating that HJIG treatment increased the number of times patients were able to maintain an erection until the completion of intercourse. For patients 32 years or younger, the percentage difference in SEP3 responses only showed a significant difference between the HJIG group and the placebo group by visit 5. A similar trend was observed in the other SEP questions.

##### ED Duration-stratified subgroup analysis (≤12 months, >12 to <36 months, ≥36 months)

###### Analysis based on a 12-month ED history revealed

**FIGURE 6:**
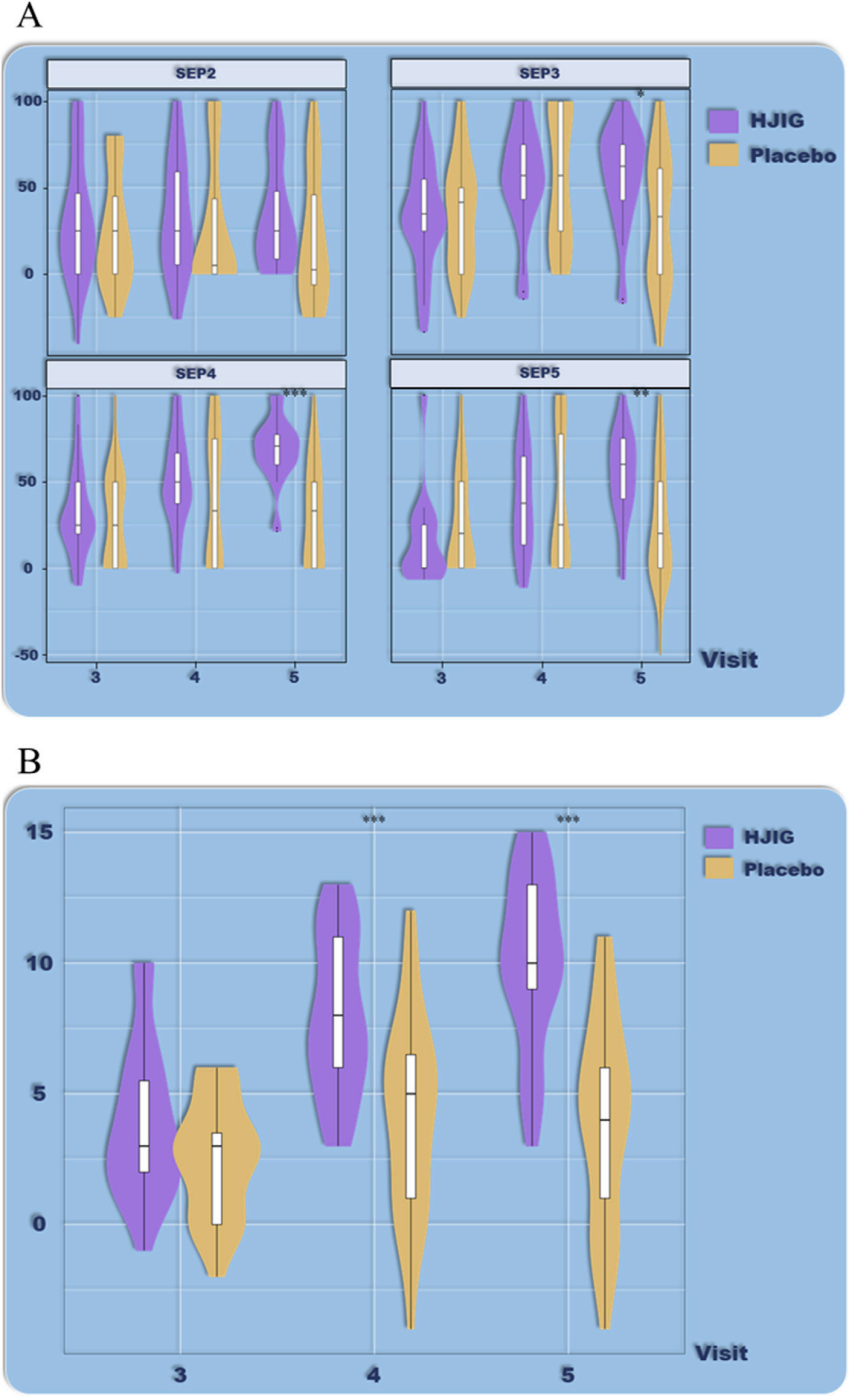
ED Duration-stratified subgroup analysis (≤12 months), **(A)** Percentage of “Yes” responses to SEP questions 2–4 at each visit point, **(B)** Differences in IIEF-EF scores between visits 3–5 and visit 2.

Using 12 months as the cut-off point for disease duration, the results indicate that for patients with a disease history longer than 12 months, HJIG significantly improved overall erectile function. At visit 4, the improvement in IIEF-EF scores in the HJIG group was significantly higher than that in the placebo group and was maintained until visit 5 (Figure 6). Regarding sexual completion as assessed by the SEP3 percentage difference, there was no significant difference between the two groups at visit 4, but a significant difference emerged at visit 5 (Figure 6).

**FIGURE 7:**
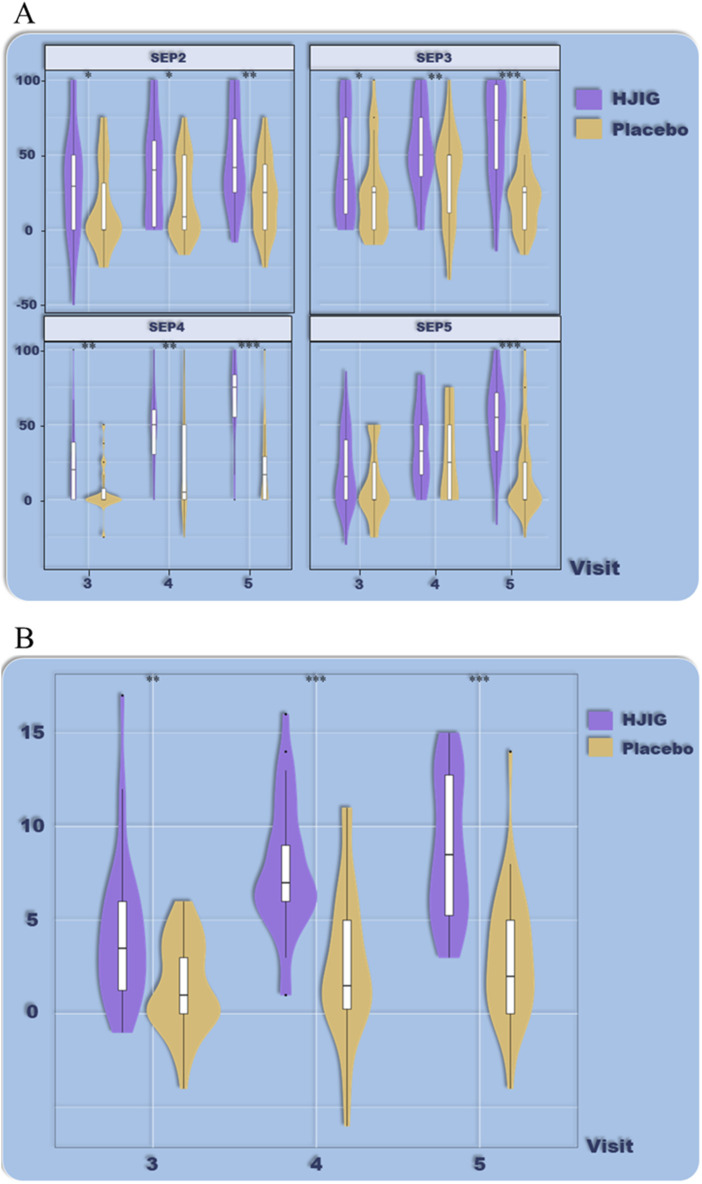
ED Duration-stratified subgroup analysis (>12 months), **(A)** Percentage of “Yes” responses to SEP questions 2–4 at each visit point, **(B)** Differences in IIEF-EF scores between visits 3–5 and visit 2.

For patients with a disease history of 12 months or less, HJIG also significantly improved overall erectile function. At visit 3, the improvement in IIEF-EF scores in the HJIG group was significantly higher than that in the placebo group and was maintained until visit 5 (Figure 7). In terms of sexual completion based on the SEP3 percentage difference, at visit 3, the improvement in the SEP3 percentage in the HJIG group was significantly higher than in the placebo group, and this improvement became more pronounced with the continuation of treatment and observation time (Figure 7). In summary, using 12 months as a threshold for stratifying disease history, HJIG was effective in improving overall erectile function and the ability to maintain an erection until the completion of sexual intercourse in both patients with a disease history longer than 12 months and those with a disease history of 12 months or less. However, for patients with a disease history of 12 months or less, the therapeutic effects manifested earlier than in those with a disease history longer than 12 months.

###### Considering a 36-month ED history boundary

**FIGURE 8:**
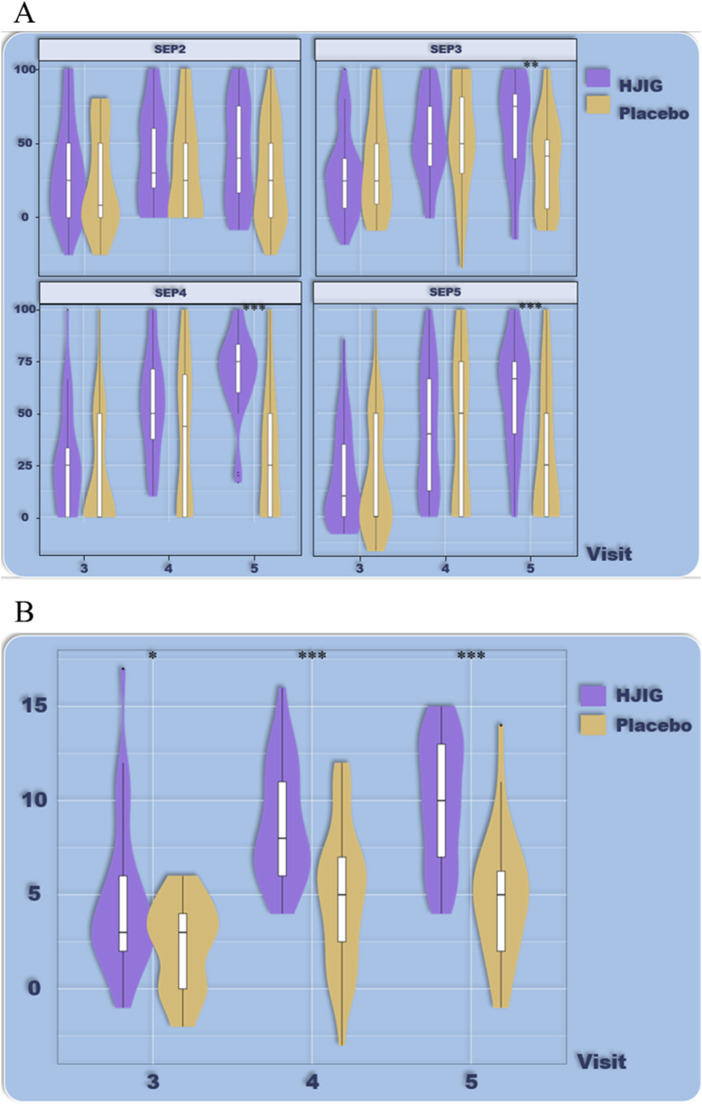
ED Duration-stratified subgroup analysis (≥36 months), **(A)** Percentage of “Yes” responses to SEP questions 2–4 at each Visit point, **(B)** Differences in IIEF-EF scores between visits 3–5 and visit 2.

Using 36 months as the cut-off point for disease duration, the results indicate that for patients with a disease history of 36 months or longer, HJIG significantly improved overall erectile function. At visit 4, the improvement in IIEF-EF scores in the HJIG group was significantly higher than that in the placebo group and was maintained until visit 5 (Figure 8). Regarding sexual completion as assessed by the SEP3 percentage difference, there was no significant difference between the two groups at visit 4, but significant differences emerged at visits 3 and 5 (Figure 8).

**FIGURE 9:**
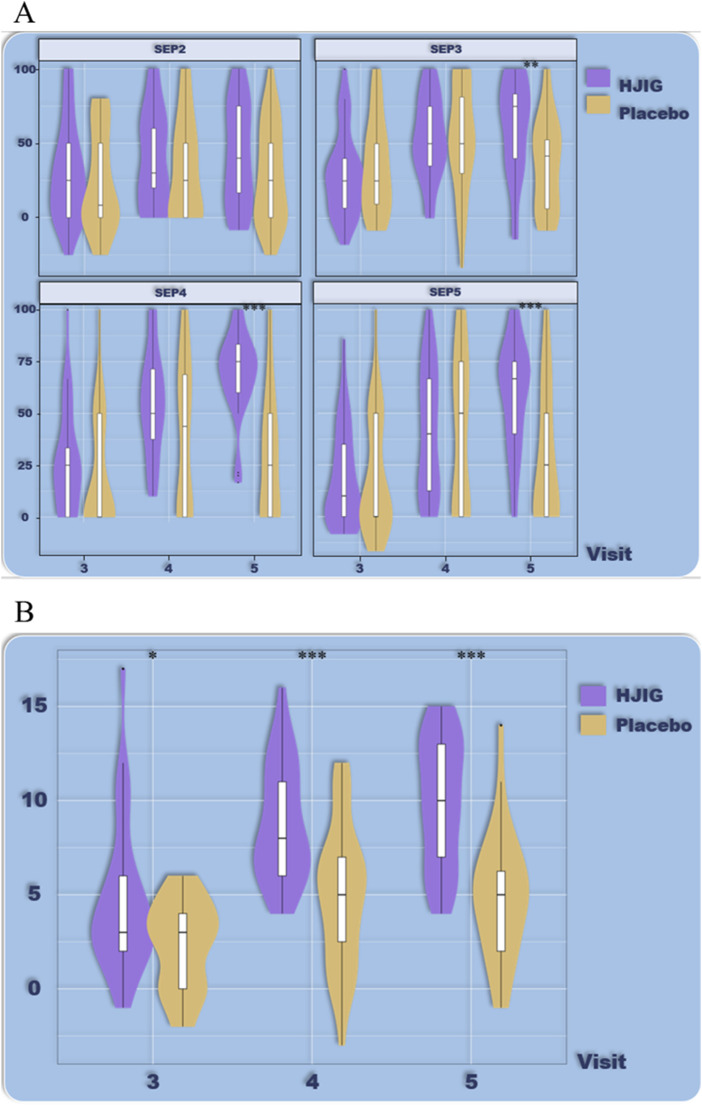
ED Duration-stratified subgroup analysis (<36 months), **(A)** Percentage of “Yes” responses to SEP questions 2–4 at each visit point, **(B)** Differences in IIEF-EF scores between visits 3–5 and visit 2.

For patients with a disease history of less than 36 months, HJIG also significantly improved overall erectile function. At visit 3, the improvement in IIEF-EF scores in the HJIG group was significantly higher than that in the placebo group and was maintained until visit 5 (Figure 9). In terms of sexual completion based on the SEP3 percentage difference, the improvement in the SEP3 percentage in the HJIG group was significantly higher than in the placebo group only at visit 5 (Figure 9).

In summary, using 36 months as a threshold for stratifying disease history, HJIG was effective in improving overall erectile function and the ability to maintain an erection until the completion of sexual intercourse in both patients with a disease history of 36 months or longer and those with a disease history of less than 36 months. However, patients with a disease history of 36 months or longer may require a longer treatment period and recovery time to achieve significant improvements in overall erectile function. For SEP3 percentage evaluation of sexual completion, both disease history stratification results showed a significant difference between the HJIG group and the placebo group at visit 5. However, for patients with an ED history of 36 months or longer, a significant difference between the HJIG group and the placebo group was observed as early as visit 3. At visit 4, this significance diminished and was near the margin of significance. Considering the high dispersion of the SEP percentage difference data and the relatively small number of patients with an ED history of 36 months or longer, as well as the substantial difference in the number of patients between the two groups, the interpretation of this result should be approached with caution.

#### Percentage of erections achievable for vaginal insertion (SEP2)

Comparing the percentage of successful erections suitable for vaginal insertion (SEP2) between Visits 3 and 2, the median change in the HJIG group was 0.25 [IQR: 0.00, 0.50], whereas for the placebo group, it was 0.00 [IQR: 0.00, 0.50]. For the comparison between Visits 4 and 2, the median change in the HJIG group was 0.25 [IQR: 0.08, 0.58], while in the placebo group, it was 0.05 [IQR: 0.00, 0.50]. The differences between the groups were statistically significant at both time points (*P* < 0.05), this indicates that HJIG treatment can effectively improve the erectile firmness of patients.

#### Percentage of IIEF-EF scores

IIEF-EF: When comparing results from Visit 4 to Visit 2, the HJIG group had an improvement rate of 50.00% [IQR: 33.33, 83.33], while the placebo group reported a rate of 18.75% [IQR: 4.17, 40.00]. The HJIG group’s performance was notably superior to the placebo group (*P* < 0.001).

#### Traditional Chinese medicine symptom scores

**FIGURE 10:**
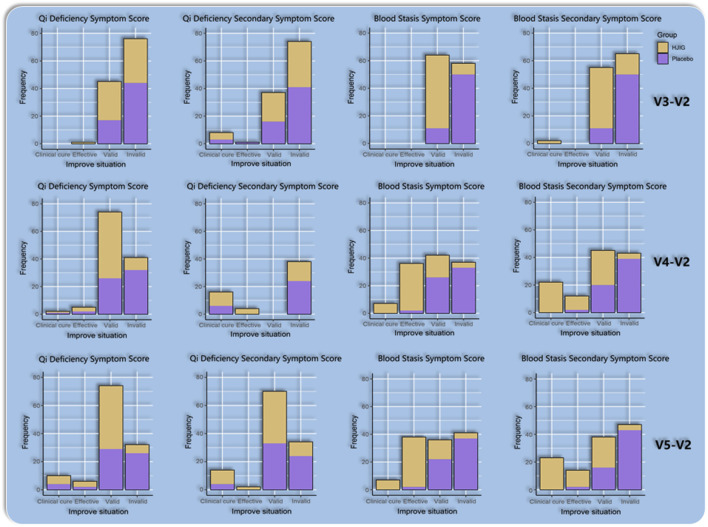
Improvement in traditional Chinese medicine symptom patterns.

At Visit 4, the effectiveness rate of the HJIG group was 85.2% for the primary symptom of Qi Deficiency and 75.0% for its secondary symptom. For the primary symptom of Blood Stasis, the effectiveness rate was 93.4%, and the rate was the same for its secondary symptom. This marked a significant improvement in the cure rate compared to Visit 3. Meanwhile, in the placebo group, the effectiveness rate for the primary symptom of Qi Deficiency stood at 47.5% and was 60.7% for its secondary symptom. For the primary symptom of Blood Stasis, the effectiveness rate reached 45.9%, while it was 36.1% for its secondary symptom. The difference in change values between the two groups, based on each visit point, was significant for all results, except for the secondary symptom score of Qi Deficiency at Visit 3 (*P* < 0.05 for all other outcomes) (Table 3; Table 4; Figure 10), this indicates that HJIG treatment improved the patients’ TCM symptoms.

**TABLE 3:** Changes in traditional Chinese medicine symptom scores at visits 3, 4, and 5 compared to visit 2 Qi Deficiency Symptom Score (v3∼5-v2).

	HJIG	placebo	*P*
n	61	61	
Qi Deficiency Symptom Score (v3-v2)	−4.00 [−4.00, −2.00]	−2.00 [−4.00, 0.00]	<0.001
Qi Deficiency Secondary Symptom Score (v3-v2)	−1.00 [−2.00, 0.00]	−1.00 [−1.00, 0.00]	0.661
Blood Stasis Symptom Score (v3-v2)	−8.00 [−10.00, −6.00]	−2.00 [-4.00, −1.00]	<0.001
Blood Stasis Secondary Symptom Score (v3-v2)	−2.00 [−2.00, −1.00]	0.00 [−1.00, 0.00]	<0.001
Qi Deficiency Symptom Score (v4-v2)	−6.00 [−8.00, −4.00]	−4.00 [−6.00, 0.00]	<0.001
Qi Deficiency Secondary Symptom (v4-v2)	−2.00 [−3.00, −1.00]	−1.00 [−2.00, −1.00]	0.034
Blood Stasis Symptom Score (v4-v2)	−14.00 [−18.00, −10.00]	−4.00 [−8.00, −2.00]	<0.001
Blood Stasis Secondary Symptom Score (v4-v2)	−3.00 [−4.00, −2.00]	−1.00 [−2.00, 0.00]	<0.001
Qi Deficiency Symptom Score (v5-v2)	−6.00 [−8.00, −6.00]	−4.00 [−6.00, −2.00]	<0.001
Qi Deficiency Secondary Symptom Score (v5-v2)	−2.00 [−3.00, −1.00]	−1.00 [−2.00, 0.00]	0.018
Blood Stasis Symptom Score (v5-v2)	−14.00 [−18.00, −10.00]	−4.00 [−6.00, −2.00]	<0.001
Blood Stasis Secondary Symptom Score (v5-v2)	−3.00 [−4.00, −2.00]	−1.00 [−2.00, 0.00]	<0.001

The difference in Qi deficiency primary symptom scores between visit 3–5 and visit 2, median [interquartile range]; Qi Deficiency Secondary Symptom Score (v3∼5-v2): The difference in Qi deficiency secondary symptom scores between visit 3 and visit 2, median [interquartile range]; Blood Stasis Symptom Score (v3∼5-v2): The difference in Blood stasis primary symptom scores between visit 3–5 and visit 2, median [interquartile range]; Blood Stasis Secondary Symptom Score (v3∼5-v2): The difference in Blood stasis secondary symptom scores between visit 3 and visit 2, median [interquartile range].

**TABLE 4:** Improvement in traditional Chinese medicine symptom patterns.

Visit/index	Group	Recover	Significant	Effective	Ineffective
V3 Qi Deficiency	HJIG	0	1	28	32
Symptom	PLACEBO	0	0	17	44
V4 Qi Deficiency	HJIG	1	3	48	9
Symptom	PLACEBO	1	2	26	32
V5 Qi Deficiency	HJIG	6	4	45	6
Symptom	PLACEBO	4	2	29	26
V3 Qi Deficiency	HJIG	5	0	21	34
Secondary Symptom	PLACEBO	3	1	16	41
V4 Qi Deficiency	HJIG	10	4	31	15
Secondary Symptom	PLACEBO	6	0	31	24
V5 Qi Deficiency	HJIG	10	3	33	14
Secondary Symptom	PLACEBO	4	1	28	28
V3 Blood Stasis	HJIG	0	0	53	8
Symptom	PLACEBO	0	0	11	50
V4 Blood Stasis	HJIG	7	34	16	4
Symptom	PLACEBO	0	2	26	33
V5 Blood Stasis	HJIG	7	36	14	4
Symptom	PLACEBO	0	2	22	37
V3 Blood Stasis	HJIG	2	0	44	15
Secondary Symptom	PLACEBO	0	0	11	50
V4 Blood Stasis	HJIG	22	10	25	4
Secondary Symptom	PLACEBO	0	2	20	39
V5 Blood Stasis	HJIG	23	12	22	4
Secondary Symptom	PLACEBO	0	2	16	43

#### Frequency of sexual activity and sexual satisfaction

**FIGURE 11:**
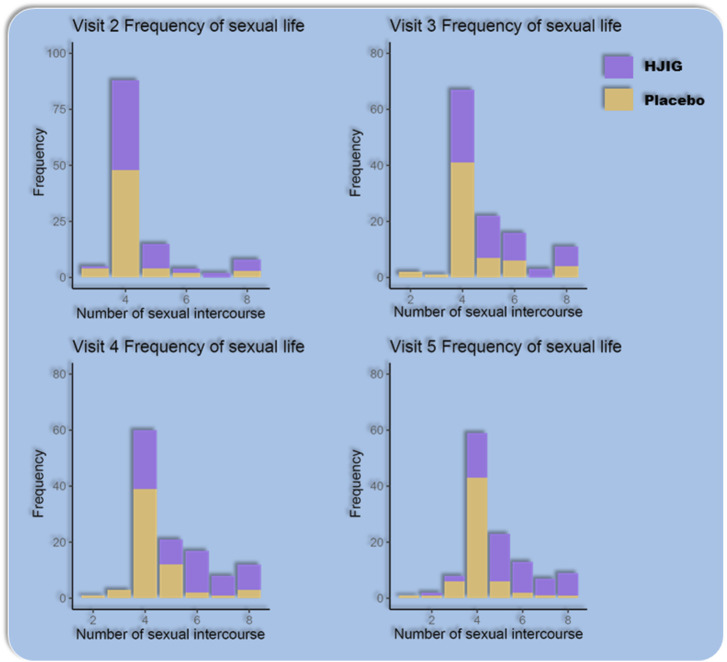
Frequency of sexual activity at different visit time points.

Sexual Activity Frequency: From visits 2–5, the distribution of sexual activity frequency among the subjects did not follow a normal distribution. Using the Wilcoxon rank-sum test for comparison between visit 2 and visits 3–5, there was a significant increase in the sexual activity frequency in the HJIG group during visits 3–5 compared to visit 2. In contrast, no significant difference was observed in the placebo group across these visits (Table 5; Figure 11).

**TABLE 5:** Frequency of sexual activity at different visit time points.

Visit	Overall		HJIG		placebo	
	M(Q_1_,Q_3_)	*p*	M(Q_1_,Q_3_)	*p*	M(Q_1_,Q_3_)	*p*
V2	4 (4,4)		4 (4,5)		4 (4,4)	
V3	4 (4,5)	0.0022	5 (4,6)	0.0062	4 (4,5)	0.1055
V4	4 (4,6)	0.0002	6 (4,7)	0.0001	4 (4,5)	0.1302
V5	4 (4,5)	0.0287	5 (4,6)	0.0004	4 (4,4)	0.5531

Sexual Satisfaction: At visit 4, the improvement level in sexual satisfaction for the HJIG group was 0.29 [IQR: 0.14, 0.60], while it was 0.25 [IQR: 0.00, 0.67] for the placebo group, showing no statistical significance between the groups. By visit 5, the median difference in the HJIG group was 0.55 [IQR: 0.40, 0.75] versus 0.00 [IQR: 0.00, 0.40] in the placebo group, highlighting a marked superiority of sexual satisfaction improvement in the HJIG group (*P* < 0.001) (Table 2).

This indicates that HJIG treatment can enhance patients’ sexual desire and improve their sexual experience.

#### Safety and adverse events

There were no significant differences in renal and liver function between and within the HJIG, and placebo groups after treatments. Most subjects well tolerated the research medication, and no serious adverse events were reported. The accusation report of HJIG can be found in Supplementary Data Sheet S3, and the metabolites of HJIG detected through LC-MS/MS of HJIG can be found in Supplementary Data Sheet S4. Using rat/human coefficients of 25.2, doses per kilogram of body weight of the rats were calculated based on the ratio of rat to human surface area: 11.34 g/kg. The rats (n = 50) were gavaged with this dose for 60 days, followed by an additional 14 days of observation. Over the total of 74 days, apart from some rats having diarrhea, no significant side effects were observed.

## Discussion

Herbal remedies and natural products have gained traction worldwide for treating erectile dysfunction (ED) (Magheli and Burnett, 2009; Feng et al., 2022), with substantial research underpinning their utility in recent years (Ciocanel et al., 2019; Wang et al., 2020; Wu et al., 2023). Traditional Chinese Medicine (TCM) originates from ancient China, with its primary therapeutic approach focusing on symptom differentiation and improving the overall health of the patient. Herbal formulation treatments constitute a significant component of TCM. Meta-analyses suggest that coupling Chinese herbs with PDE5i (phosphodiesterase-5 inhibitors) enhances efficacy without amplifying side effects (Wang et al., 2020). This corroborates the conclusions drawn from our preliminary studies on HJIG (Zhang G. et al., 2017; Ping et al., 2021). Another analysis indicates that singular Chinese herbal treatments can elevate IIEF (International Index of Erectile Function) scores and amplify clinical recovery rates (Wu et al., 2023). However, previous studies have touted the need for more rigorous clinical trials for Chinese herbal medicine (Xiong et al., 2014). Recognizing the pivotal role of accurate symptom differentiation in TCM and its association with ED, we advocate for randomized controlled trials (RCTs) targeting explicit symptom patterns to genuinely gauge Chinese medicine’s potency.

Our study indicates that exclusive use of HJIG can improve erectile function in patients with mild to moderate ED and enhance sexual completion (the ability to maintain an erection until ejaculation). Secondary outcomes also show that HJIG has the potential to increase sexual desire, as evidenced by an increase in sexual activity frequency. Clinical trail on herbal or herbal formula treatments for ED have yielded positive results (Kam et al., 2007; Shah et al., 2012; Xiong et al., 2014; Park et al., 2019).

A double-blind randomized controlled trial of a Thai herbal formulation composed of four medicinal plants showed improvements in IIEF-5 scores and sexual desire in patients with mild to moderate ED (Lin et al., 2022). Guojun et al.’s study indicated that acupuncture could improve IIEF-EF and SEP3 results in patients with psychogenic erectile dysfunction, although there was no significant difference in SEP2 between the treatment and placebo groups (Wang et al., 2023). A multicenter randomized controlled study provided a more detailed classification of patients and applied a TCM herbal formulation for those with liver depression and kidney deficiency symptoms related to psychogenic or mild arterial ED. By evaluating sexual success rates and erectile hardness in 500 patients, it was found that the herbal formulation Shugan Yiyang Capsule effectively improved erectile function and sexual completion (Qi et al., 2004). Nam Cheol Park et al. used a formula composed of five herbal granules for an 8-week intervention in ED patients. The results showed significant improvement in IIEF-EF scores in the treatment group compared to the placebo group, although there were no differences in SEP2 and SEP3 (Park et al., 2019). Gaurang R Shah used a mixture of eight herbal formulations to treat patients with mild to moderate ED, significantly improving their IIEF-EF scores. It is evident that the IIEF score remains a primary method for evaluating ED patients, especially in preliminary efficacy studies of new therapies (Shah et al., 2012).

Previous studies suggest that drugs like tadalafil are insufficient in alleviating auxiliary ED-related symptoms, whereas herbal interventions may fill this therapeutic gap (Zixue and Pengchao, 2018). This is a key advantage of herbal formulations. Therefore, in high-quality, large-scale studies focusing on other diseases, clinical research on traditional Chinese herbal formulas often incorporates TCM syndromes as criteria for inclusion, exclusion, or as a basis for evaluating treatment efficacy (Zhong et al., 2019). We adopted this model, and our results indicate significant improvement in patients’ specific TCM symptoms. Additionally, we used difference scores to evaluate treatment efficacy, which significantly reduces the impact of individual differences. By calculating the pre- and post-treatment differences, we can more accurately assess treatment effects, minimizing the influence of individual variability such as baseline levels. Furthermore, stratification results indicate that differences in patient age and duration of ED history may influence treatment efficacy. Older patients and those with a longer duration of ED may require longer treatment and recovery periods to achieve optimal therapeutic outcomes.

From a pharmacological perspective, the efficacy of HJIG may stem from the synergistic properties of its botanical drug metabolites. The main active metabolite in Rhodiola crenulata (Hook. f. et Thoms.) H. Ohba., salidroside, has been proven to improve hypoxic conditions (Zhao et al., 2020), inhibit fibrosis (Ma et al., 2020), and reduce phenotypic transformation of smooth muscle cells (Zhang X. et al., 2017). Astragali radix praeparata cum melle, a traditional herb used in ED treatment, though not fully explored mechanistically, has shown potential in improving cardiac diastolic function in postmenopausal women with hypertension, suggesting a similar beneficial effect on cavernous smooth muscle cells (Li et al., 2018). Salvia miltiorrhiza Bge. has been shown to inhibit oxidative stress and reduce apoptosis, thereby improving erectile function in diabetic rats (Zhang et al., 2018). Angelica sinensis (Oliv.) Diels has been found to enhance nitric oxide synthase (NOS) activity in rats with damaged cavernous nerves (Li et al., 2017). Cyathula officinalis Kuan has demonstrated efficacy in improving erectile function in diabetic rats (Wang et al., 2021). Lycium Chinense Miller. through its antioxidative stress properties improves erectile function in aged rats (Moon et al., 2017). Epimedium brevicornu Maxim. has been proven to induce erection in rats (Chen and Chiu, 2006), and its metabolites exhibit PDE5 inhibitory effects. Although not every herbs in the HJIG formulation has been extensively studied for its mechanism, most herbs have been proven beneficial in improving erectile function.

Our study, being exploratory in nature, still presents a range of aspects that necessitate further refinement and improvement. We lack bolstering through objective measures like NPTR (Nocturnal Penile Tumescence and Rigidity) or CT (Computed Tomography) tests. Additionally, even though five participants admitted to intermittent PDE5i usage and subsequently exited the study, there remains a latent ambiguity regarding undisclosed PDE5i usage, potentially skewing our results. The self-selection bias, where participants more inclined toward herbal treatment remain, while skeptics drop out, may also tilt outcomes.

In summary, HJIG demonstrates significant enhancements in sexual functionality for ED patients, complemented by improvements in their TCM symptomatology. We are planning broader real-world TCM studies to eliminate biases, reaffirm the therapeutic efficacy of HJIG, and extend the follow-up duration. Incorporating HJIG into a structured treatment regimen offers promise for a more favorable prognosis for patients. In future research endeavors, we are considering incorporating effectiveness parameters, such as time-efficiency and cost-effectiveness. The reduction in time and financial costs can alleviate patients’ burdens, increasing their engagement in the treatment, exemplifying one of the inherent benefits of herbal intervention.

We acknowledge that the sample size in this study is relatively small compared to large-scale clinical trials. However, given the exploratory nature of this research and the promising preliminary results, we believe the findings provide valuable insights into the efficacy of HJIG for mild to moderate ED. It is also important to note that collecting patients who meet the criteria for using Chinese herbal medicine alone, without involving ethical concerns, is particularly challenging. This recruitment difficulty further limits the sample size, but we are confident that the data gathered offers a solid foundation for future research. Larger studies with more extended follow-up periods are necessary to validate these findings and explore HJIG’s full therapeutic potential.

## Data Availability

The datasets presented in this article are not readily available. In consideration of patient privacy and the agreements signed, the data from this study is not permitted to be shared on public platforms. Researchers with relevant needs can contact the corresponding author of this article for access.

## Abbreviations

HIJG, Hongjing I granule; ED, Erectile dysfunction; TCM, traditional Chinese medicine; IIEF-EF, International Index of Erectile Function-Erectile Function domain; SEP, Sexual Encounter Profile; EF, erectile function; EHS, erection hardness scale; AEs, Adverse events; CRF Case report form.
