# Supplementary material for: Efficacy of Hongjing I granule, an herbal medicine, in patients with mild to moderate erectile dysfunction in a randomized controlled trial
Source: Front Pharmacol. 2024 Dec 24;15:1367812. doi: 10.3389/fphar.2024.1367812 (PMC11703738; doi:10.3389/fphar.2024.1367812)
Supplement: Supplementary file 1 [file DataSheet2.pdf]

浙江中医药大学附属第二医院伦理委员会

伦理审查批件

批件号：2019-KL-012-01

审查日期、地点	2019 年 4 月 22 日　杭州市文二路 8 号 241 会议室		
临床研究批文	——	项目类别	科研项目
临床研究项目	基于循证医学证据的阳痿病中医药防治机制研究		
审查文件	1、科研项目审查申请报告　2、临床研究方案<br>3、研究者专业履历　4、临床研究者岗位职责<br>5、知情同意书（受试者）　6、知情同意书（受试者性伴侣）<br>7、附件：情况补充说明、2017 年伦理意见		
临床研究主要负责单位	泌尿外科	主要研究者	吕伯东
伦理审查方式	■ 会议审查　□ 加快审查		
投票结果	委员人数 17 人，本次会议出席委员 9 人，参加投票委员 9 人，同意 9 票，修改后同意 0 票，修改后重审 0 票，不同意 0 票，终止或暂停已批准的试验 0 票。		
审查意见	**审查结果：■同意　□不同意**<br>根据卫生部《涉及人的生物医学研究伦理审查办法（试行）》（2007）、CFDA《药物临床试验质量管理规范（2003）》、《医疗器械临床试验规定（2004）》、WMA《赫尔辛基宣言》和 CIOMS《人体生物医学研究国际道德指南》的伦理原则，经本伦理委员会审查，同意按所批准的临床研究方案、知情同意书、招募材料开展本项研究。请遵循 GCP 原则、遵循伦理委委员会批准的方案开展临床研究，保护受试者的健康与权力。<br>◆ 研究开始前，请申请人完成临床试验注册。<br>◆ 研究过程中若变更主要研究者，对临床方案、知情同意书、招募材料等的任何修改，请申请人提交修正案审查申请。<br>◆ 发生严重不良事件（SAE），请申请人及时提交 SAE 报告，本伦理委员会有权根据 SAE 做出新的审查决定；提交报告之后，尽快提交详细的严重不良事件随访报告。<br>◆ 请按照伦理委员会规定的持续审查频率，申请人在截止日期前 1 个月提交持续审查申请报告，本伦理委员会有权根据实际开展情况修改持续审查频率，并有权根据实际审查情况做出新的决定；当出现任何可能显著影响实验进行、或增加受试者危险的情况时，请申请人及时向伦理委员会提交书面报告。<br>◆ 研究纳入了不符合纳入标准或符合排除标准的受试者，符合终止试验规定而未让受试者退出研究，给予错误治疗或剂量，给予方案禁止的合并用药等没有遵从方案的情况；或可能对受试者的权益/健康、以及研究的科学性造成不良影响等违背 GCP 原则的情况，请申办方/监察员/研究者提交违背方案报告。<br>◆ 申请人暂停或提前终止临床研究，请及时提交暂停/终止研究报告。<br>◆ 完成临床研究，请申请人提交结题报告。<br>◆ 及时书面报告中心伦理的重要决定。<br>◆ 凡涉及中国人类已遗传资源、需要报批的研究项目，需要在获得中国人类遗传资源管理办公室批准后才能开始研究。		
有效期	研究结束	持续审查频率	□ 3 个月　□ 6 个月　■ 12 个月　其他
主任委员签字	陈卫建	浙江中医药大学附属第二医院<br>伦理委员会（盖章）<br>日期：2019 年 4 月 23 日	

地址：杭州市拱墅区潮王路 318 号　邮编：310005　电话：0571-85267016　联系人：娄懿

浙江省中医药防治重大疾病攻关计划
项目合同书

项目编号：2018ZY007

项目名称：基于循证医学证据的阳痿病中医药防治机制研究

项目委托单位：浙江省卫生计生委

项目承担单位：浙江中医药大学附属第二医院

项目负责人：吕伯东

起止时间：2017 年 9 月至 2022 年 8 月

浙江省卫生计生委

2017 年制

Zhejiang Chinese Medicine University Affiliated Second Hospital Ethics Committee Ethical Review Approval

Approval No.: 2019-KL-012-01

**Review Date and Location**	April 22, 2019.<br>Room 241, No. 8 Wen'er Road, Hangzhou		
**Clinical Research Approval Document**		**Project Category**	Scientific Research Project
**Clinical Research Project**	Research on the Prevention and Treatment Mechanism of Yang's Disease Based on Evidence-Based Medicine		
**Review document**	1. Scientific Research Project Review Application Report<br>2. Clinical Research Plan<br>3. Professional Resume of Researchers<br>4. Job Responsibilities of Clinical Researchers Review Documents<br>5. Informed Consent Form (Subjects)<br>6. Informed Consent Form (Subjects' Sexual Partners)<br>7. Attachment: Supplementary Information, Ethical Opinions of 2017		
**Main Clinical Research Responsible Unit**	Urology Department	**Principal Investigator**	Bodong Lv
**Ethical Review Method**	■ Meeting Review □ Expedited Review		
Poll results	Number of Committee Members: 17, Number of Attending Members: 9, Number of Voting Members: 9, Agree: 9 votes, Post-Modification Agreement: 0 votes, Post-Modification Re-review: 0 votes, Disagreement: 0 votes, Termination or Suspension of Approved Experiments: 0 votes.		
review comment	Review Result: ■ Agreement □ Disagreement<br>In accordance with the ethical principles of the Ministry of Health's "Ethical Review Methods for Biomedical Research Involving Humans (Trial)" (2007), CFDA "Good Clinical Practice for Drug Clinical Trials (2003)" "Regulations on Medical Device Clinical Trials (2004)", WMA "Helsinki Declaration" and CIOMS International Ethical Guidelines for Biomedical Research Involving Humans, this ethics committee has reviewed and agreed to carry out this research according to the approved clinical research plan, informed consent form,		

	and recruitment materials. Please follow the GCP principles and the approved plan of the ethics committee to conduct clinical research and protect the health and rights of the subjects.<br>※Before the start of the research, please complete the clinical trial registration.<br>※During the research process, if there are any changes to the principal investigator, the clinical plan, informed consent form, recruitment materials, etc., please submit a revised review application.<br>※In the event of a serious adverse event (SAE), please submit an SAE report in a timely manner, and this ethics committee has the right to make a new review decision based on the SAE: After submitting the report, please submit a detailed follow-up report of the serious adverse event as soon as possible.<br>※Please submit a continuous review application report 1 month before the termination date according to the frequency of continuous review specified by the ethics committee. This ethics committee has the right to modify the frequency of continuous review according to the actual situation and make new decisions based on the actual review situation: When any situation that may significantly affect the experiment or increase the risk to the subjects occurs, please submit a written report to the ethics committee in a timely manner.<br>※If the research includes subjects who do not meet the inclusion criteria or meet the exclusion criteria, and if the subjects are not withdrawn from the research in accordance with the termination of the experiment regulations, and if incorrect treatment or dosage is given, or if prohibited concurrent medication is given in violation of the plan, etc., please submit a deviation report from the plan by the sponsor/monitor/researcher.<br>※If the applicant suspends or terminates the clinical research in advance, please submit a suspension/termination research report in a timely manner.<br>※After completing the clinical research, please submit a final report.<br>※Report important decisions of the central ethics in writing in a timely manner.<br>※For any research projects involving Chinese human genetic resources that require approval, the research

	can only begin after obtaining approval from the Office of Chinese Human Genetic Resources Management.		
**Validity Period**	End of study	**Frequency of Continuous Review**	□ 3 months □ 6 months ■ 12 months
**Signature of the chairman**			

Zhejiang Chinese Medicine Prevention and Treatment of Major Diseases Research Program Project Contract

Project No.: 2018ZY007

Project Name: Research on the Prevention and Treatment Mechanism of Yang's Disease Based on Evidence-Based Medicine

Project Entrusting Unit: Zhejiang Provincial Department of Health and Family Planning

Project Undertaking Unit: Zhejiang Chinese Medicine University Affiliated Second Hospital

Project Leader: Bodong Lv

Start and End Time: September 2017 to August 2022

Zhejiang Provincial Health and Family Planning Commission

Made in 2017
