# Supplementary material for: Efficacy of Hongjing I granule, an herbal medicine, in patients with mild to moderate erectile dysfunction in a randomized controlled trial
Source: Front Pharmacol. 2024 Dec 24;15:1367812. doi: 10.3389/fphar.2024.1367812 (PMC11703738; doi:10.3389/fphar.2024.1367812)
Supplement: Supplementary file 2 [file DataSheet4.pdf]

1. Fingerprint Profile of a Single Granule

红景天 (Hong-Jing-Tian) - Rhodiola crenulata (Hook. f. et Thoms.) H. Ohba [Crassulaceae; Rhodiola crenulata (Hook. f. et Thomas.) H. Ohba]

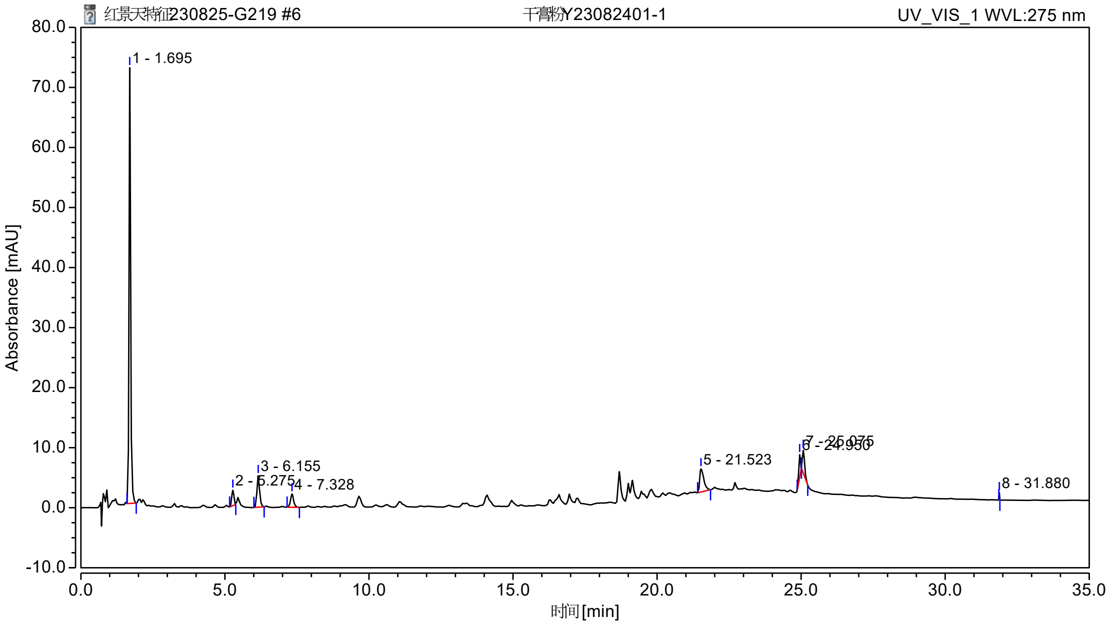

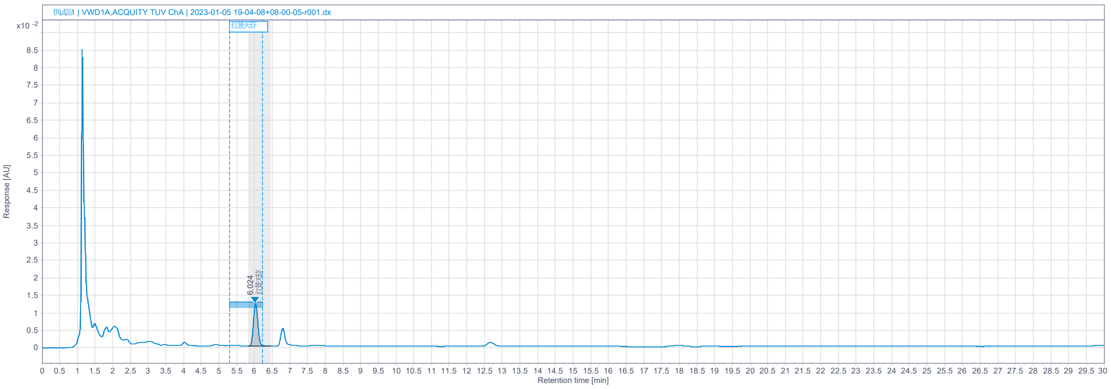

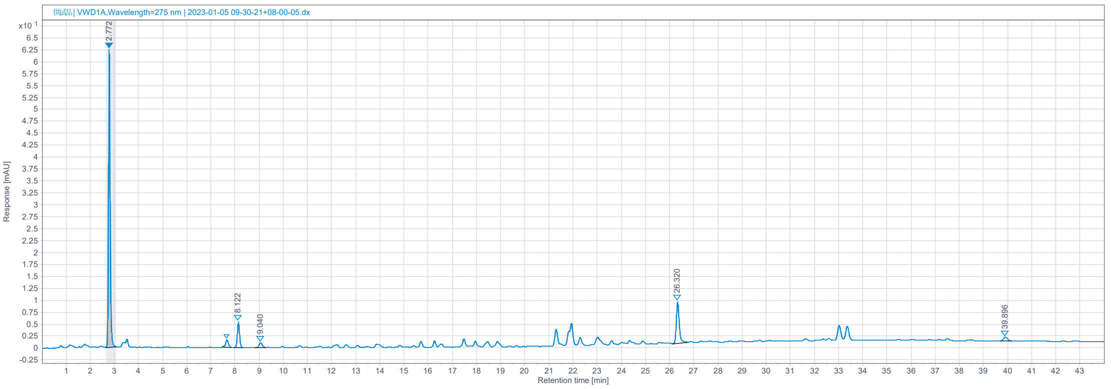

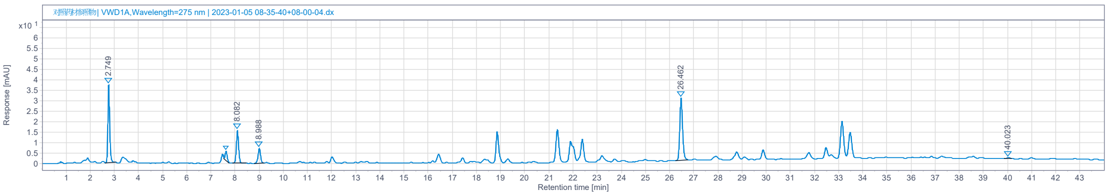

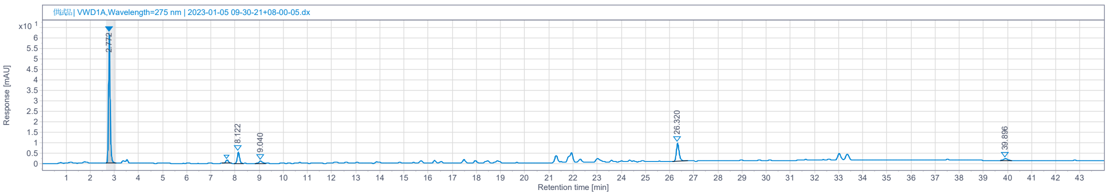

黄芪 (Huang-Qi) - Astragalus mongholicus Bunge [Fabaceae; astragali radix praeparata cum melle]

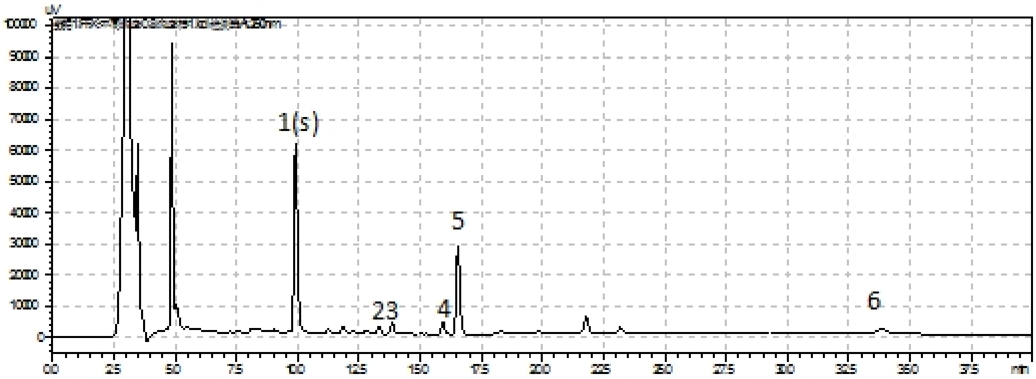

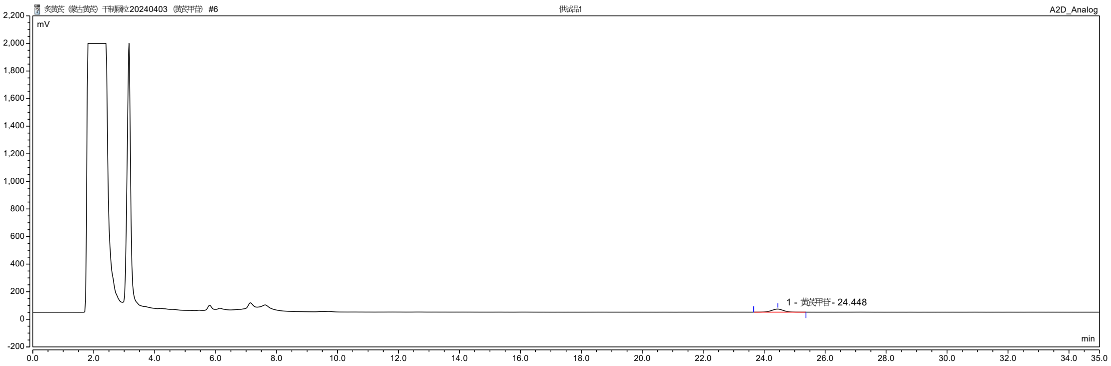

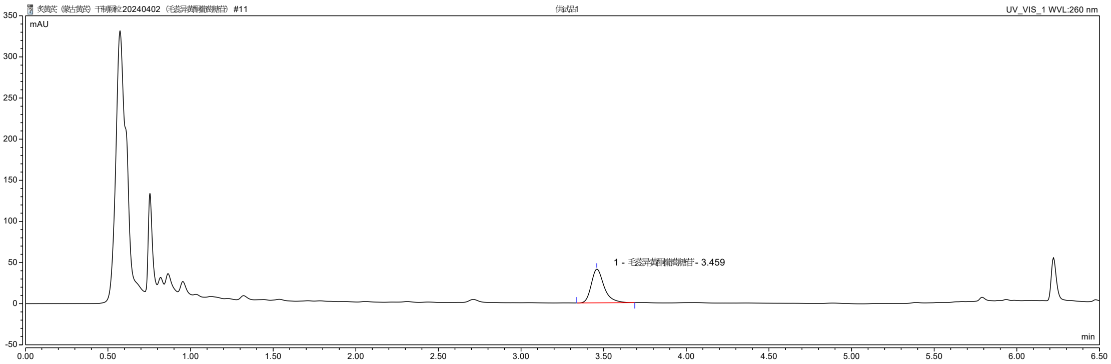

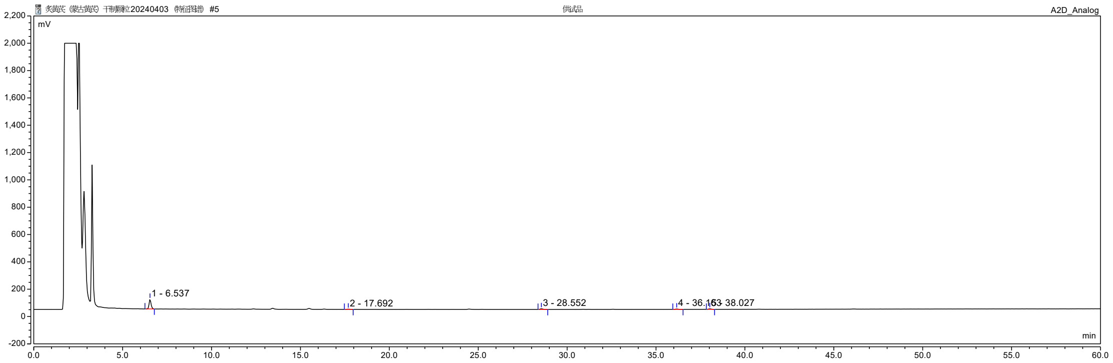
A2D_Analog
mV
1 - 6.537
2 - 17.692
3 - 28.552
4 - 36.163
38.027
min

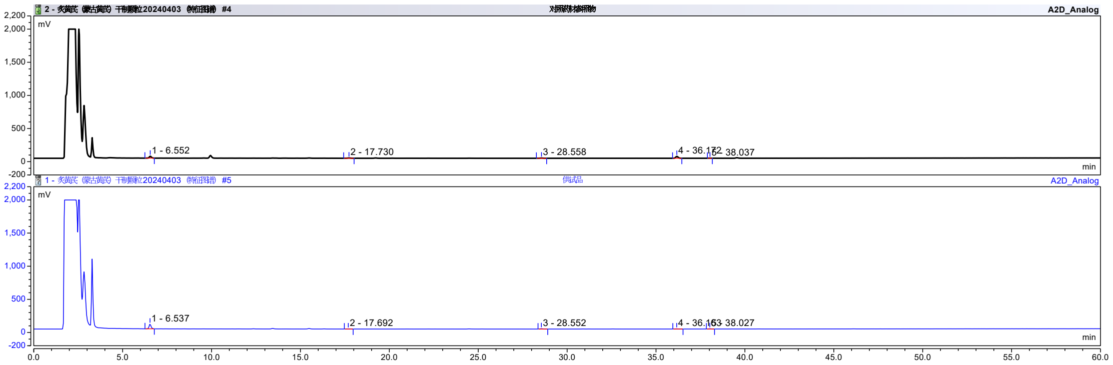
A2D_Analog
mV
1 - 6.552
2 - 17.730
3 - 28.558
4 - 36.172
38.037
1 - 6.537
2 - 17.692
3 - 28.552
4 - 36.163
38.027
min

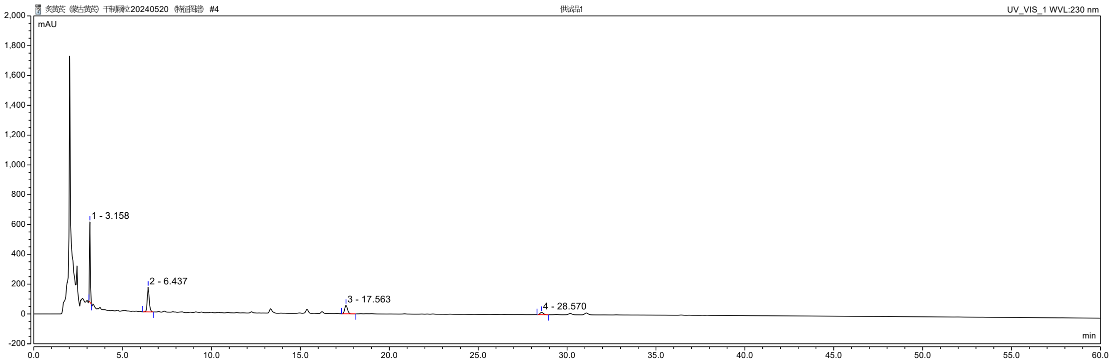
UV_VIS_1 WVL:230 nm
mAU
1 - 3.158
2 - 6.437
3 - 17.563
4 - 28.570
min

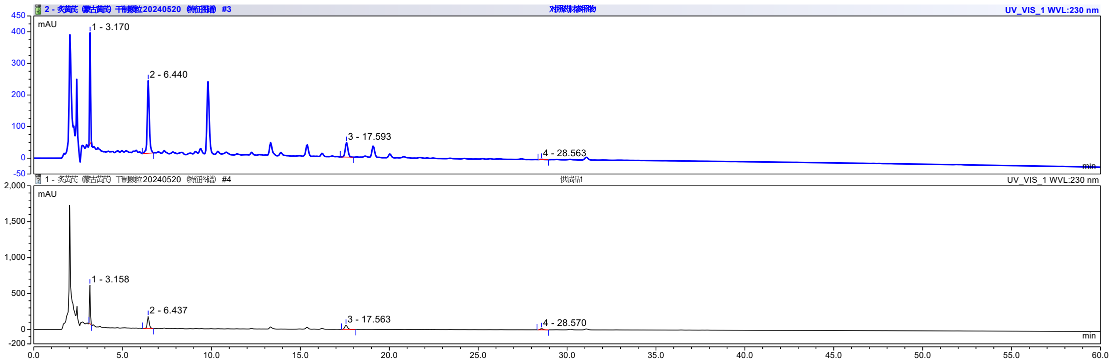
UV_VIS_1 WVL:230 nm
mAU
1 - 3.170
2 - 6.440
3 - 17.593
4 - 28.563
1 - 3.158
2 - 6.437
3 - 17.563
4 - 28.570
min

党参 (Dang-shen) - Codonopsis pilosula (Franch.) Nannf. [Campanulaceae; Codonopsis pilosula (Franch.) Nannf.]

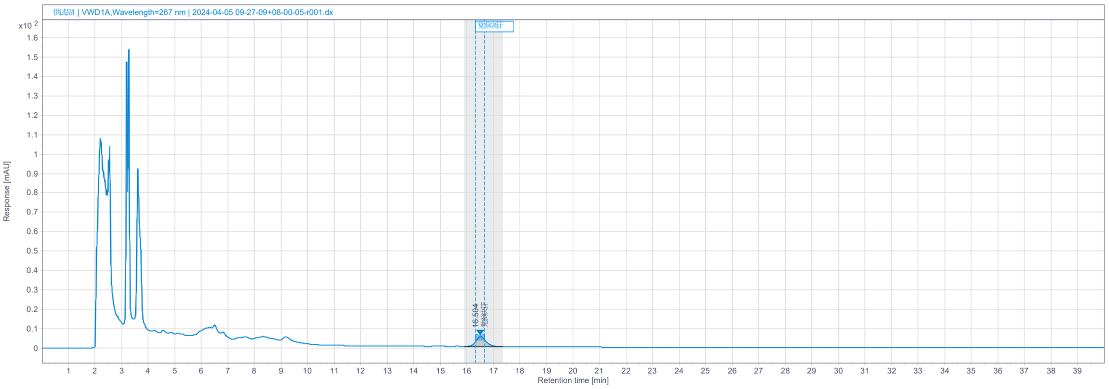

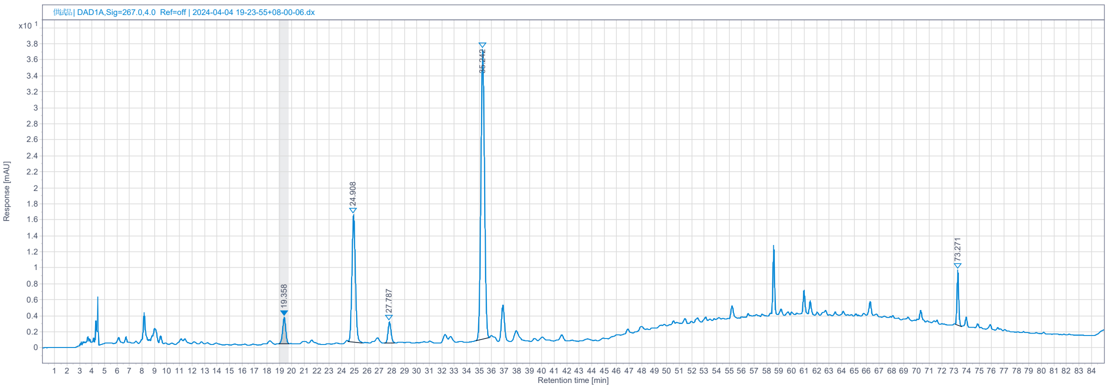

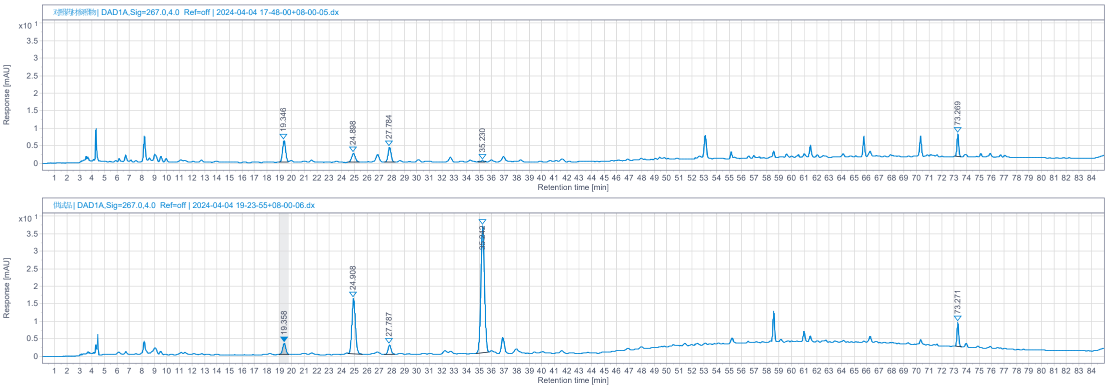

全当归 (Quan-Dang-Gui) – Angelica sinensis (Oliv.) Diels [Apiaceae; Angelica sinensis (Oliv.) Diels]

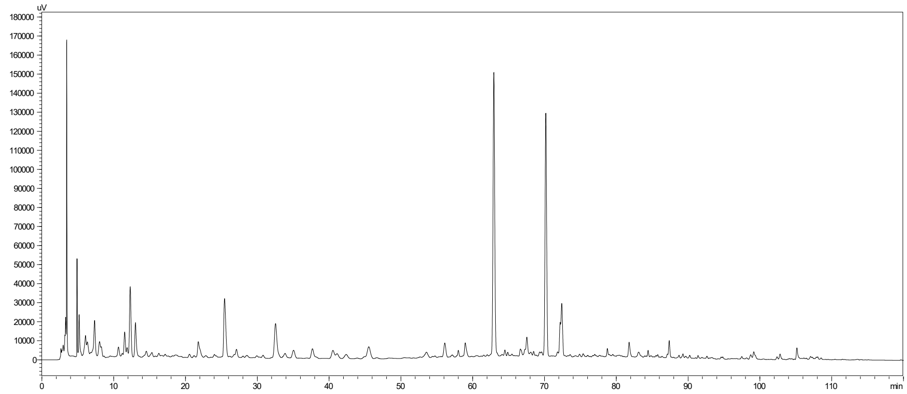

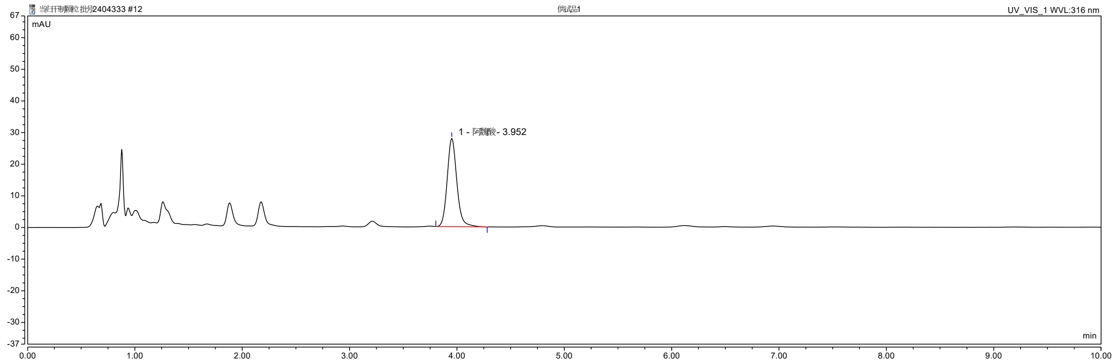

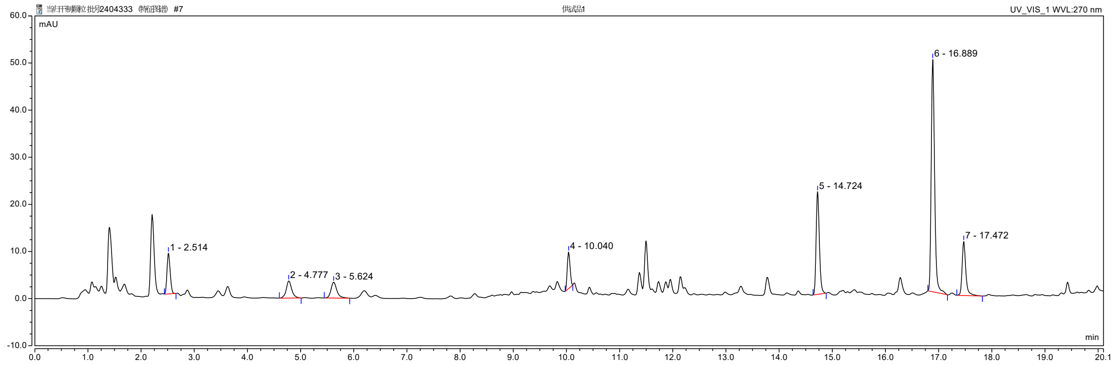

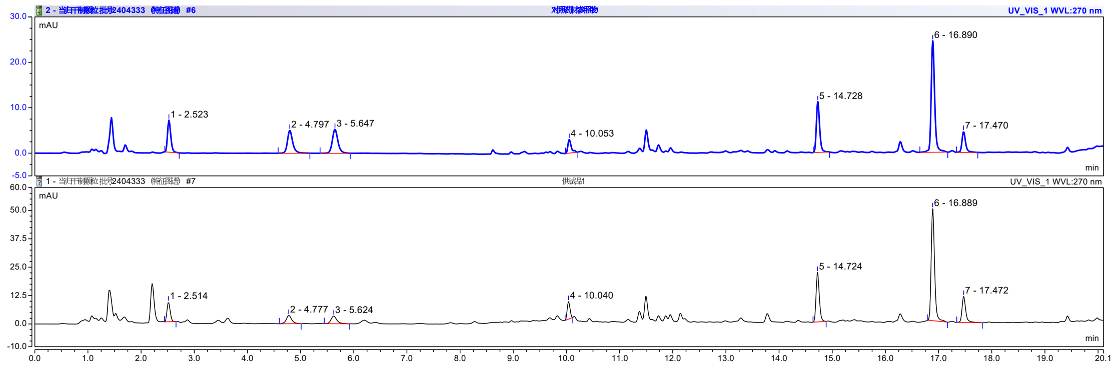

丹参 (Dan-shen) - Salvia miltiorrhiza Bunge [Lamiaceae; Salvia miltiorrhiza Bge.]

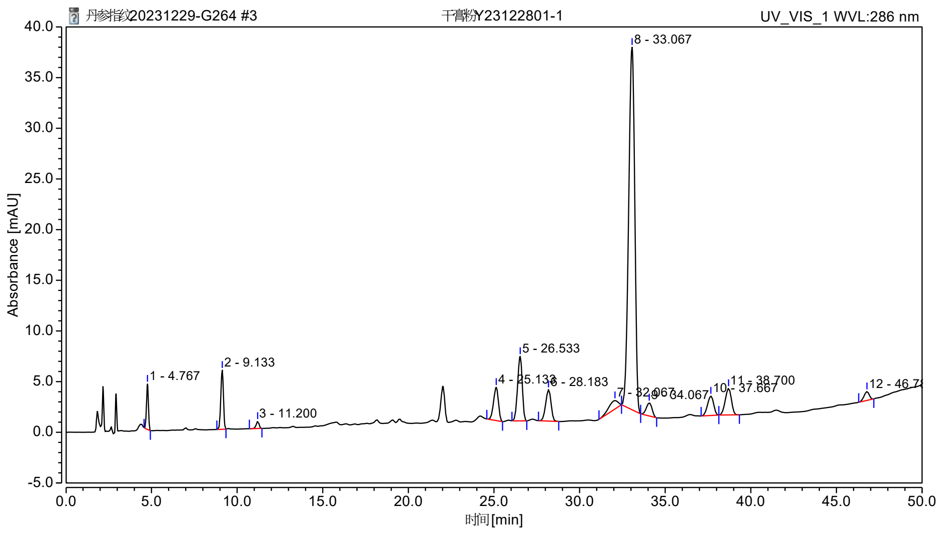

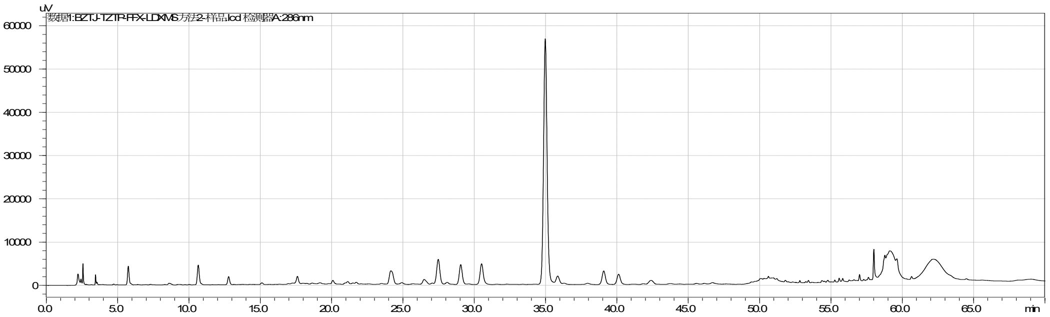

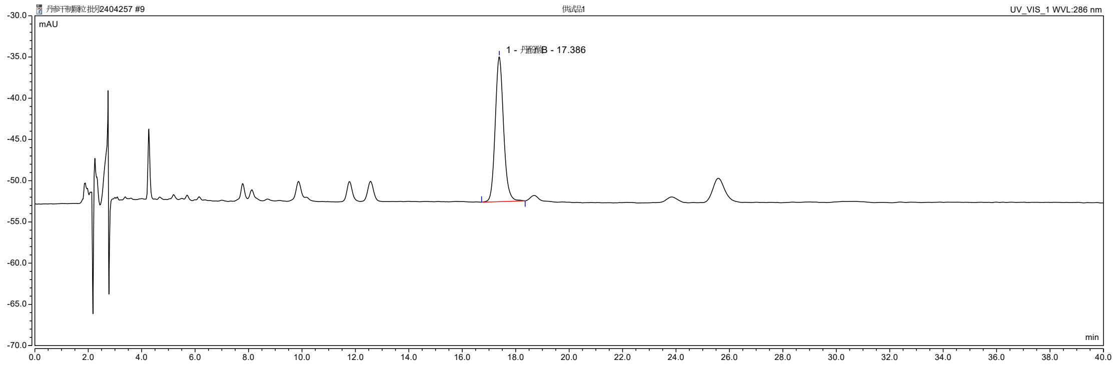

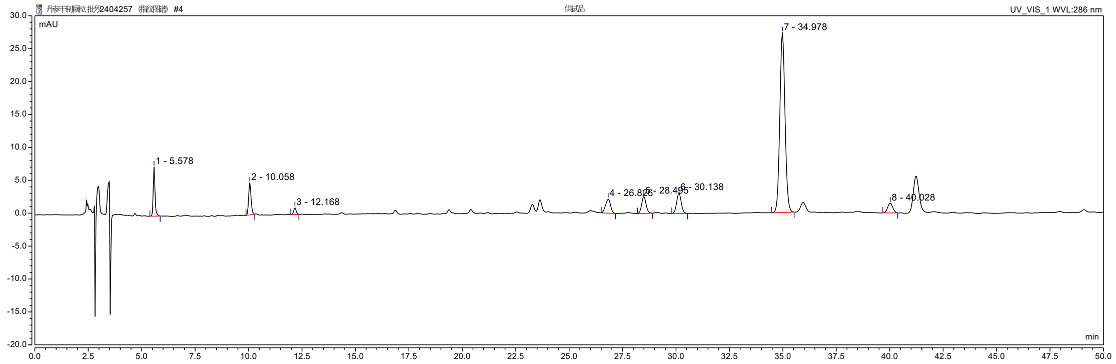

白芍 (Bai-Shao) - Paeonia lactiflora Pall. [Paeoniaceae; Paeonia lactiflora Pall.]

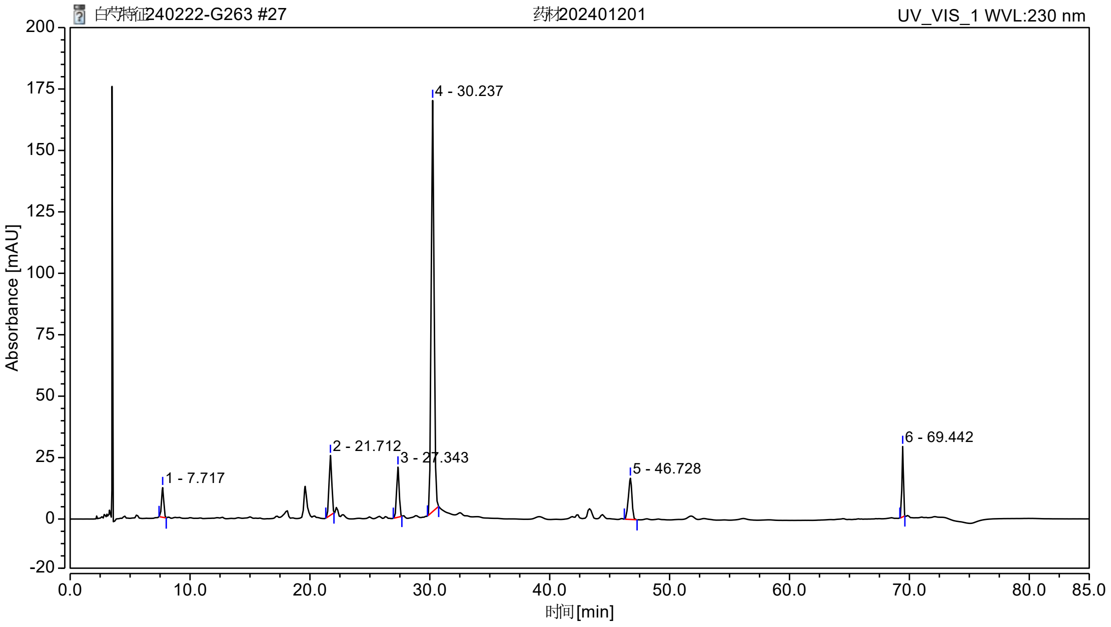

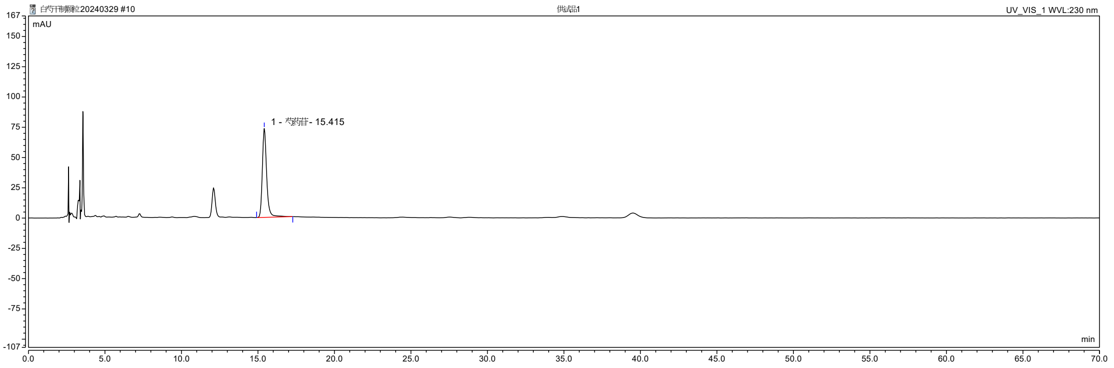

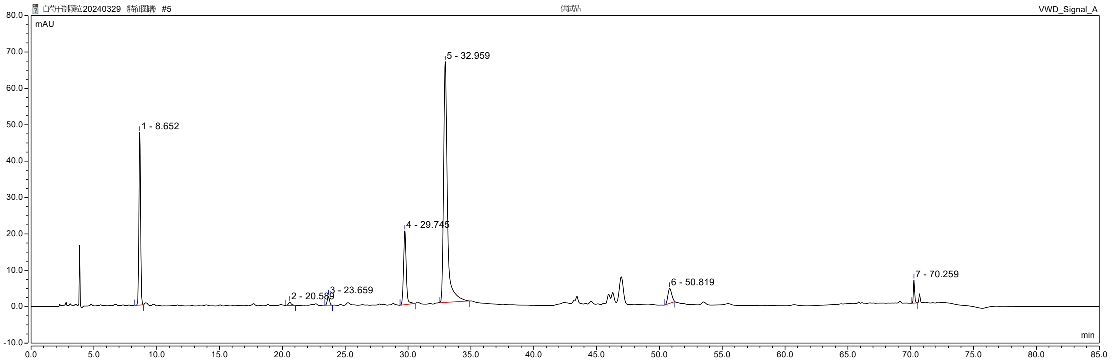

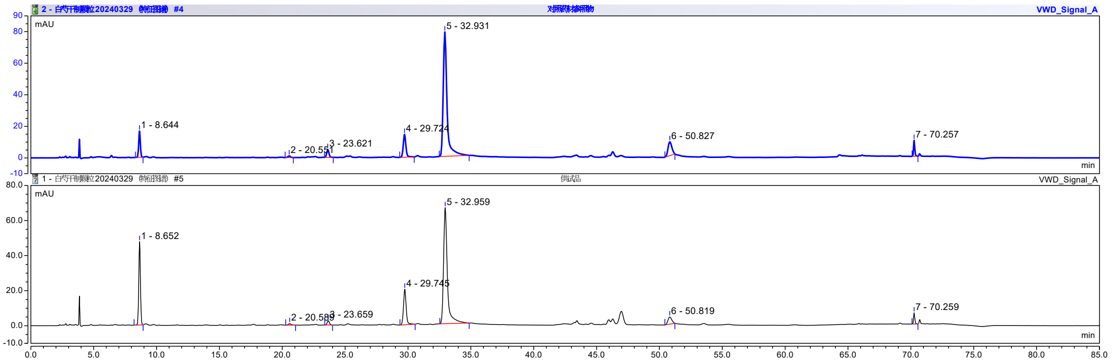

枸杞 (Gou-Qi) - Lycium chinense Mill. [Solanaceae; Lycium chinense Mill.]

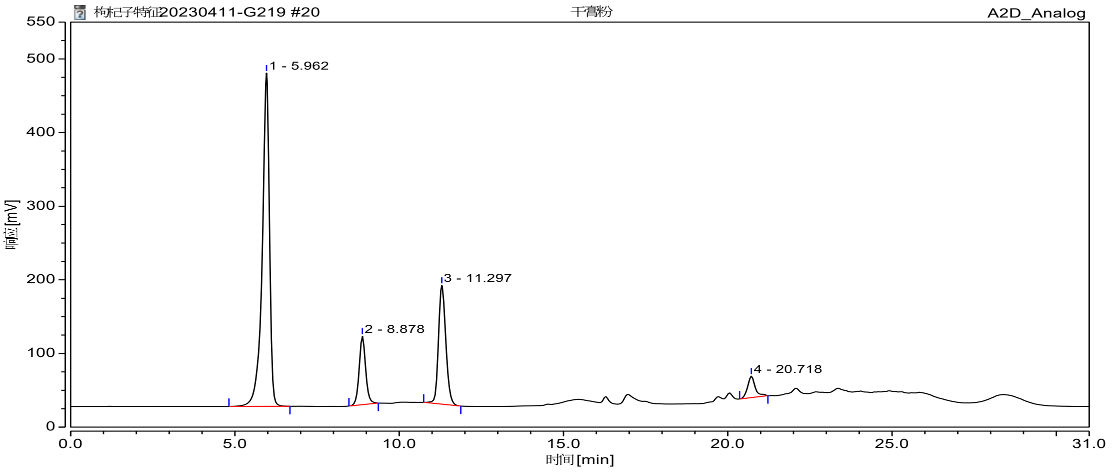

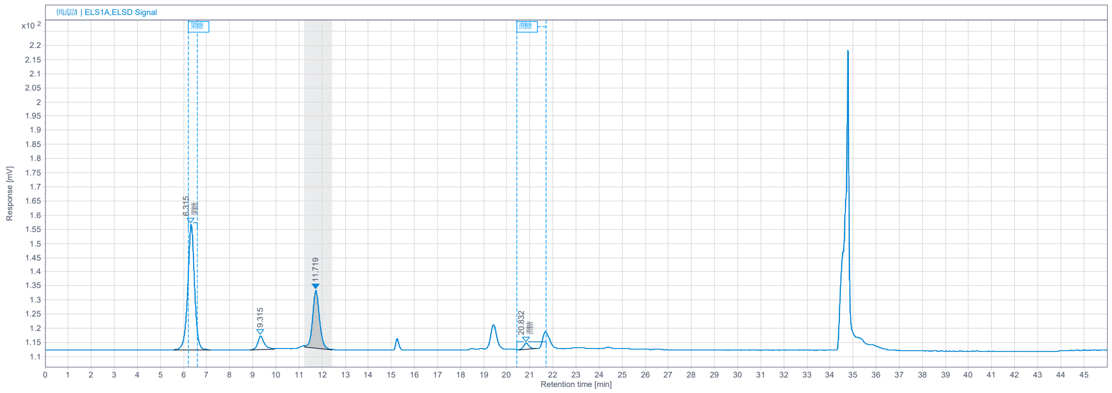

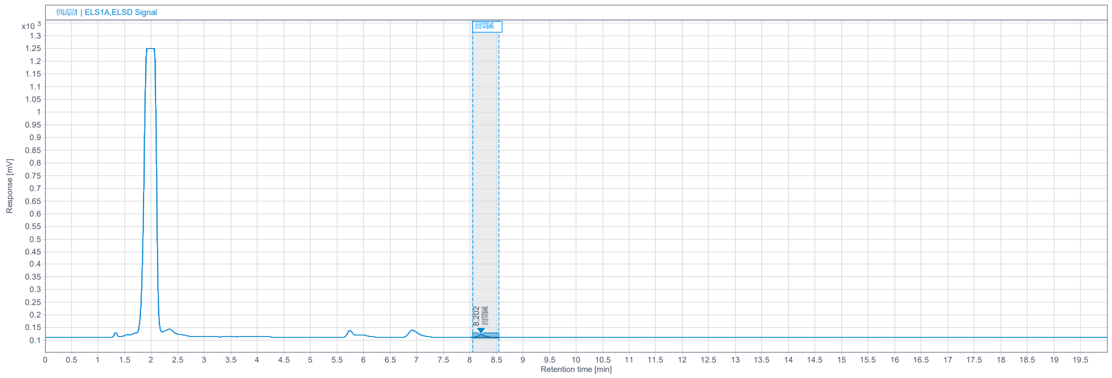

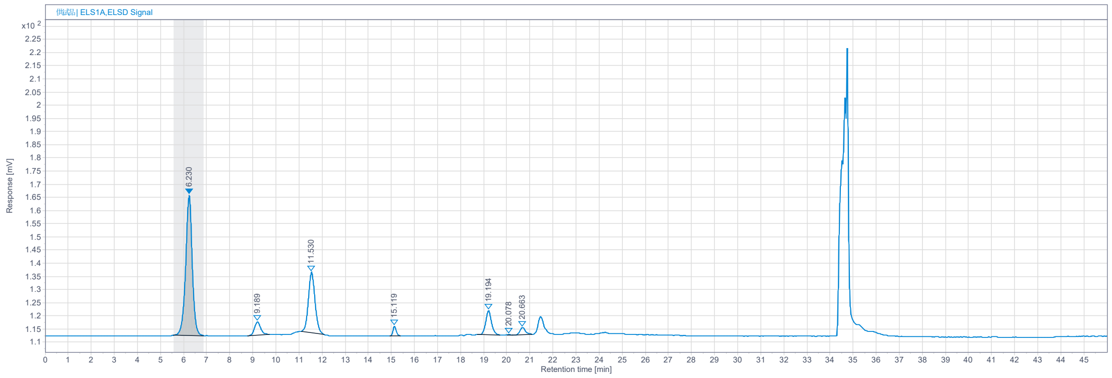

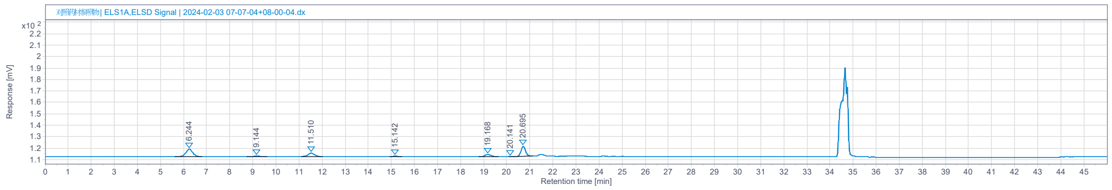

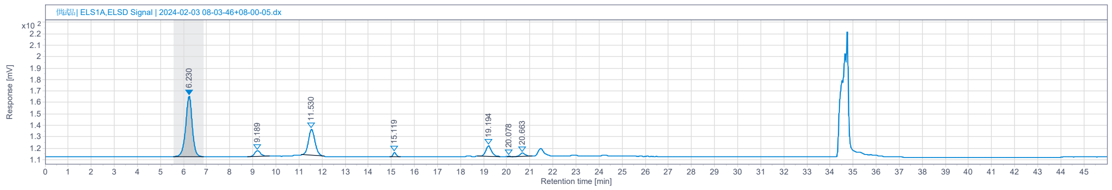

淫羊藿（Yin-Yang-Huo) - Epimedium brevicornu Maxim. [Berberidaceae; Epimedium brevicornu Maxim.]

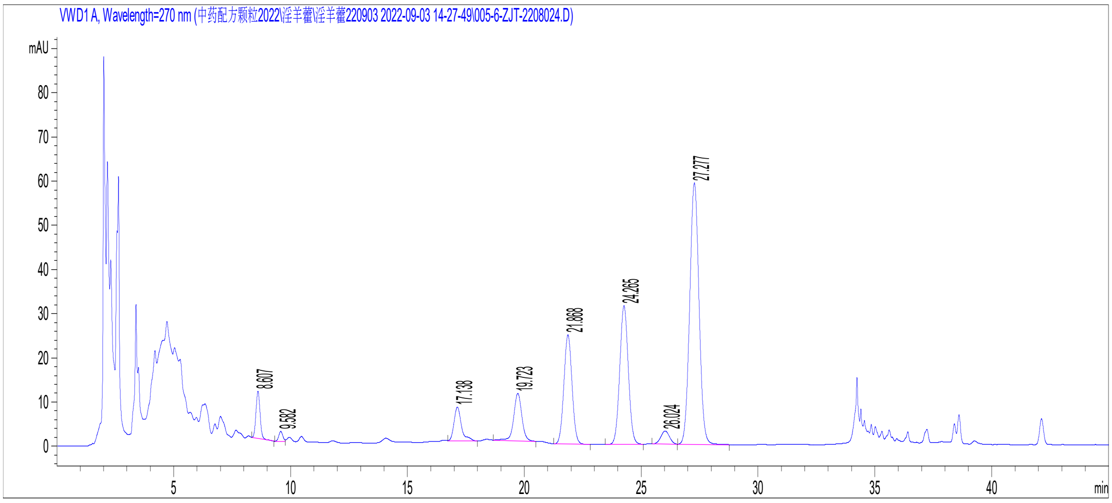

淫羊藿(淫羊藿)--淫羊藿（淫羊藿）干制颗粒 批号 2401020

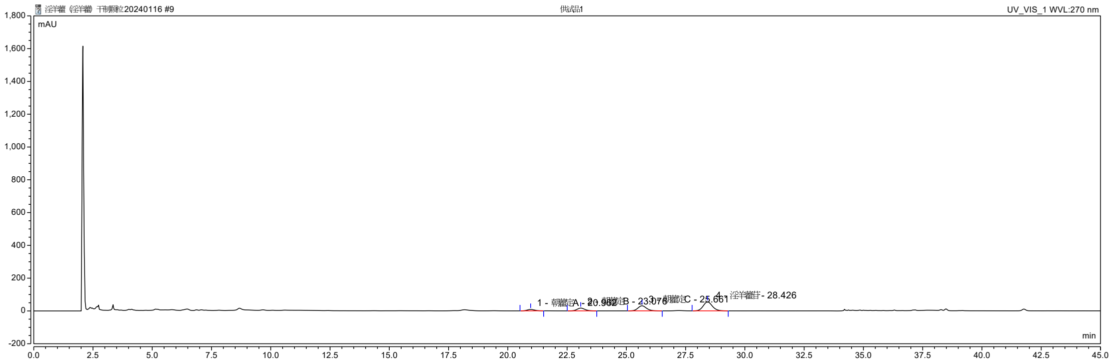

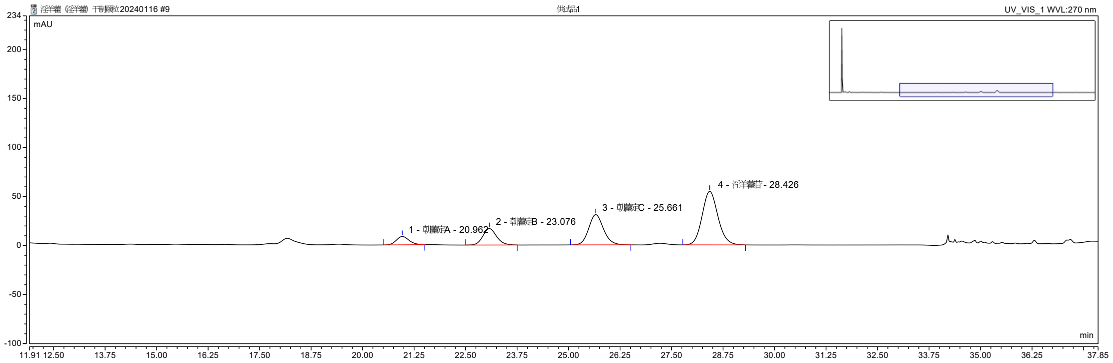

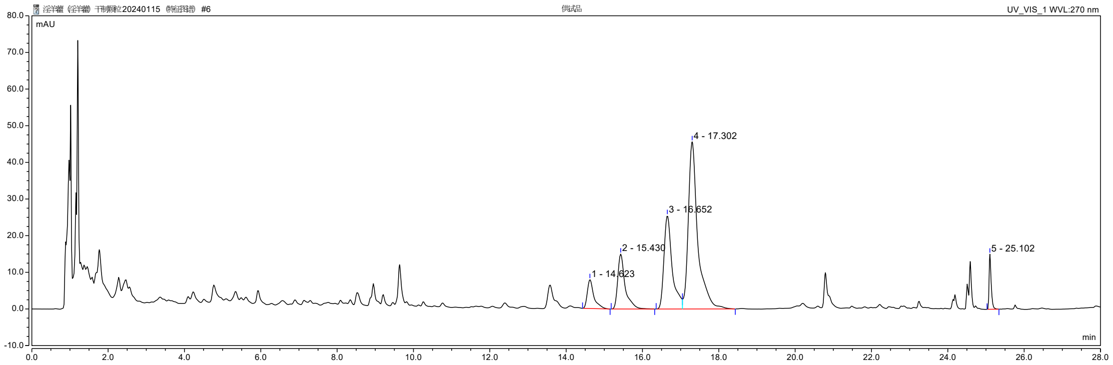

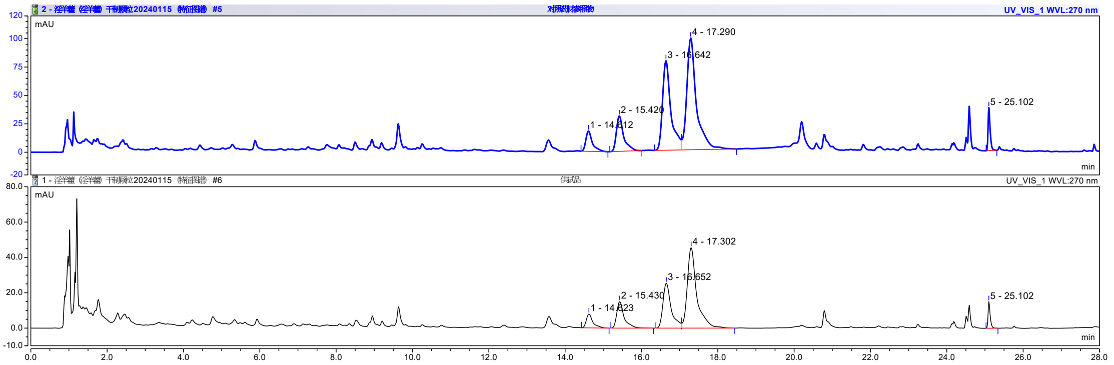

川牛膝（Chuan-Niu-Xi) - Cyathula officinalis K.C.Kuan [Amaranthaceae; Cyathula officinalis Kuan]

数据文件名:CNX-DGF-TZTP-MS-乙腈-0.1%甲酸-方法-7-乙酸乙酯萃取2次-Xtimate.lcd

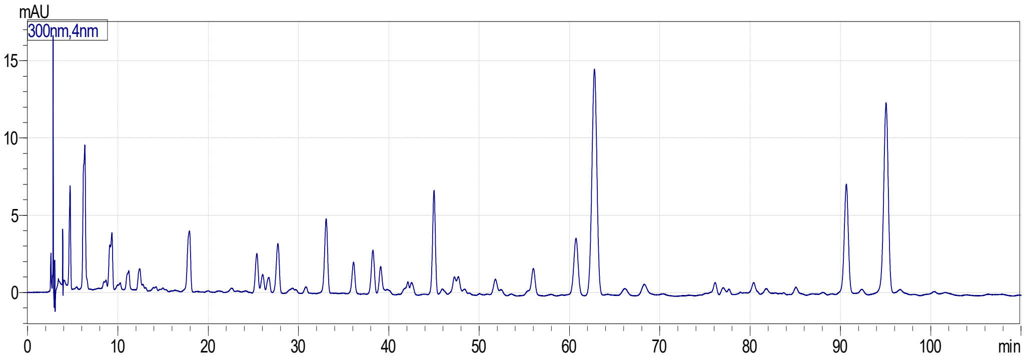

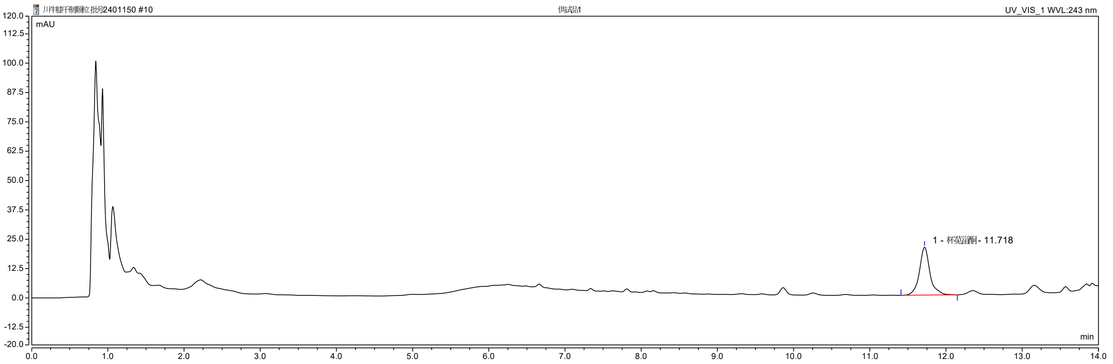

特征图谱

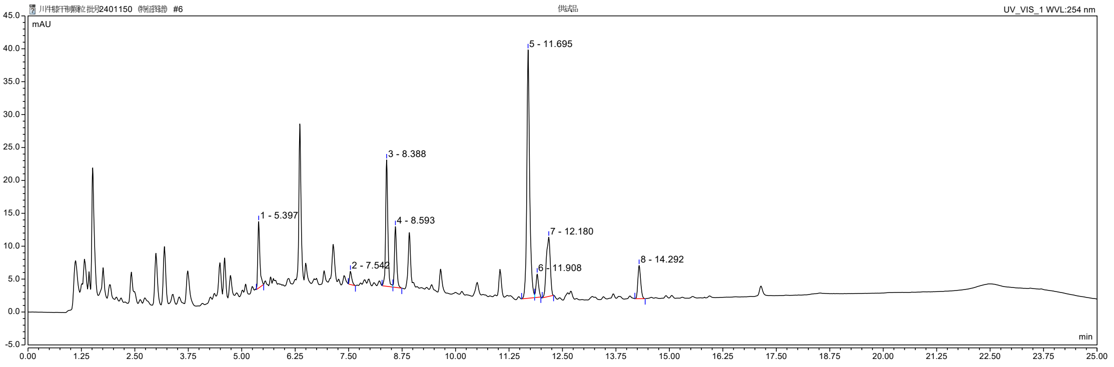

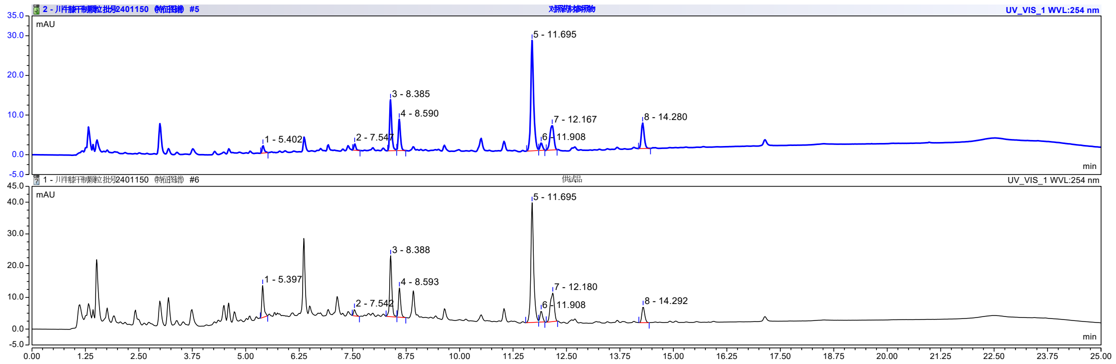

2. Fingerprint Profile of the Hongjing I Granule

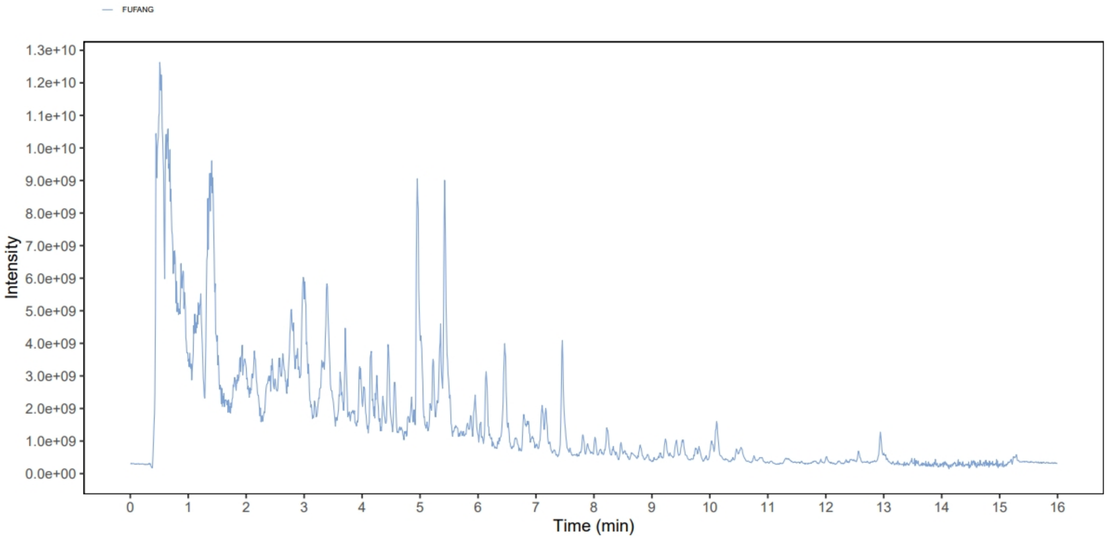

NEG

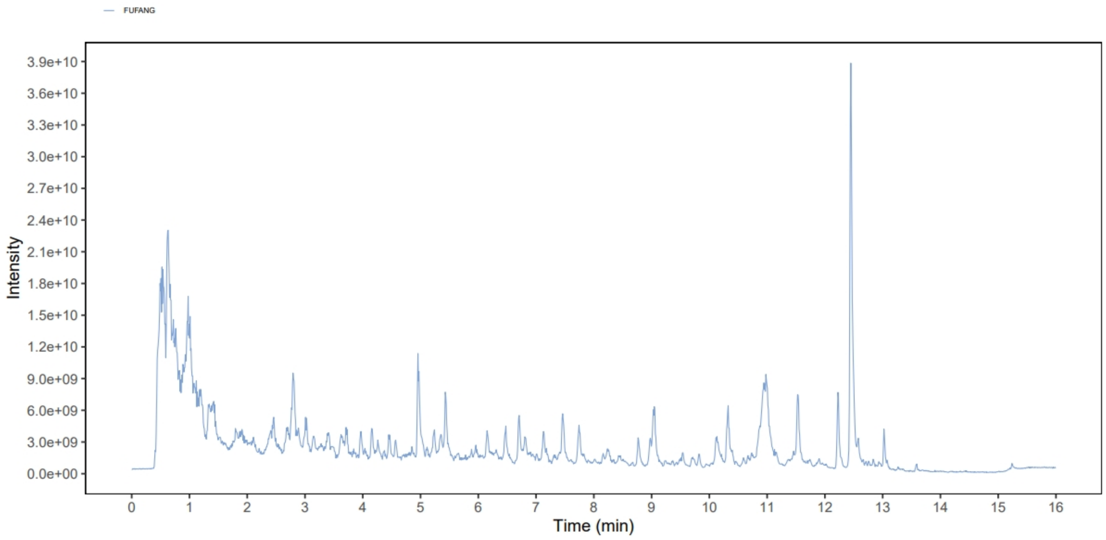

POS
