# Supplementary material for: Efficacy of Hongjing I granule, an herbal medicine, in patients with mild to moderate erectile dysfunction in a randomized controlled trial
Source: Front Pharmacol. 2024 Dec 24;15:1367812. doi: 10.3389/fphar.2024.1367812 (PMC11703738; doi:10.3389/fphar.2024.1367812)
Supplement: Supplementary file 3 [file DataSheet3.pdf]

Chinese name	Botanical name	Part used	Granule dosage
红景天 Hong-Jing-Tian	Rhodiola crenulata (Hook. f. et Thoms.) H. Ohba	Dried Root	1.5g
黄芪 Huang-Qi	Astragalus mongholicus Bunge	Dried Root	6g
党参 Dang-shen	Codonopsis pilosula (Franch.) Nannf.	Dried Root	4.5g
全当归 Quan-Dang-Gui	Angelica sinensis (Oliv.) Diels	Dried Root	4.8g
丹参 Dan-shen	Salvia miltiorrhiza Bunge	Dried Root	3g
白芍 Bai-Shao	Paeonia lactiflora Pall.	Dried Root	1.5g
枸杞 Gou-Qi	Lycium chinense Mill.	Dried Fruit	6g
淫羊藿 Yin-Yang-Huo	Epimedium brevicornu Maxim.	Dried Stems and Leaves	0.5g
川牛膝 Chuan-Niu-Xi	Cyathula officinalis K.C.Kuan	Dried Root	3.6g

Table 1 Composition of HJIG

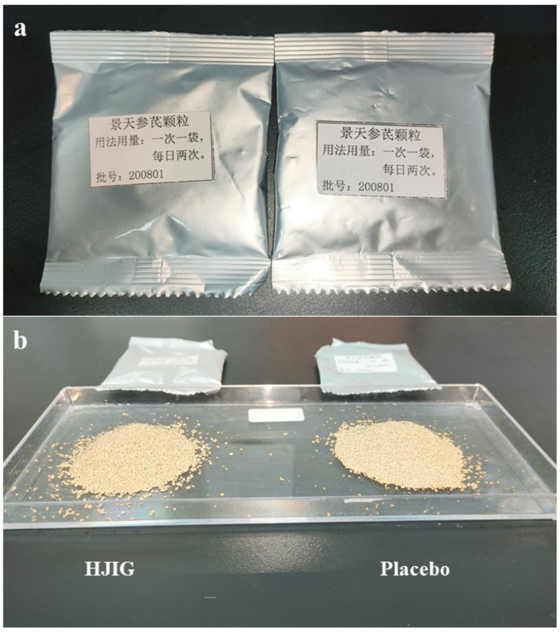

Figure 1 HJIG and Placebo

红景天 (Hong-Jing-Tian) - Rhodiola crenulata (Hook. f. et Thoms.) H. Ohba [Crassulaceae; Rhodiola crenulata (Hook. f. et Thomas.) H. Ohba]

https://mpns.science.kew.org/mpns-portal/plantDetail?plantId=2416737&query=Rhodiola+crenulata+%28Hook.f.+%26+Thomson%29&filter=&fuzzy=false&nameType=all&dbs=wcsCmp

黄芪 (Huang-Qi) - Astragalus mongholicus Bunge [Fabaceae; astragali radix praeparata cum melle]

https://mpns.science.kew.org/mpns-portal/plantDetail?plantId=2661222&query=Astragalus+mongholicus+Bunge&filter=&fuzzy=false&nameType=all&dbs=wcs

党参 (Dang-shen) - Codonopsis pilosula (Franch.) Nannf. [Campanulaceae; Codonopsis pilosula (Franch.) Nannf.]

https://mpns.science.kew.org/mpns-portal/plantDetail?plantId=367867&query=Codonopsis+pilosula+Nannf&filter=&fuzzy=false&nameType=all&dbs=wcs

全当归 (Quan-Dang-Gui) – Angelica sinensis (Oliv.) Diels [Apiaceae; Angelica sinensis (Oliv.) Diels]
https://mpns.science.kew.org/mpns-portal/plantDetail?plantId=2639272&query=Angelicae+sinensis+radix&filter=&fuzzy=false&nameType=all&dbs=wcsCmp

丹参 (Dan-shen) - Salvia miltiorrhiza Bunge [Lamiaceae; Salvia miltiorrhiza Bge.]
https://mpns.science.kew.org/mpns-portal/plantDetail?plantId=183206&query=Salvia+miltiorrhiza+Bunge&filter=&fuzzy=false&nameType=all&dbs=wcs

白芍 (Bai-Shao) - Paeonia lactiflora Pall. [Paeoniaceae; Paeonia lactiflora Pall.]
https://mpns.science.kew.org/mpns-portal/plantDetail?plantId=519125&query=Paeonia+lactiflora+Pall.&filter=&fuzzy=false&nameType=all&dbs=wcs

枸杞 (Gou-Qi) - Lycium chinense Mill. [Solanaceae; Lycium chinense Mill.]
https://mpns.science.kew.org/mpns-portal/plantDetail?plantId=2496344&query=Lycium+chinense+Mill.&filter=&fuzzy=false&nameType=all&dbs=wcsCmp

淫羊藿 (Yin-Yang-Huo) - Epimedium brevicornu Maxim. [Berberidaceae; Epimedium brevicornu Maxim.]

https://mpns.science.kew.org/mpns-portal/plantDetail?plantId=2791222&query=Epimedium+brevicornu+Maxim&filter=&fuzzy=false&nameType=all&dbs=wcsCmp

川牛膝 (Chuan-Niu-Xi) - Cyathula officinalis K.C.Kuan [Amaranthaceae; Cyathula officinalis Kuan]

https://mpns.science.kew.org/mpns-portal/plantDetail?plantId=2749224&query=Cyathula+officinalis&filter=&fuzzy=false&nameType=all&dbs=wcsCmp

检 验 报 告

TEST REPORT

项目名称：景天参芪颗粒

委托单位：浙江中医药大学附属第二医院

报告日期：2020 年 09 月 30 日

浙江佐力药业股份有限公司

检 验 报 告

对景天参芪颗粒所用原辅包材及中间体、成品进行检验，结果如下：

样品名称	质量标准	检验结果
红景天	《中国药典》2015 年版一部	符合规定
白芍	《中国药典》2015 年版一部	符合规定
丹参	《中国药典》2015 年版一部	符合规定
川牛膝	《浙江省中药炮制规范》2015 年版	符合规定
当归	《中国药典》2015 年版一部	符合规定
枸杞子	《中国药典》2015 年版一部	符合规定
炒党参	《浙江省中药炮制规范》2015 年版	符合规定
炙黄芪	《中国药典》2015 年版一部	符合规定
淫羊藿	《浙江省中药炮制规范》2015 年版	符合规定
复合膜	QS-C-001	符合规定
糊精	QS-B-002	符合规定
景天参芪颗粒中间体（干膏粉）	景天参芪颗粒中间体（干膏粉）标准草案	符合规定
景天参芪颗粒中间体（颗粒）	景天参芪颗粒中间体（颗粒）标准草案	符合规定
景天参芪颗粒	景天参芪颗粒标准草案	符合规定

附件：各样品检验报告书

浙江佐力药业股份有限公司

Zhejiang Jolly Pharmaceutical Co., Ltd.

物料检验报告书

物料名称	红景天		物料编号	R470-20071001
供应商	浙江佐力百草中药饮片有限公司		规格	片
产地	西藏		数　量	18.00Kg
供应商生产批号	20200301		检验依据	《中国药典》2015年版一部
报告日期	2020年07月24日		备注	/

检验项目		标准规定	检验结果
性状		应符合规定	符合规定
鉴别	显微鉴别	应具有红景天的显微特征	符合规定
	薄层鉴别	应符合规定	符合规定
检查	水分	应不得过12.0%	5.4%
	总灰分	应不得过8.0%	4.5%
	酸不溶性灰分	应不得过2.0%	0.04%
	二氧化硫残留量	应不得过150 mg/kg	2 mg/kg
浸出物		应不得少于22.0%	48.2%
含量测定		按干燥品计算，含红景天苷（$C_{14}H_{20}O_{7}$）不得少于0.50%。	0.96%

结论：本品按《中国药典》2015年版一部检验，结果符合规定。

审核者	签发者
王丹颖	朱含剑

浙江佐力药业股份有限公司

Zhejiang Jolly Pharmaceutical Co., Ltd.

物料检验报告书

物料名称	白芍	物料编号	R397-20071001
供应商	浙江佐力百草中药饮片有限公司	规格	片
产地	安徽	数 量	48.00Kg
供应商生产批号	20200401	检验依据	《浙江省中药炮制规范》2015年版
报告日期	2020年07月24日	备注	/

检验项目		标准规定	检验结果
性状		应符合规定	符合规定
鉴别	显微鉴别	应具有白芍的显微特征	符合规定
	薄层鉴别	应符合规定	符合规定
检查	水分	应不得过14.0%	10.3%
	总灰分	应不得过4%	2.8%
	二氧化硫残留量	应不得过400 mg/kg	228 mg/kg
浸出物		应不得少于22.0%	28.0%
含量测定		按干燥品计算，含芍药苷（$C_{23}H_{28}O_{11}$）不得少于1.2%。	2.5%

结论：本品按《浙江省中药炮制规范》2015年版检验，结果符合规定。

审核者	签发者
王丹颖	朱[illegible]

浙江佐力药业股份有限公司 3305210033[illegible]

浙 江 佐 力 药 业 股 份 有 限 公 司

Zhejiang Jolly Pharmaceutical Co., Ltd.

物 料 检 验 报 告 书

<table>
<tr><td>物料名称</td><td colspan="2">丹参</td><td>物料编号</td><td colspan="2">R022-20071001</td></tr>
<tr><td>供应商</td><td colspan="3">浙江佐力百草中药饮片有限公司</td><td>规格</td><td>片</td></tr>
<tr><td>产地</td><td colspan="3">山东</td><td>数　量</td><td>48.00Kg</td></tr>
<tr><td colspan="2">供应商生产批号</td><td>20200401</td><td>检验依据</td><td colspan="2">《中国药典》2015 年版一部</td></tr>
<tr><td colspan="2">报告日期</td><td>2020 年 07 月 24 日</td><td>备注</td><td colspan="2">/</td></tr>
</table>

<table>
<tr><td colspan="2">检验项目</td><td>标准规定</td><td>检验结果</td></tr>
<tr><td colspan="2">性状</td><td>应符合规定</td><td>符合规定</td></tr>
<tr><td rowspan="2">鉴别</td><td>显微鉴别</td><td>应具有丹参的显微特征</td><td>符合规定</td></tr>
<tr><td>薄层鉴别</td><td>应符合规定</td><td>符合规定</td></tr>
<tr><td rowspan="4">检查</td><td>水分</td><td>应不得过 13.0%</td><td>9.9%</td></tr>
<tr><td>总灰分</td><td>应不得过 10.0%</td><td>4.7%</td></tr>
<tr><td>酸不溶性灰分</td><td>应不得过 2.0%</td><td>0.6%</td></tr>
<tr><td>二氧化硫残留量</td><td>应不得过 150 mg/kg</td><td>2 mg/kg</td></tr>
<tr><td rowspan="2">浸出物</td><td>水溶性浸出物</td><td>应不得少于 35.0%</td><td>65.5%</td></tr>
<tr><td>醇溶性浸出物</td><td>应不得少于 11.0%。</td><td>17.1%</td></tr>
<tr><td colspan="4">结论：本品按《中国药典》2015 年版一部检验，结果符合规定。</td></tr>
<tr><td colspan="2">审核者</td><td colspan="2">签发者</td></tr>
<tr><td colspan="2"></td><td colspan="2"></td></tr>
</table>

浙江佐力药业股份有限公司

Zhejiang Jolly Pharmaceutical Co., Ltd.

物料检验报告书

物料名称	川牛膝	物料编号	R040-20071001
供应商	浙江佐力百草中药饮片有限公司	规格	片
产地	四川	数量	30.00Kg
供应商生产批号	20200201	检验依据	《浙江省中药炮制规范》2015年版
报告日期	2020年07月24日	备注	/

检验项目		标准规定	检验结果
性状		应符合规定	符合规定
鉴别	显微鉴别	应具有川牛膝的显微特征	符合规定
	薄层鉴别	应符合规定	符合规定
检查	水分	应不得过12.0%	6.0%
	总灰分	应不得过8.0%	4.6%
	二氧化硫残留量	应不得过150 mg/kg	0 mg/kg
浸出物		应不得少于60.0%	71.2%
含量测定		按干燥品计算，含杯苋甾酮（$C_{29}H_{44}O_8$）不得少于0.030%。	0.112%

结论：本品按《浙江省中药炮制规范》2015年版检验，结果符合规定。

审核者	签发者
王卉颖	朱为钊

浙江佐力药业股份有限公司

Zhejiang Jolly Pharmaceutical Co., Ltd.

物料检验报告书

物料名称	当归	物料编号	R273-20071001
供应商	浙江佐力百草中药饮片有限公司	规格	片
产地	甘肃	数量	36.00Kg
供应商生产批号	20200301	检验依据	《中国药典》2015 年版一部
报告日期	2020 年 07 月 24 日	备注	/

检验项目		标准规定	检验结果
性状		应符合规定	符合规定
鉴别	显微鉴别	应具有当归的显微特征	符合规定
	薄层鉴别（1）	应符合规定	符合规定
	薄层鉴别（2）	应符合规定	符合规定
检查	水分	应不得过 15.0%	9.8%
	总灰分	应不得过 7.0%	4.7%
	酸不溶性灰分	应不得过 2.0%	0.6%
	二氧化硫残留量	应不得过 150 mg/kg	0 mg/kg
浸出物		应不得少于 45.0%	63.7%

结论：本品按《中国药典》2015 年版一部检验，结果符合规定。

审核者	签发者
王丹颖	朱方剑

浙江佐力药业股份有限公司

Zhejiang Jolly Pharmaceutical Co., Ltd.

物料检验报告书

物料名称	枸杞子	物料编号	R025-20071001
供应商	浙江佐力百草中药饮片有限公司	规格	片
产地	宁夏	数　量	36.00Kg
供应商生产批号	20200401	检验依据	《中国药典》2015年版一部
报告日期	2020年07月24日	备注	/

检验项目		标准规定	检验结果
性状		应符合规定	符合规定
鉴别	显微鉴别	应具有枸杞子的显微特征	符合规定
鉴别	薄层鉴别	应符合规定	符合规定
检查	水分	应不得过13.0%	7.3%
检查	总灰分	应不得过5.0%	4.1%
检查	二氧化硫残留量	应不得过150 mg/kg	19 mg/kg
检查	重金属及有害元素	铅不得过5 mg/kg	小于5 mg/kg
检查	重金属及有害元素	镉不得过0.3 mg/kg	小于0.3 mg/kg
检查	重金属及有害元素	砷不得过2 mg/kg	小于2 mg/kg
检查	重金属及有害元素	汞不得过0.2 mg/kg	小于0.2 mg/kg
检查	重金属及有害元素	铜不得过20 mg/kg	小于20 mg/kg
浸出物		应不得少于55.0%	78.5%
含量测定	枸杞多糖以葡萄糖	按干燥品计算，含枸杞多糖以葡萄糖（$C_6H_{12}O_6$）不得少于1.8%	3.4%
含量测定	甜菜碱	按干燥品计算，含甜菜碱（$C_5H_{11}NO_2$）不得少于0.30%。	0.76%

结论：本品按《中国药典》2015年版一部检验，结果符合规定。

审核者	签发者
王[illegible]颖	朱方剑

浙 江 佐 力 药 业 股 份 有 限 公 司

Zhejiang Jolly Pharmaceutical Co., Ltd.

物 料 检 验 报 告 书

物料名称	炒党参	物料编号	R618-20071001
供应商	浙江佐力百草中药饮片有限公司	规格	炒
产地	甘肃	数 量	48.00Kg
供应商生产批号	20200602	检验依据	《浙江省中药炮制规范》2015 年版
报告日期	2020 年 07 月 24 日	备注	/

检验项目		标准规定	检验结果
性状		应符合规定	符合规定
薄层鉴别		应符合规定	符合规定
检查	水分	应不得过 10.0%	9.1%
	总灰分	应不得过 5.0%	4.1%
	二氧化硫残留量	应不得过 400 mg/kg	29 mg/kg
浸出物		应不得少于 55.0%	66.3%

结论：本品按《浙江省中药炮制规范》2015 年版检验，结果符合规定。

审核者	签发者
王舟颖	朱尔钊

浙江佐力药业股份有限公司 3305210033616

浙江佐力药业股份有限公司

Zhejiang Jolly Pharmaceutical Co., Ltd.

物料检验报告书

物料名称	炙黄芪	物料编号	R171-20071001
供应商	浙江佐力百草中药饮片有限公司	规格	蜜炙
产地	甘肃	数　量	30.00Kg
供应商生产批号	20200201	检验依据	《中国药典》2015 年版一部
报告日期	2020 年 07 月 24 日	备注	/

检验项目		标准规定	检验结果
性状		应符合规定	符合规定
鉴别	薄层鉴别（1）	应符合规定	符合规定
	薄层鉴别（2）	应符合规定	符合规定
检查	水分	应不得过 10.0%	9.1%
	总灰分	应不得过 4.0%	3.0%
	二氧化硫残留量	应不得过 150 mg/kg	0 mg/kg
含量测定	黄芪甲苷	按干燥品计算，含黄芪甲苷（$C_{41}H_{68}O_{14}$）应不得少于 0.030%	0.061%
	毛蕊异黄酮葡萄糖苷	按干燥品计算，含毛蕊异黄酮葡萄糖苷（$C_{22}H_{22}O_{10}$）应不得少于 0.020%。	0.056%
结论：本品按《中国药典》2015 年版一部检验，结果符合规定。			

审核者	签发者
王丹颖	朱方剑

浙江佐力药业股份有限公司

Zhejiang Jolly Pharmaceutical Co., Ltd.

物料检验报告书

物料名称	淫羊藿	物料编号	R055-16102801	
供应商	浙江佐力百草中药饮片有限公司		规格	片
产地	陕西		数量	30.00Kg
供应商生产批号	20200101	检验依据	《浙江省中药炮制规范》2015年版	
报告日期	2020年07月24日	备注	/	

检验项目		标准规定	检验结果
性状		应符合规定	符合规定
薄层鉴别		应符合规定	符合规定
检查	总灰分	应不得过8.0%	6.4%
	二氧化硫残留量	应不得过150 mg/kg	3 mg/kg
含量测定	淫羊藿苷	按干燥品计算，含淫羊藿苷（$C_{33}H_{40}O_{15}$）不得少于0.40%	0.56%
	总黄酮	按干燥品计算，含总黄酮以淫羊藿苷（$C_{33}H_{40}O_{15}$）不得少于5.0%。	10.9%

结论：本品按《浙江省中药炮制规范》2015年版检验，结果符合规定。

审核者	签发者
肖颖	朱万金

浙江佐力药业股份有限公司

Zhejiang Jolly Pharmaceutical Co., Ltd.

物料检验报告书

物料名称	复合膜	物料编号	D078-19110101
供应商	杭州中盈医药包装印刷有限公司	规格	0.08×120mm
检验依据	QS-C-001	数　量	30.00Kg
供应商生产批号	20191015C	报告日期	2020年07月28日

检验项目		标准规定	检验结果
外观		不得有穿孔、异物、异味、粘连，复合层间分离及明显损伤、气泡、皱纹、脏污等缺陷。	符合规定
尺寸		厚度 0.08±0.006mm；宽度 120±0.5mm	符合规定
溶出物试验	易氧化物	供试液与空白液消耗硫代硫酸钠滴定液（0.01mol/L）之差不得过 1.5ml	1.2ml
	不挥发物	水不挥发物残渣与其空白残渣之差应不得过 30.0mg;	4.9mg
		65%乙醇不挥发物残渣与其空白残渣之差应不得过 30.0mg;	1.3mg
		正己烷不挥发物残渣与其空白残渣之差应不得过 30.0mg;	20.2mg
	重金属	不得过百万分之一	符合规定
微生物检查	需氧菌总数	不得过 500cfu/100cm²	小于 30cfu/100cm²
	霉菌和酵母菌总数	不得过 50cfu/100cm²	小于 30cfu/100cm²
	大肠埃希菌	不得检出	未检出

结论：本品按 QS-C-001 检验，结果符合规定。

审核者	签发者
姚[illegible]（签名）	

浙江佐力药业股份有限公司 3305210033816

浙江佐力药业股份有限公司

Zhejiang Jolly Pharmaceutical Co., Ltd.

物料检验报告书

物料名称	糊 精		物料编号	B003-20072201	
供应商	嘉兴市白浪淀粉制品有限公司			规格	25kg/袋
数 量	200.00kg	供应商生产批号	2020070602	检验依据	QS-B-002
报告日期	2020 年 07 月 30 日		备注	/	

检验项目			标准规定	检验结果
性 状			本品为白色或类白色的无定形粉末；无臭；味微甜	符合规定
鉴 别			应显紫红色	符合规定
检查	干燥失重		减失重量不得过 10.0%	3.5%
	酸度		应显粉红色	符合规定
	还原糖		遗留的氧化亚铜不得过 0.20g	0.18g
	炽灼残渣		不得过 0.5%	0.1%
	重金属		不得过百万分之二十	符合规定
	铁盐		不得更深（0.005%）	符合规定
	微生物限度	需氧菌总数	不得过 500cfu/g	小于 10cfu/g
		霉菌、酵母菌总数	不得过 50cfu/g	小于 10cfu/g
		控制菌	不得检出大肠埃希菌（1g）	未检出

结论：本品按 QS-B-002 检验，结果符合规定。

审核者	签发者
[illegible]	[illegible]

浙江佐力药业股份有限公司

Zhejiang Jolly Pharmaceutical Co., Ltd.

中间体检验报告书

产品名称	景天参芪颗粒中间体（干膏粉）	产品批号	200801	规　　格	/
生产日期	2020年08月	取样量	50g	报告日期	2020年08月10日
厂家	浙江佐力药业股份有限公司				
检验依据	景天参芪颗粒中间体（干膏粉）标准草案				

检验项目	标准规定	检验结果
性　状	本品为黄色至棕黄色的粉末；气微，味苦。	符合规定
水分	不得过5.0%	3.8%
结论：本品按景天参芪颗粒中间体（干膏粉）标准草案检验，结果符合规定		

审核者	签发者
李[illegible]	朱[illegible]

浙江佐力药业股份有限公司

Zhejiang Jolly Pharmaceutical Co., Ltd.

中间体检验报告书

产品名称	景天参芪颗粒中间体（颗粒）	产品批号	200801	规　　格	/
生产日期	2020年08月	取样量	50g	报告日期	2020年08月18日
厂家	浙江佐力药业股份有限公司				
检验依据	景天参芪颗粒中间体（颗粒）标准草案				

检验项目	标准规定	检验结果
性　状	本品为黄色至棕黄色的颗粒；气微，味苦。	符合规定
水分	不得过6.0%	4.5%
粒度	不能通过一号筛与能通过五号筛的总和不得过15%	3%
溶化性	本品应全部溶化或轻微浑浊	符合规定

结论：本品按景天参芪颗粒中间体（颗粒）标准草案检验，结果符合规定

审核者	签发者
[illegible]	[illegible]

浙江佐力药业股份有限公司

Zhejiang Jolly Pharmaceutical Co., Ltd.

物料检验报告书

产品名称	景天参芪颗粒	产品批号	200801	规　　格	每袋装 8.5 克
生产日期	2020 年 08 月	有效期至	2023 年 07 月	报告日期	2020 年 09 月 25 日
厂家	浙江佐力药业股份有限公司				
检验依据	景天参芪颗粒标准草案				

检验项目		标准规定	检验结果
性　状		本品为黄色至棕黄色的颗粒；气微，味苦。	符合规定
鉴别	鉴别 1	供试品色谱中应分别呈现与对照品色谱峰保留时间相应的色谱峰。	符合规定
	鉴别 2	在与对照品色谱相应的位置上，显相同颜色的斑点	符合规定
	鉴别 3	在与对照品色谱相应的位置上，显相同颜色的斑点	符合规定
水分		不得过 8.0%	4.7%
粒度		不能通过一号筛与能通过五号筛的总和不得过 15%	3%
溶化性		本品应全部溶化或轻微浑浊	符合规定
装量差异		装量差异限度±10%	符合规定
微生物限度	需氧菌总数	不得过 500cfu/100cm2	小于 $80cfu/100cm^2$
	霉菌和酵母菌总数	不得过 50cfu/100cm2	小于 $20cfu/100cm^2$
	大肠埃希菌	不得检出	未检出
含量测定		本品每袋含红景天苷($C_{14}H_{20}O_7$)不得少于 10mg。	18.2mg/袋

结论：本品按景天参芪颗粒标准草案检验，结果符合规定

审核者	签发者
李[illegible]	朱[illegible]

TEST REPORT

Project Name: **Hongjing I Granule (HJIG)**

Client : **Zhejiang Chinese Medical University Affiliated Second Hospital**

Report Date : **September 30, 2020**

Zhejiang Jolly Pharmaceutical Co., Ltd.

Seal: Zhejiang Jolly Pharmaceutical Co., Ltd.

Test Report

The raw materials, intermediates, and finished products of Jingtian Sanye Granule were inspected with the following results:

Sample Name	Quality Standard	Test Results
Rhodiola crenulata (Hook. f. et Thoms.) H. Ohba	Chinese Pharmacopoeia 2015 Edition, Part I	Conforms to standard
Paeonia lactiflora Pall.	Chinese Pharmacopoeia 2015 Edition, Part I	Conforms to standard
Salvia miltiorrhiza Bunge	Chinese Pharmacopoeia 2015 Edition, Part I	Conforms to standard
Cyathula officinalis K.C.Kuan	Zhejiang Provincial Chinese Medicine Processing Standard 2015 Edition	Conforms to standard
Angelica sinensis (Oliv.) Diels	Chinese Pharmacopoeia 2015 Edition, Part I	Conforms to standard
Lycium chinense Mill.	Chinese Pharmacopoeia 2015 Edition, Part I	Conforms to standard
Codonopsis pilosula (Franch.) Nannf.	Zhejiang Provincial Chinese Medicine Processing Standard 2015 Edition	Conforms to standard
Astragalus mongholicus Bunge	Chinese Pharmacopoeia 2015 Edition, Part I	Conforms to standard
Epimedium brevicornu Maxim.	Zhejiang Provincial Chinese Medicine Processing Standard 2015 Edition	Conforms to standard
Composite Membrane	QS-C-001	Conforms to standard
Maltodextrin	QS-B-002	Conforms to standard
HJIG Intermediate (Dried Powder)	HJIG Intermediate (Dried Powder) Standard	Conforms to standard
HJIG Intermediate (Granule)	HJIG Intermediate (Granule) Standard	Conforms to standard
HJIG	HJIG Standard	Conforms to standard

Appendix: Sample Test Reports

Zhejiang Jolly Pharmaceutical Co., Ltd.

Material Test Report

**Material Name**	Rhodiola crenulata (Hook. f. et Thoms.) H. Ohba
**Material Code**	R470-20071001
**Supplier**	Zhejiang Jolly Pharmaceutical Co., Ltd.
**Specification**	Sheet
**Place of Origin**	Tibet
**Quantity**	18.00Kg
**Supplier Batch Number**	20200301
**Standard**	Chinese Pharmacopoeia 2015 Edition, Part I
**Report Date**	July 24, 2020
**Notes**	/

**Test Item**		**Standard Specification**	**Test Results**
Appearance		Conforms to the standard	Conforms to the standard
Identification	Microscopic Identification	Must show the distinctive features of Rhodiola Rosea	Conforms to the standard
	TLC Identification	Conforms to the standard	Conforms to the standard
Inspection	Moisture	Must not exceed 12.0%	5.4%
	Total Ash	Must not exceed 8.0%	4.5%
	Acid-insoluble Ash	Must not exceed 2.0%	0.04%
	Sulfur Dioxide Residue	Must not exceed 150 mg/kg	2 mg/kg
Extractives		Must not be less than 22.0%	48.2%
Assay		Calculated on the dried basis, must contain not less than 0.50% of salidroside ($C_{14}H_{20}O_7$)	0.96%

Conclusion: This product complies with the standards of the Chinese Pharmacopoeia 2015 Edition, Part I.

Reviewer: Wang Shenying

Approver: Zhu Fangjian

Zhejiang Jolly Pharmaceutical Co., Ltd.

Material Test Report

**Material Name**	Paeonia lactiflora Pall.
**Material Code**	R397-20071001
**Supplier**	Zhejiang Jolly Pharmaceutical Co., Ltd.
**Specification**	Sheet
**Place of Origin**	Anhui
**Quantity**	48.00Kg
**Supplier Batch Number**	20200401
**Standard**	Zhejiang Provincial Standard for Chinese Medicine Processing 2015 Edition
**Report Date**	July 24, 2020
**Notes**	/

**Test Item**		**Standard Specification**	**Test Results**
Appearance		Conforms to the standard	Conforms to the standard
Identification	Microscopic Identification	Must show the distinctive features of White Peony Root	Conforms to the standard
	TLC Identification	Conforms to the standard	Conforms to the standard
Inspection	Moisture	Must not exceed 14.0%	10.3%
	Total Ash	Must not exceed 4%	2.8%
	Sulfur Dioxide Residue	Must not exceed 400 mg/kg	228 mg/kg
Extractives		Must not be less than 22.0%	28.0%
Assay		Calculated on the dried basis, must contain not less than 1.2% of paeoniflorin ($C_{23}H_{28}O_{11}$)	2.5%

Conclusion: This product complies with the standards of Zhejiang Provincial Standard for Chinese Medicine Processing 2015 Edition.

Reviewer: Wang Shenying

Approver: Zhu Fangjian

Zhejiang Jolly Pharmaceutical Co., Ltd.

Material Test Report

**Material Name**	Salvia miltiorrhiza Bunge
**Material Code**	R022-20071001
**Supplier**	Zhejiang Jolly Pharmaceutical Co., Ltd.
**Specification**	Sheet
**Place of Origin**	Shandong
**Quantity**	48.00Kg
**Supplier Batch Number**	20200401
**Standard**	Chinese Pharmacopoeia 2015 Edition, Part I
**Report Date**	July 24, 2020
**Notes**	/

**Test Item**		**Standard Specification**	**Test Results**
Appearance		Conforms to the standard	Conforms to the standard
Identification	Microscopic Identification	Must show the distinctive features of Danshen Root	Conforms to the standard
	TLC Identification	Conforms to the standard	Conforms to the standard
Inspection	Moisture	Must not exceed 13.0%	9.9%
	Total Ash	Must not exceed 10.0%	4.7%
	Acid-insoluble Ash	Must not exceed 2.0%	0.6%
	Sulfur Dioxide Residue	Must not exceed 150 mg/kg	2 mg/kg
Extractives	Water-soluble Extractives	Must not be less than 35.0%	65.5%
	Alcohol-soluble Extractives	Must not be less than 11.0%	17.1%

Conclusion: This product complies with the standards of Chinese Pharmacopoeia 2015 Edition, Part I.

Reviewer: Wang Shenying

Approver: Zhu Fangjian

Zhejiang Jolly Pharmaceutical Co., Ltd.

Material Test Report

**Material Name**	Cyathula officinalis K.C.Kuan
**Material Code**	R040-20071001
**Supplier**	Zhejiang Jolly Pharmaceutical Co., Ltd.
**Specification**	Sheet
**Place of Origin**	Sichuan
**Quantity**	30.00Kg
**Supplier Batch Number**	20200201
**Standard**	Zhejiang Provincial Chinese Medicine Preparation Standards 2015 Edition
**Report Date**	July 24, 2020
**Notes**	/

**Test Item**		**Standard Specification**	**Test Results**
Appearance		Conforms to the standard	Conforms to the standard
Identification	Microscopic Identification	Must show the distinctive features of Chuanniuxi Root	Conforms to the standard
	TLC Identification	Conforms to the standard	Conforms to the standard
Inspection	Moisture	Must not exceed 12.0%	6.0%
	Total Ash	Must not exceed 8.0%	4.6%
	Sulfur Dioxide Residue	Must not exceed 150 mg/kg	0 mg/kg
Extractives		Must not be less than 60.0%	71.2%
Content Determination		Calculated as β-ecdysterone ($C_{29}H_{44}O_8$), must not be less than 0.030%	0.112%

Conclusion: This product complies with the standards of Zhejiang Provincial Chinese Medicine Preparation Standards 2015 Edition.

Reviewer: Wang Shenying

Approver: Zhu Fangjian

Zhejiang Jolly Pharmaceutical Co., Ltd.

Material Test Report

**Material Name**	Angelica sinensis (Oliv.) Diels
**Material Code**	R273-20071001
**Supplier**	Zhejiang Jolly Pharmaceutical Co., Ltd.
**Specification**	Sheet
**Place of Origin**	Gansu
**Quantity**	36.00Kg
**Supplier Batch Number**	20200301
**Standard**	Chinese Pharmacopoeia 2015 Edition Part I
**Report Date**	July 24, 2020
**Notes**	/

**Test Item**		**Standard Specification**	**Test Results**
Appearance		Conforms to the standard	Conforms to the standard
Identification	Microscopic Identification	Must show the distinctive features of Danggui	Conforms to the standard
	TLC Identification (1)	Conforms to the standard	Conforms to the standard
	TLC Identification (2)	Conforms to the standard	Conforms to the standard
Inspection	Moisture	Must not exceed 15.0%	9.8%
	Total Ash	Must not exceed 7.0%	4.7%
	Acid-Insoluble Ash	Must not exceed 2.0%	0.6%
	Sulfur Dioxide Residue	Must not exceed 150 mg/kg	0 mg/kg
Extractives		Must not be less than 45.0%	63.7%

Conclusion: This product complies with the standards of the Chinese Pharmacopoeia 2015 Edition Part I.

Reviewer: Wang Shenying

Approver: Zhu Fangjian

Zhejiang Jolly Pharmaceutical Co., Ltd.

Material Test Report

Material Name	Lycium chinense Mill.
**Material Code**	R025-20071001
**Supplier**	Zhejiang Jolly Pharmaceutical Co., Ltd.
**Specification**	Sheet
**Place of Origin**	Ningxia
**Quantity**	36.00Kg
**Supplier Batch Number**	20200401
**Standard**	Chinese Pharmacopoeia 2015 Edition Part I
**Report Date**	July 24, 2020
**Notes**	/

Test Item		Standard Specification	Test Results
Appearance		Conforms to the standard	Conforms to the standard
Identification	Microscopic Identification	Must show the distinctive features of Gouqi	Conforms to the standard
	TLC Identification	Conforms to the standard	Conforms to the standard
Inspection	Moisture	Must not exceed 13.0%	7.3%
	Total Ash	Must not exceed 5.0%	4.1%
	Sulfur Dioxide Residue	Must not exceed 150 mg/kg	19 mg/kg
	Heavy Metals and Harmful Elements	Lead (Pb) must not exceed 5 mg/kg	< 5 mg/kg
		Cadmium (Cd) must not exceed 0.3 mg/kg	< 0.3 mg/kg
		Arsenic (As) must not exceed 2 mg/kg	< 2 mg/kg
		Mercury (Hg) must not exceed 0.2 mg/kg	< 0.2 mg/kg
		Copper (Cu) must not exceed 20 mg/kg	< 20 mg/kg
Extractives		Must not be less than 55.0%	78.5%
Content Determination	Gouqi Polysaccharides	Must not be less than 1.8%	3.4%
	Betanin	Must not be less than 0.30%	0.76%

Conclusion: This product complies with the standards of the Chinese Pharmacopoeia 2015 Edition Part I.

Reviewer: Wang Shenying

Approver: Zhu Fangjian

Zhejiang Jolly Pharmaceutical Co., Ltd.

Material Test Report

**Material Name**	Codonopsis pilosula (Franch.) Nannf.
**Material Code**	R618-20071001
**Supplier**	Zhejiang Jolly Pharmaceutical Co., Ltd.
**Specification**	Roasted
**Place of Origin**	Gansu
**Quantity**	48.00Kg
**Supplier Batch Number**	20200602
**Standard**	Zhejiang Provincial Standards for Processed Chinese Herbal Medicine 2015 Edition
**Report Date**	July 24, 2020
**Notes**	/

**Test Item**		**Standard Specification**	**Test Results**
Appearance		Conforms to the standard	Conforms to the standard
TLC Identification		Conforms to the standard	Conforms to the standard
Inspection	Moisture	Must not exceed 10.0%	9.1%
	Total Ash	Must not exceed 5.0%	4.1%
	Sulfur Dioxide Residue	Must not exceed 400 mg/kg	29 mg/kg
Extractives		Must not be less than 55.0%	66.3%

Conclusion: This product complies with the standards of the Zhejiang Provincial Standards for Processed Chinese Herbal Medicine 2015 Edition.

Reviewer: Wang Shenying

Approver: Zhu Fangjian

Zhejiang Jolly Pharmaceutical Co., Ltd.

Material Test Report

**Material Name**	Astragalus mongholicus Bunge
**Material Code**	R171-20071001
**Supplier**	Zhejiang Jolly Pharmaceutical Co., Ltd.
**Specification**	Honey-baked
**Place of Origin**	Gansu
**Quantity**	30.00Kg
**Supplier Batch Number**	20200201
**Standard**	Chinese Pharmacopoeia 2015 Edition Part I
**Report Date**	July 24, 2020
**Notes**	/

**Test Item**		**Standard Specification**	**Test Results**
Appearance		Conforms to the standard	Conforms to the standard
Identification	TLC Identification (1)	Conforms to the standard	Conforms to the standard
	TLC Identification (2)	Conforms to the standard	Conforms to the standard
Inspection	Moisture	Must not exceed 10.0%	9.1%
	Total Ash	Must not exceed 4.0%	3.0%
	Sulfur Dioxide Residue	Must not exceed 150 mg/kg	0 mg/kg
Content Determination	Huangqi Methoxyflavone	Not less than 0.030%	0.061%
	Hedysarum Mongolicum Polysaccharide	Not less than 0.020%	0.056%

Conclusion: This product complies with the standards of the Chinese Pharmacopoeia 2015 Edition Part I.

Reviewer: Wang Shenying

Approver: Zhu Fangjian

Zhejiang Jolly Pharmaceutical Co., Ltd.

Material Test Report

**Material Name**	Epimedium brevicornu Maxim.
**Material Code**	R055-16102801
**Supplier**	Zhejiang Jolly Pharmaceutical Co., Ltd.
**Specification**	Sheet
**Place of Origin**	Shaanxi
**Quantity**	30.00Kg
**Supplier Batch Number**	20200101
**Standard**	Zhejiang Provincial Chinese Medicine Paozhi Standards 2015 Edition
**Report Date**	July 24, 2020
**Notes**	/

<table>
<tr><th colspan="2">Test Item</th><th>Standard Specification</th><th>Test Results</th></tr>
<tr><td colspan="2">Appearance</td><td>Conforms to the standard</td><td>Conforms to the standard</td></tr>
<tr><td colspan="2">TLC Identification</td><td>Conforms to the standard</td><td>Conforms to the standard</td></tr>
<tr><td rowspan="2">Inspection</td><td>Total Ash</td><td>Must not exceed 8.0%</td><td>6.4%</td></tr>
<tr><td>Sulfur Dioxide Residue</td><td>Must not exceed 150 mg/kg</td><td>3 mg/kg</td></tr>
<tr><td rowspan="2">Content Determination</td><td>Icariin</td><td>Not less than 0.40%</td><td>0.56%</td></tr>
<tr><td>Total Flavonoids</td><td>Not less than 5.0%</td><td>10.9%</td></tr>
</table>

Conclusion: This product complies with the standards of the Zhejiang Provincial Chinese Medicine Paozhi Standards 2015 Edition.

Reviewer: Xiao liu

Approver: Zhu Fangjian

Zhejiang Jolly Pharmaceutical Co., Ltd.

Material Test Report

**Material Name**	Composite Membrane
**Material Code**	D078-19110101
**Supplier**	Hangzhou Zhongying Medical Packaging Printing Co., Ltd.
**Specification**	0.08 × 120mm
**Inspection Standard**	QS-C-001
**Quantity**	30.00Kg
**Supplier Batch Number**	20191015C
**Report Date**	July 28, 2020

**Test Item**		**Standard Specification**	**Test Results**
Appearance		No punctures, foreign matter, dirt, viscosity, peeling of composite layers, and no significant separation or damage, bubbles, sensitivity, or other defects.	Conforms to the standard
Dimensions		Thickness 0.08 ± 0.006mm; Width 120 ± 0.5mm	Conforms to the standard
Dissolution test	Oxidizable Substances	Difference between the test solution and blank titrated with potassium permanganate solution (0.01mol/L) should not exceed 1.5ml	1.2ml
	Non-Volatile Residue Test	Residue from evaporation should not exceed 30.0mg when tested with water	4.9mg
		Residue from evaporation should not exceed 30.0mg when tested with 65% ethanol	1.3mg
		Residue from evaporation should not exceed 30.0mg when tested with n-hexane	20.2mg
	Heavy Metals	Should not exceed 1 ppm	Conforms to the standard
Microbial Limit Test	Aerobic bacterial count	Should not exceed 500cfu/100cm²	< 30cfu/100cm²
	Total yeast and mold count	Should not exceed 50cfu/100cm²	< 30cfu/100cm²
	Escherichia coli	Not detectable	Not detected

Conclusion: This product complies with the QS-C-001 standard.

Reviewer: Yao Teng

Approver: Zhu Fangjian

Zhejiang Jolly Pharmaceutical Co., Ltd.

Material Inspection Report

**Material Name**	Maltodextrin
**Material Code**	B003-20072201
**Supplier**	Jiaxing Tianshun Starch Manufacturing Co., Ltd.
**Specification**	25kg/bag
**Quantity**	200.00kg
**Supplier Batch Number**	2020070602
**Inspection Basis**	QS-B-002
**Report Date**	July 30, 2020
**Remarks**	/

<table>
<tr><th colspan="3">Test Item</th><th>Standard Specification</th><th>Test Result</th></tr>
<tr><td colspan="3">Appearance</td><td>White or off-white amorphous powder; odorless; slightly sweet taste</td><td>Conforms to the standard</td></tr>
<tr><td colspan="3">Identification</td><td>Should show a purple-red color</td><td>Conforms to the standard</td></tr>
<tr><td rowspan="9">Inspection</td><td colspan="2">Dry Weight Loss</td><td>Not more than 10.0%</td><td>3.5%</td></tr>
<tr><td colspan="2">Acidity</td><td>Should show red color</td><td>Conforms to the standard</td></tr>
<tr><td colspan="2">Reducing Sugars</td><td>Not more than 0.20g</td><td>0.18g</td></tr>
<tr><td colspan="2">Sulfate Ash</td><td>Not more than 0.5%</td><td>0.1%</td></tr>
<tr><td colspan="2">Heavy Metals</td><td>Not more than 20ppm</td><td>Conforms to the standard</td></tr>
<tr><td colspan="2">Iron</td><td>Not more than 0.005%</td><td>Conforms to the standard</td></tr>
<tr><td rowspan="3">Microbial limit</td><td>Aerobic Bacteria Count</td><td>Not more than 500cfu/g</td><td>Less than 10cfu/g</td></tr>
<tr><td>Yeast and Mold Count</td><td>Not more than 50cfu/g</td><td>Less than 10cfu/g</td></tr>
<tr><td>Pathogenic Bacteria</td><td>Should not be detected in 1g</td><td>Not detected</td></tr>
</table>

Conclusion: This product conforms to QS-B-002 standards.

Reviewer: Yao Teng

Approver: Zhu Fangjian

Zhejiang Jolly Pharmaceutical Co., Ltd.

Intermediate Inspection Report

**Product Name**	Intermediate of HJIG<br>(Dried Ginger Powder)
**Product Batch Number**	200801
**Specification**	/
**Manufacturing Date**	August 2020
**Sample Quantity**	50g
**Report Date**	August 10, 2020
**Manufacturer**	Zhejiang Jolly Pharmaceutical Co., Ltd.
**Inspection Basis**	Standard Herb Record for Intermediate of HJIG(Dried Ginger Powder)

Test Item	Standard Specification	Test Result
Appearance	This product is yellow to brown-yellow powder; slightly odorous, bitter taste.	Conforms to the standard
Moisture Content	Not more than 5.0%	3.8%

Conclusion: This product complies with the standard herb record for the intermediate of HJIG (Dried Ginger Powder).

Reviewer: Li Zhe

Approver: Zhu Fangjian

Zhejiang Jolly Pharmaceutical Co., Ltd.

Intermediate Inspection Report

**Product Name**	HJIG Intermediate (Granules)
**Production Batch Number**	200801
**Specification**	/
**Production Date**	August 2020
**Sample Quantity**	50g
**Report Date**	August 18, 2020
**Manufacturer**	Zhejiang Jolly Pharmaceutical Co., Ltd.
**Inspection Basis**	HJIG Intermediate (Granules) Standard

**Test Item**	**Standard Specification**	**Test Result**
Appearance	The product is yellow to brown-yellow granules; faint odor, bitter taste	Conforms to the standard
Moisture	Not more than 6.0%	4.5%
Particle Size	No more than 15% passing through No. 1 sieve and retained by No. 5 sieve combined.	3%
Solubility	Should completely dissolve or slightly disperse	Conforms to the standard

Conclusion: This product conforms to the standard of HJIG Intermediate (Granules) upon inspection.

Reviewer: Li Zhe

Approver: Zhu Fangjian

Zhejiang Jolly Pharmaceutical Co., Ltd.

Material Inspection Report

**Product Name**	Jingtian Shenqi Granules
**Product Batch Number**	200801
**Specification**	Each pack 8.5g
**Production Date**	August 2020
**Expiry Date**	July 2023
**Report Date**	September 25, 2020
**Manufacturer**	Zhejiang Jolly Pharmaceutical Co., Ltd.
**Inspection Basis**	Jingtian Shenqi Granules Standard Draft

**Test Items**		**Standard Specification**	**Test Result**
Appearance		This product is yellow to brown-yellow granules; slight odor, bitter taste.	Conforms to specifications
Identification	Identification 1	The position of the peaks in the chromatogram of the test solution corresponds to those of the reference solution.	Conforms to specifications
	Identification 2	The position of the color spots in the chromatogram of the test solution corresponds to those of the reference solution.	Conforms to specifications
	Identification 3	The position of the color spots in the chromatogram of the test solution corresponds to those of the reference solution.	Conforms to specifications
Moisture		Not more than 8.0%	4.7%
Granularity		No more than 15% passing through No. 1 sieve and retained by No. 5 sieve combined.	3%
Solubility		The product should completely dissolve or slightly turbid.	Conforms to specifications
Equipment variance		Equipment difference limit ± 10%	Conforms to specifications
Microbial Limits	Aerobic bacteria count	≤ 500cfu/100cm^2	≤ 80cfu/100cm^2
	Yeast and mold count	≤ 50cfu/100cm^2	≤ 20cfu/100cm^2
	Escherichia	Not detectable	Not detected

	coli		
Content		Each pack contains not less than 10mg of salidroside ($C_{14}H_{20}O_7$)	18.2mg/pack

Conclusion: The product conforms to the Jingtian Shenqi Granules standard draft based on the inspection results.

Reviewer: Li Zhe

Approver: Zhu Fangjian
