# Supplementary material for: Efficacy of Hongjing I granule, an herbal medicine, in patients with mild to moderate erectile dysfunction in a randomized controlled trial
Source: Front Pharmacol. 2024 Dec 24;15:1367812. doi: 10.3389/fphar.2024.1367812 (PMC11703738; doi:10.3389/fphar.2024.1367812)
Supplement: Supplementary file 4 [file DataSheet1.pdf]

International Journal of General Medicine

Dovepress
open access to scientific and medical research

Open Access Full Text Article

STUDY PROTOCOL

Efficacy of Chinese Herbal Medicine Formula in the Treatment of Mild to Moderate Erectile Dysfunction: Study Protocol for a Multi-Center, Randomized, Double-Blinded, Placebo-Controlled Clinical Trial

International Journal of General Medicine downloaded from https://www.dovepress.com/
For personal use only.

Run-Nan Xu[1], Jian-Xiong Ma[2], Xin Zhang[1], Ze-Dong Liao[2], Yi-Jia Fu[1], Bo-Dong Lv[2]

[1]The Second Affiliated Clinical Medical College, Zhejiang Chinese Medical University, Hangzhou, Zhejiang, People's Republic of China; [2]Department of Urology, School of Medicine, The Second Affiliated Hospital, Zhejiang University, Hangzhou, Zhejiang, People's Republic of China

Correspondence: Bo-Dong Lv, Department of Urology, School of Medicine, The Second Affiliated Hospital, Zhejiang University, 88 Jiefang Road, Shangcheng District, Hangzhou City, Zhejiang Province, People's Republic of China, Tel +8613805722233, Email lbd168@zju.edu.cn

Introduction: Erectile dysfunction (ED) is a prevalent condition in urology, primarily managed with PDE5 inhibitors (PDE5Is). However, approximately 20% of patients do not experience improvement in overall sexual satisfaction (OS) after taking PDE5Is. Among these, traditional Chinese medicine (TCM) has emerged as a complementary approach, with formulas like Hongjing I granules (HJIG) showing promise in preliminary studies. This study aims to rigorously evaluate the effectiveness and safety of HJIG in mild to moderate ED cases, assessing improvement in both sexual function and TCM pattern alignment.

Methods: This study is a randomized, double-blind, placebo-controlled multicentre trial. Recruitment will be conducted from patients who have a strong willingness to try using only traditional Chinese medicine treatment (This is very common in traditional Chinese medicine hospitals.). A total of 100 patients diagnosed with mild to moderate ED caused by qi deficiency and blood stasis will be recruited and randomly assigned to receive one of two treatments: HJIG (N = 50) or placebo (N = 50). Patients will receive 8 weeks of treatment and a 16-week follow-up starting from the fourth week of treatment. Outcome measures, including the International Index of Erectile Function-Erectile Function domain (IIEF-EF) score, Sexual Encounter Profile (SEP), and Traditional Chinese Medicine symptom score, will be evaluated.

Discussion: The expected outcome of this trial is that the use of the herbal formula HIJG alone can improve overall sexual satisfaction (OS) in patients with mild to moderate ED, while also improving their traditional Chinese medicine symptom scores. This will provide evidence-based support for the use of Chinese medicine in the treatment of ED in China.

Trial Registration: Chinese Clinical Trial Registry, ChiCTR2000041127, Registered on 19 December 2020, https://www.chictr.org.cn/showproj.html?proj=46469.

Trial Status: Recruitment began in March 2021, therefore 80 patients have been recruited. It is expected to finish recruiting in December 2023.

Keywords: erectile dysfunction, traditional Chinese medicine, Chinese herbal formula, Hongjing I granules, clinical trials, clinical protocols, alternative medicine

Introduction

ED is one of the most common sexual dysfunctions in men and is defined by the persistent inability of the penis to achieve or maintain sufficient erection to complete a satisfactory sexual life.[1] It is a chronic disease that seriously affects the physical and mental health of patients. Approximately 150 million men worldwide are afflicted by ED, with research projections suggesting that the global population of ED patients could reach 322 million by the year 2025.[2] Reportedly,

Received: 30 August 2023
Accepted: 10 November 2023
Published: 24 November 2023

© 2023 Xu et al. This work is published and licensed by Dove Medical Press Limited. The full terms of this license are available at https://www.dovepress.com/terms.php and incorporate the Creative Commons Attribution – Non Commercial (unported, v3.0) License (http://creativecommons.org/licenses/by-nc/3.0/). By accessing the work you hereby accept the Terms. Non-commercial uses of the work are permitted without any further permission from Dove Medical Press Limited, provided the work is properly attributed. For permission for commercial use of this work, please see paragraphs 4.2 and 5 of our Terms (https://www.dovepress.com/terms.php).

the prevalence of ED in middle-aged and elderly men can reach 52% or even higher.[3] With the aging population, the prevalence of ED in China is experiencing a rapid increase. An epidemiological meta-analysis has revealed that the overall prevalence of ED in mainland China is as high as 49.69%.[4] The prevalence is even higher among patients with underlying conditions or discomfort symptoms such as gastrointestinal diseases and diabetes.[5–7] Based on existing global reporting data, the prevalence of ED increases with age, but the affected population is becoming younger.[8]

Currently, the treatment of ED mainly relies on phosphodiesterase type 5 inhibitors (PDE5Is), with an overall efficacy rate ranging from 77% to 84%.[9] However, the effectiveness of PDE5 inhibitors for ED patients with accompanying diseases such as diabetes, severe cardiovascular disease, hyperlipidaemia, and testosterone deficiency is not ideal. In particular, for ED patients with nerve damage, the effective rate is only 35%.[10] Furthermore, only 28% of patients with severe ED can complete normal sexual activity after PDE5 inhibitor treatment.[11] Moreover, phosphodiesterase type 5 inhibitors (PDE5Is) bear costs that extend beyond the financial,[12] encompassing a spectrum of adverse effects. Therefore, it is urgent to explore safer and more effective treatment methods. Traditional Chinese medicine (TCM) has a long history of treating ED,[13] among the treatments, the most widely used are herbal compound formulas and acupuncture therapy.[14] In China, TCM formulas are increasingly being used as complementary and alternative medicine (CAM) treatments for ED patients, and their efficacy is being confirmed by an increasing number of studies.[15]

According to the "General Treatise on the Cause and Symptoms of Diseases", it is proposed that ED occurs when there is insufficient blood in the penis, making it difficult to fill the tissues. Various pathogenic factors can contribute to Qi deficiency and blood stasis as the underlying pathogenesis of impotence.[16,17] HJIG is a traditional Chinese medicine formula that possesses the therapeutic properties of invigorating Qi and promoting blood circulation. It was developed under the guidance of Professor Li Yueqing, and has been found to improve erectile function in patients with ED, preliminary studies have indicated that the combined application of tadalafil and HJIG can enhance therapeutic efficacy.[18,19] Animal experiments have shown that HJIG can promote nerve regeneration,[20] improve hypoxia, inhibit corporal cavernosum smooth muscle cells phenotype transformation,[21] and reduce tissue fibrosis.[22] Clinical studies have shown that the combination of tadalafil and HJIG is more effective in treating ED than using tadalafil alone.[23] However, many previous studies have been limited in terms of study design, sample size, and evidence. Additionally, traditional Chinese medicine (TCM) emphasizes the importance of matching herbal formulations with the specific TCM pattern types of patients.[24] Therefore, in the present protocol we will conduct a multicenter, randomized, double-blind, placebo-controlled trial to evaluate the efficacy of HJIG as a standalone treatment and its impact on the improvement of TCM pattern scores. The aim of this study is to evaluate the effectiveness and safety of traditional Chinese medicine for supplementing qi and promoting blood circulation in the treatment of ED, as well as the effectiveness of the treatment in improving the syndrome of qi deficiency and blood stasis.

Methods

Study Objectives

The objective of this study is to evaluate the safety and efficacy of the Chinese herbal formula HJIG in treating mild to moderate ED patients diagnosed with qi deficiency and blood stasis syndrome.

Design and Setting

This study is a multicenter, randomized, double-blind, placebo-controlled clinical trial conducted across five clinical sites in China. Recruitment will be conducted from patients who specifically request exclusive traditional Chinese medicine treatment from five hospitals. A total of 100 patients with ED will be randomly assigned to two groups in a 1:1 ratio, the HJIG Chinese herbal treatment group (N = 50) and the placebo treatment group (N = 50) (Flow chart. Figure 1). The SPIRIT schedule of tests and procedures for this study can be found in Figure 2. The study protocol obeyed the Standard Protocol Items for Randomized Trials statement,[25] the Declaration of Helsinki,[26] and the Good Clinical Practice guidelines. The study has been registered in the Chinese Clinical Trial Registry (ChiCTR) and the registration number was ChiCTR2000041127.

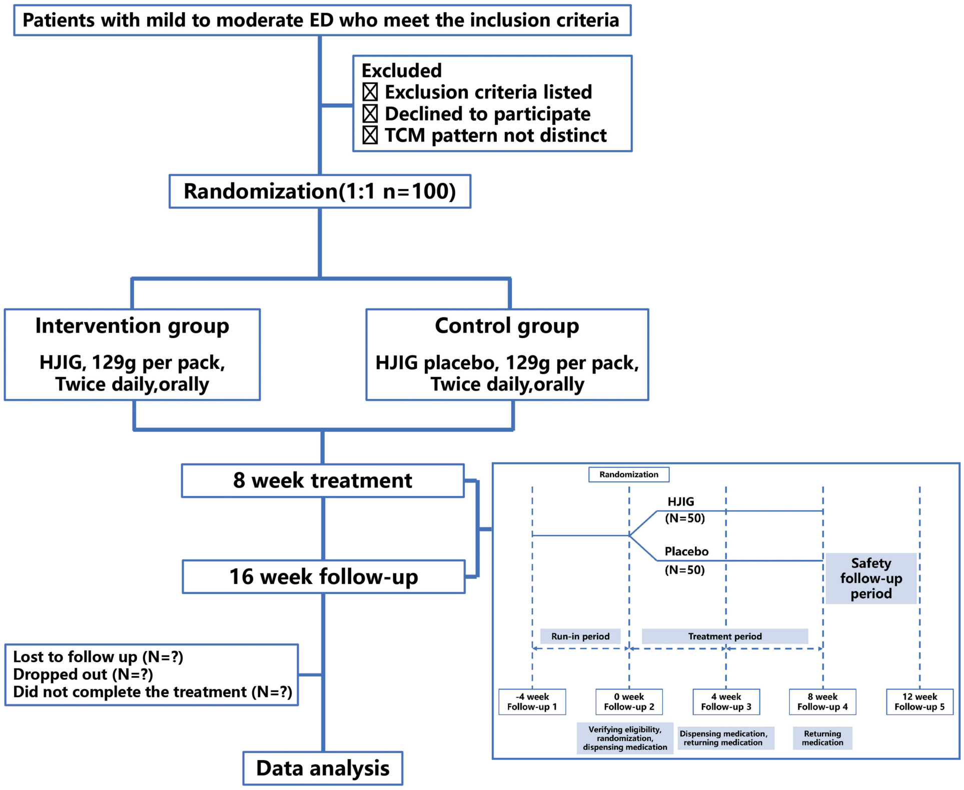

Figure 1 Flow diagram.
Abbreviations: ED, erectile dysfunction; HJIG, Hongjing I granules.

The study plan to conducted in five medical institutions in China. The study is led by the Second Affiliated Hospital of Zhejiang Chinese Medical University, with the participation of the First Affiliated Hospital of Hunan University of Chinese Medicine, the First Affiliated Hospital of Tianjin University of Traditional Chinese Medicine, Yunnan Provincial Hospital of Traditional Chinese Medicine, and Xiyuan Hospital of China Academy of Chinese Medical Sciences.

Primary Outcome Measures

The primary outcomes are the change in the percentage of intercourse events with erections lasting until ejaculation (SEP3, Sexual Encounter Profile Question 3), and changes in IIEF-EF specific scores. The secondary outcomes are the TCM symptom score and safety measures.

Inclusion Criteria

(1) According to the diagnostic criteria for impotence in traditional Chinese medicine: impotence refers to a condition in which an adult male is unable to engage in normal sexual intercourse due to a flaccid penis that either fails to achieve an erection, achieves an erection that is not firm, or achieves an erection that does not last, with the condition persisting for more than 3 months. (refer to the standard T/CACM 2015-BZ076 of the China Association of Chinese Medicine)

(2) Patients meet the diagnostic criteria for qi deficiency and blood stasis in traditional Chinese medicine

Qi deficiency (referring to the National Technical Supervision Bureau's publication of <Clinical Terminology of Traditional Chinese Medicine>, National Standard GB/16751.2-1997)

The main symptoms are shortness of breath, fatigue, weakness, lack of energy, and weak pulse.

The secondary symptoms are spontaneous sweating, reluctance to speak, and pale tongue

Study period					
	Screening period (-4 ~ 0 weeks)		Treatment period (0 ~ 8 weeks)		Follow-up period
	Visit 1	Visit 2 (confirmation of enrolment)	Visit 3	Visit 4 (Trial ended)	Telephone follow-up
	-4 weeks ±3 days	0 weeks	4 weeks ±3 days	8 weeks ±3 days	12 weeks ±3 days
Basic information					
Informed consent	X				
Registration of basic information	X				
Demographic data[1]	X				
Clinical interviewing [2]	X				
Randomization		X			
Dispensing and retrieval of investigational drugs					
Dispensing medication		X	X		
Retrieval of medication			X	X	
Efficacy assessment					
Dispensing subject diaries	X	X	X		
Retrieval of subject diaries		X	X	X	
IIEF-5	X				
TCM symptom scoring	X	X	X	X	
IIEF-EF		X	X	X	
Safety evaluation					
Physical examination[3]	X			X	
Vital signs [4]	X	X	X	X	
CBC , BBA , URE[5]	X		X	X	
Adverse events		X	X	X	X
Other records					
Polypharmacy		X	X	X	

Figure 2 SPIRIT schedule of tests and procedures 1. Demographic Data: Date of birth, height, weight. 2. Medical History: History of erectile dysfunction (ED), history of diabetes, history of cardiovascular diseases, history of other chronic diseases; surgical history, allergy history, medication history within the past 3 months; sexual history; participation in clinical trials within the past 3 months. 3. Physical Examination: Secondary sexual characteristics (skin, body shape, skeletal muscle development, Adam's apple, beard, distribution and density of body hair, male breast development); reproductive system (penis size, presence of deformities or nodules, testicles); local sensory examination (sensation in the perineal area, cremasteric reflex). 4.Vital Signs: Respiration, body temperature, pulse, blood pressure. 5.Blood Routine: White blood cell count, percentage of neutrophils, percentage of lymphocytes, red blood cell count, hemoglobin, platelet count. Biochemical Analysis: Aspartate aminotransferase (AST), alanine aminotransferase (ALT), total bilirubin, direct bilirubin, indirect bilirubin, alkaline phosphatase, blood urea nitrogen (BUN), uric acid, blood glucose, creatinine. Urine Routine: Acidity, glucose, red blood cell count (microscopic examination), white blood cell count (microscopic examination), urine protein, ketones, urinary urobilinogen.

The diagnosis of qi deficiency can be made if there are two main symptoms and one secondary symptom present.

According to the National Technical Supervision Bureau's publication of <Clinical Terminology of Traditional Chinese Medicine>, National Standard GB/16751.2-1997, blood stasis refers to the condition where blood circulation is stagnant or obstructed, leading to symptoms such as fixed pain, stabbing pain, resistance to palpation, petechiae, ecchymosis, and a purplish tongue with ecchymosis or petechiae. Other symptoms may include subcutaneous ecchymosis; blood separation; and a rough pulse that is hesitant, weak or absent.

The secondary symptoms are skin and nail disorders, numbness or paralysis of limbs, dementia, mania, forgetfulness, abnormal local sensation, and a history of trauma or surgery.

The diagnosis of blood stasis can be made if there are two main symptoms present or if one main symptom and two secondary symptoms are present.

The diagnosis of qi deficiency and blood stasis in traditional Chinese medicine includes the diagnosis of qi deficiency and blood stasis as described above.

(3) The patient is over 22 years and under 65 years old.

(4) Mild to moderate ED is present, with an IIEF-5 score of >7 and ≤21.[27]

(5) The patient has had regular heterosexual relationships for at least 3 months.

(6) The patient agrees to make at least 4 attempts at sexual intercourse every 4 weeks during the trial period.

(7) At least 4 attempts at sexual intercourse were made during the introduction period (From Visit 1 to Visit 2, 4 weeks), and the IIEF-EF special score for visit 2 was 11–25 (17–25 points for mild, 11–16 points for moderate, and ≤ 10 points for severe).

(8) The patient volunteered to participate in the experiment and gave informed consent.

Exclusion Criteria

(1) Poorly controlled diabetes (fasting blood glucose > 120% of the upper limit of the normal range).

(2) ED caused by spinal/neural injury or radical prostatectomy for prostate cancer.

(3) A history of persistent penile erection or abnormal anatomical structure of the penis (such as angular malformation, cavernous fibrosis, or penile sclerosis), or conditions making the patient prone to abnormal penile erection (such as sickle cell anaemia, multiple myeloma, or leukaemia).

(4) Use of penile implants.

(5) Severe psychological abnormalities and poor impulse control.

(6) A combination of untreated endocrine disorders such as hypogonadism, hypothyroidism, and hypopituitarism.

(7) Active testosterone replacement therapy for hypogonadism, with a stable treatment duration of less than 3 months.

(8) A history of myocardial infarction, stroke, life-threatening arrhythmia, or potential cardiovascular risk during sexual activity within the 6 months prior to enrolment, including but not limited to significant abnormalities in electrocardiogram, history of coronary artery reconstruction surgery, unstable angina, sexual angina, congestive heart failure, significant cardiomyopathy, or moderate or severe cardiovascular disease, as determined by the researcher.

(9) Aspartate aminotransferase (AST) and alanine aminotransferase (ALT) levels more than two times the upper limit of normal; a creatinine level more than two times the upper limit of normal.

(10) Bleeding disorders or active gastrointestinal ulcers.

(11) Hypotension at rest (blood pressure below 90/50 mmHg) or poorly controlled hypertension (blood pressure above 170/100 mmHg).

(12) A history of alcohol abuse (drinking more than 14 alcohol units per week; one bottle of 350 mL beer, 120 mL wine, or 30 mL of 40% alcohol spirit is equivalent to 1 alcohol unit) or drug abuse in the past 6 months.

(13) Inability to cooperate in order to complete the required study records.

(14) Obvious comorbid diseases.

Withdrawal Criteria

The subjects can withdraw from the study at any time for any reason, including but not limited to the following:

(1) Withdrawal of informed consent.

(2) Loss to follow-up. In this case, the researcher is required to make at least 3 phone calls to notify the subject to follow up and record, but the subject still refuses to continue participating in the trial.

(3) Dissatisfaction with drug efficacy.

(4) Dissatisfaction with drug safety.

Alternatively, the researcher may decide to withdraw the subject from the study for the following legitimate reasons:

(1) The subject experiences intolerable adverse events.

(2) The subject is unable to comply with the requirements of the study protocol for medication use, testing, and provision of relevant research data.

(3) The subject is unblinded during the trial.

(4) The sexual partner becomes pregnant during the experiment.

Removal Criteria

Before conducting statistical analysis, the statistician, sponsor, and primary investigator of the clinical research team will comprehensively determine whether to exclude the subject based on factors such as the completion of the study by the subject and reasons for withdrawal and make relevant explanations in the statistical report.

(1) Subjects who do not meet the inclusion criteria or meet the exclusion criteria and who cannot be exempted as determined jointly by the investigator and sponsor.

(2) Subjects who have not used the investigational drug.

(3) Subjects who have no study data.

Study Termination Criteria

The sponsor may terminate the clinical study at any time if any of the following conditions occur:

(1) Serious safety issues are discovered during the study.
(2) The efficacy of the investigational drug is found to be lower than expected during the study and has no development value.
(3) Significant deviation in the conduct of the trial is found during the study, resulting in the inability to obtain the expected study data.
(4) The National Medical Products Administration or the Ethics Committee orders the study to be terminated.
(5) The sponsor initiates termination.

When the study is terminated, the sponsor shall immediately notify all principal investigators of clinical research units, ethics committees, and relevant regulatory authorities and submit relevant written explanations.

Randomization and Blinding

The present study mainly adopts stratified block randomization for grouping. First, the centres are stratified, with 20 subjects in each centre, divided into a control group and an experimental group. Then, 20 subjects in each centre are randomly assigned to subgroups again based on the time of visit as the matching factor. Four patients with adjacent visit times were considered one subgroup. The specific operation method is as follows: (1) Determine the length of the subgroup and all possible arrangements of the two groups; (2) Assign a sample number to each possible arrangement of the subgroup; and (3) Randomly arrange the subgroup assignment number by drawing lots.

Drug Preparation

This study involved two drugs, namely, HJIG and HJIG placebo. The appearance, odour, and taste of HJIG and HJIG placebo are completely identical. They are placed in identical packaging bags and boxes, with the packaging bag sealed and the opening of the packaging box affixed with a seal strip. Only the subjects can open it when taking the medication. Labels are affixed to the outside of the packaging box and the body of the packaging bag, indicating the trial information but not the type of drug in the bottle.

Blinding Envelopes

The statistician prepares two levels of blinding envelopes, with two copies of the same envelope. After the statistician completes the blinding of the drugs, the blinding envelope is sealed, and the name of the blinding personnel and the time of blinding were signed at the seam of the envelope. The sponsor and the primary investigator of the clinical research team each keep a copy, and the blinding envelope should be properly preserved until the end of the clinical study.

Unblinding

This study stipulates two unblinding times. The first unblinding time is after the blind check is completed and the database is locked, and the second unblinding time is after the statistical analysis report is completed. Each unblinding process must be jointly conducted by the statistician, sponsor, and primary investigator of the clinical research team, and the name of the unblinding personnel and the time of unblinding must be signed on the blinding envelope.

Unblinding can be requested by the statistician only when an adverse event (AE) occurs in the subject and the investigator determines that the handling of the AE requires knowledge of which investigational drug the subject is using. The applicant can view the unblinding results provided by the statistician.

Placebo Control

As psychological factors can also cause ED and the effectiveness of ED treatment is mainly based on the subjective feelings of the subjects, this trial uses a placebo control.

Drug and Intervention

Investigational drug: Hongjing I granules (produced by Jiangyin Tianjiang Pharmaceutical Co., Ltd.) (a basic formula for promoting qi and activating blood circulation, a prescription for the treatment of male impotence in the Department of Urology, Second Affiliated Hospital of Zhejiang Chinese Medical University, composed of Hongjingtian, Huangqi, Dangshen, Danshen, goji berry, *Epimedium, Angelica*, White Peony, and Szechuan Lovage Rhizome); see Table 1 for specific ingredients.

Placebo

The corresponding placebo of the Chinese herbal granules used in the treatment group (produced by Jiangyin Tianjiang Pharmaceutical Co., Ltd.) (similar in appearance, colour, and odour to HJIG Figure 3).

Table 1 Composition of HJIG

Chinese Herbal Medicine Name	Latin Name	Dosage of Herbal Medicine	Dosage of Granules in the Prescription
Hong-Jing-Tian	Rhodiola rosea.	15 g	1.5 g
Zhi-Huang-Qi	Radix Astragali.	20 g	6 g
Chao-Dang-shen	Codonopsis pilosula.	15 g	4.5 g
Quan-Dang-Gui	Angelica sinensis.	12 g	4.8 g
Dan-shen	Salvia miltiorrhiza.	15 g	3 g
Bai-Shao	Radix Paeoniae Alba.	15 g	1.5 g
Gou-Qi	Lycium chinense Miller.	15 g	6 g
Yin-Yang-Huo	Epimedium brevicornum.	10 g	0.5 g
Chuan-Niu-Xi	Cyathula officinalis.	12 g	3.6 g

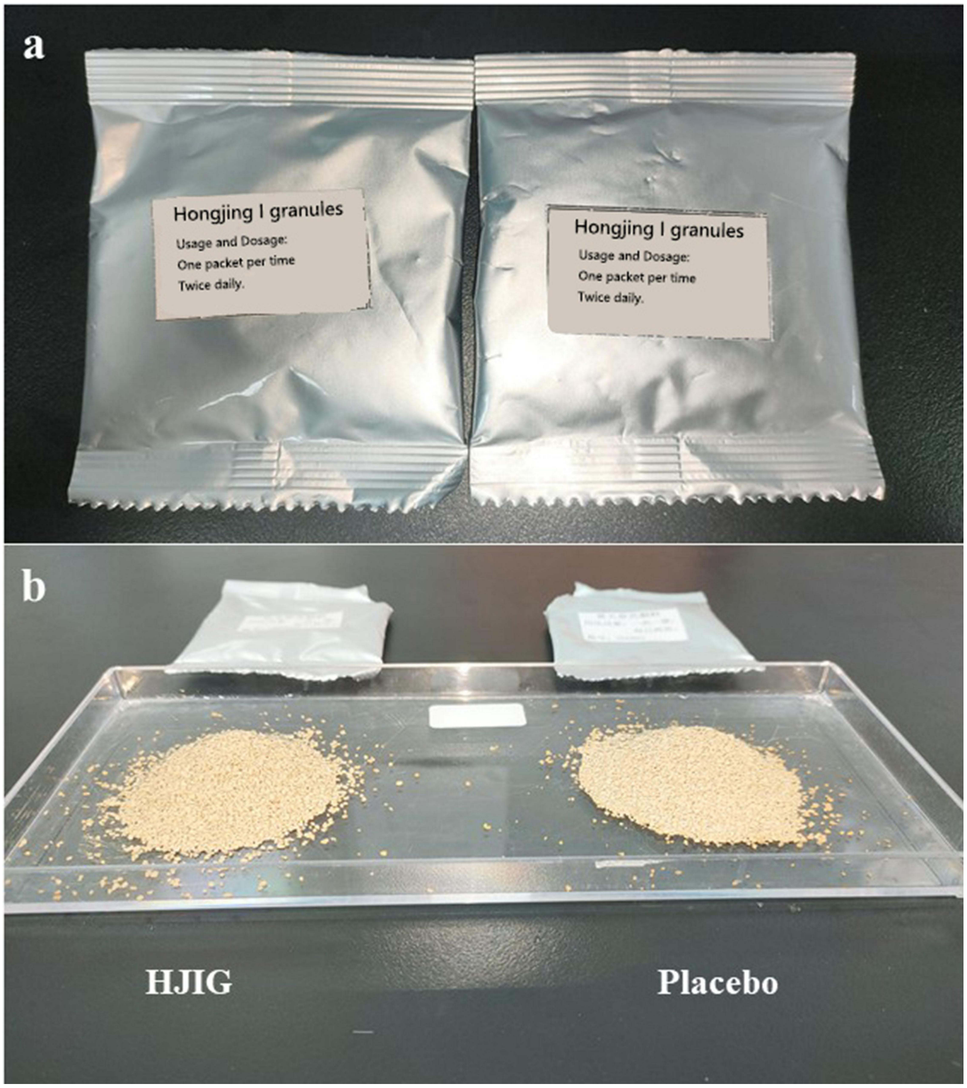

Figure 3 Packaging and appearance of Hongjing I granules (HJIG) and its placebo (**a**) HJIG and its placebo packaging (**b**) The appearance of HJIG granules and placebo granules (Left side is HJIG and right side is placebo granules).

Dispensing and Recovery of Drugs

After subject enrollment, drug numbers were allocated based on randomization. The drug administration was conducted by authorized personnel who were responsible for dispensing and recovering drugs. During dispensation, the integrity of the seal strip was verified, and subjects were reminded to check if the packaging had been previously opened. The quantity of drugs dispensed was recorded, and recipients signed the receipt form. During drug return, the correct drug numbers were confirmed, and the quantity was counted. Usage and lost drugs were documented. Drug dispensation occurred during the second visit, while drug recovery took place during the third visit. At the end of the trial, the drugs were returned to the sponsor.

Administration Method

The investigational drug in this trial is dispensed by the research nurse or designated personnel designated by the investigator, confirmed for relevant information, and then dispensed to the subjects. The subjects used granules for treatment. The granules (produced by Jiangyin Tianjiang Pharmaceutical Co., Ltd.) are dissolved in 200 mL of hot water and taken twice a day, in the morning and afternoon.

Recording Drug Administration Time

The subjects must record the time and quantity of drug administration on the subject diary cards.

Medication Cycle

Each experimental cycle lasts 4 weeks. During each cycle, participants are required to conduct at least 4 sexual intercourse attempts and take medication according to the specifications. Medications that have not been taken completely during the cycle must be kept in the original packaging bag and box (the original packaging bag and box must be kept after taking the medication). After 4 weeks, the subjects returned to the clinical unit for follow-up and returned the remaining drugs.

Medication Adherence

Medication adherence (%) = (actual number of doses taken/theoretical number of doses prescribed) × 100%. The theoretical number of doses prescribed is the total number of doses prescribed to the subject twice daily for a period of four weeks. The actual number of doses taken is the number of doses prescribed minus the number of doses returned. If the medication adherence is less than 80%, the subject is considered nonadherent to the medication, and the reasons should be investigated and recorded.

Concomitant Medication

The following rules for concomitant medication during the trial must be followed, and the investigator must provide corresponding guidance to the subjects regarding concomitant medication and daily life during the trial. All concomitant medications must be recorded in the concomitant medication record form. 1. It is strictly prohibited to use other drugs or treatments for ED during the trial; 2. It is strictly prohibited to use other Chinese herbal medicines or Chinese patent medicines concomitantly.

Outcome Indicators

Primary Outcome

Assessing the effectiveness of Hongjing I granules in treating ED through the evaluation of two composite endpoints.

The composite endpoints are changes in the following two questions at Visit 4 compared to Visit 2:

(1) The change in the percentage of erections that can last until the completion of ejaculation as a percentage of total sexual intercourse (Sexual Diary SEP3).

(2) Changes in the IIEF-EF Special Score.

Evaluate the difference between the experimental group and the placebo group separately. It can be considered that the primary endpoint is achieved only if both endpoint indicators of the experimental group are better than those of the placebo group.

Secondary Indicators

(1) Stratified subgroup analysis of IIEF-EF and SEP results at Visit 4 based on participants' baseline characteristics, such as age (>32 years old, ≤32 years old) and duration of ED (<12 months, ≥12 months but <36 months, and ≥36 months).

(2) The percentage change in the number of times an erection can be inserted into the vagina at visits 3 and 4 compared to visit 2 (SEP2).

(3) The percentage change in SEP3 and the IIEF-EF score at visit 3 compared to visit 2.

(4) Changes in traditional Chinese medicine symptom scores (Supplementary File) at visits 3 and 4 compared to visit 2.

(5) Alteration in scores reflecting sexual desire (frequency of sexual activities) and overall satisfaction with sexual life between Visit 3 or Visit 4 and Visit 2.

(6) The percentage of subjects with restored normal IIEF-EF scores during visit 4 compared to visit 2.

Safety Outcomes

The safety outcomes and vital signs in patients will be tested at baseline and every month during medication, and detailed results will be recorded on the CRF in a timely fashion. In addition, all AEs occurring throughout the study will be closely observed and documented by the investigator, and the patients will be appropriately managed.

Adverse Event

All adverse events that occur during the trial process will be assessed.

Data Management

In this study, we will record patients' data on paper case report forms (CRFs). The clinical investigator or clinical coordinator nominated by the investigator will input the data from the study medical record to CRFs promptly and accurately. The study supervisor will make site visits to review protocol compliance, compare CRFs against individual patients' medical records, and verify whether the drugs that are supplied, received, stored, distributed, and recovered, are recorded accordingly in accordance with relevant regulations. CRFs will be kept in a locked file cabinet that is pertinent to this research. Data management will be performed by the investigator and monitored by an independent supervisor. Information regarding Contraceptive Guidelines and Safety Observation Indicators, as well as the assessment forms (Erectile Function, Sexual Function, and Qi Deficiency/Blood Stasis Scores) that are to be recorded in the Case Report Form (CRF), can be found in supplementary File. Examples of the Informed Consent Form (for both partners) are also available in supplementary File.

Sample Size

The subjects in this study were divided into two groups, namely, HJIG group and placebo group. The hypothesis to be validated is: the superiority of the HJIG group compared to the placebo group. The superiority indices to be validated include: ① SEP3; ② The change in the IIEF-EF specific score. All superiorities are statistical superiorities, and no superiority threshold is set. According to related references, the test level is 0.05, and the test assurance is set to 0.9. The formula for the superiority test with a 1:1 ratio allocation between the experimental group and the control group is:

$$\mathrm{n} = \frac{(Z_\alpha + Z_\beta)^2[P_C(1-P_C)+(1-P_T)]}{(P_T-P_C)^2}$$

In this formula, P_C is the control group rate, P_T is the test group rate. α is the first type of error, usually taken as α=0.05, then Z_α=1.645; β is the second type of error, usually taken as β=0.10, then Z_β= 1.282. According to publicly published research data, the clinical effectiveness rate of placebo in ED clinical research is PC=0.33.[28] Our preliminary pre-experiment statistics show that the clinical effectiveness rate of HJIG is PT=0.65. Take α=0.05, β=0.10, substitute the relevant data into the superiority test formula, get n≈40, considering a 20% drop-out rate, it is expected that 50 cases will be required for each group, with a total of 100 cases included in the group.

Statistical Analysis

For continuous variables, if the data do not follow a normal distribution, they will be described using the median [interquartile range] format; if they follow a normal distribution, the mean (standard deviation) format will be used. For categorical variables, the data will be described as the number of cases (percentage). For categorical variables, the data will be described as the number of cases (percentage). Before comparing the differences between the experimental group and the control group for various indicators, we will use the Shapiro–Wilk test to check the normality of continuous variables. If the variable passes the normality test, the *t* test will be used; otherwise, the Wilcoxon test will be employed. Additionally, for the comparison of categorical variables, the chi-square test will be used. In this study, we will use the baseline disease status as a reference to compare the changes in IIEF-EF domain scores, frequency of sexual encounters, and number of erections that persisted until ejaculation was completed as a proportion of the total number of sexual intercourse sessions (SEP3) after treatment (4 weeks vs 0 weeks, 8 weeks vs 0 weeks, and 12 weeks vs 0 weeks). Similarly, for secondary outcomes, we will compare the changes in SEP1, SEP2, SEP4, SEP5, and the scores of different

traditional Chinese medicine syndromes before and after treatment. Considering the significant impact of age and ED history on treatment outcomes, we will use the median values of age (32 years) and ED history (12 units) as cut-off points and to stratify the study population by age (≤32 or >32 years) and ED history (≤12, >12) for subgroup analysis. (Analyses will be conducted using R software (version 4.2.3). A P value of <0.05 will be considered statistically significant.)

Discussion

Traditional Chinese medicine emphasizes individualized treatment based on syndrome differentiation.[29] The selection of Chinese herbal medicine formulas that match the characteristic symptoms of traditional Chinese medicine syndromes can alleviate or improve diseases. Traditional Chinese medicine and herbal medicine have a long history and rich experience in the treatment of ED.[30] Traditional Chinese medicine believes that normal erection requires the coordination of human organs, meridians, qi and blood and the cooperation of the five organs, as well as unobstructed meridians and abundant qi and blood, resulting in a strong and lasting erection. Multiple early-stage clinical studies have been conducted to confirm that the combination of HJIG and PDE5i can improve the efficacy of treating ED.[19,23]

However, there is still a lack of high-quality evidence for the clinical efficacy of the Chinese herbal compound HJIG as monotherapy. Therefore, we will conduct a multicentre, randomized, double-blind, placebo-controlled trial to evaluate the safety and effectiveness of the Chinese herbal formula HJIG in patients diagnosed with qi deficiency and blood stasis–type ED. This study may establish a new treatment method for ED patients, distinguishing them from other drugs used for similar clinical indications in clinical practice. The International Index of Erectile Function – Erectile Function (IIEF-EF) score is an internationally recognized method for evaluating erectile function and sexual satisfaction in patients.[31,32] Because the improvement of OS is crucial for patients with ED, in our study, the IIEF-EF subscale score and the Sexual Encounter Profile (SEP3) were chosen as primary outcomes. We will also assess the traditional Chinese medicine (TCM) score for qi deficiency and blood stasis syndrome in patients. These measurements will provide us with evidence of whether herbal medicine for tonifying qi and promoting blood circulation can improve EF in patients with TCM syndrome differentiation.

This study has strict controls for blinding, drug administration, and placebo to ensure the reliability of the research data. We have provided a detailed description of statistical analysis methods in the article. In addition, the outcome evaluation will be conducted by independent clinical statisticians who are unaware of group allocation and not involved in providing interventions or management. Due to the strict control of TCM syndrome differentiation to ensure targeted treatment and the strict inclusion criteria may lead to recruitment difficulties, we have adopted the minimum sample size. Although the sample size of this study is relatively small compared to the number of patients with ED, the "National Key Specialty Cooperation Diagnosis and Treatment Research Platform" (China) developed by our team has included 671 patients with ED. Analysis shows that although the qi deficiency and blood stasis type of patients only account for approximately 10% of all ED patients, a high percentage of 58.9% showed complete inability to erect. In addition, among 21 patients with neurogenic ED, 18 (85.7%) showed complete inability to erect, and 9 (43%) were diagnosed with qi deficiency and blood stasis, which is much higher than the proportion of 8.3% in all impotence patients, indicating that qi deficiency and blood stasis may be a basic symptom of difficult-to-treat ED, such as neurogenic ED. Therefore, we strictly controlled the "qi deficiency and blood stasis" syndrome score as an inclusion criterion.

As this study is the first double-blind, placebo-controlled, multicentre randomized controlled trial of traditional Chinese medicine for the treatment of ED, we chose only two internationally recognized scales to evaluate the subjective sexual experience of ED patients. Objective detection methods such as nocturnal penile tumescence and rigidity (NPTR) will be considered in future studies. The strengths of this trial include strict methods and quality control measures. We have detailed our recruitment methods, blinding procedures, data collection and statistical methods, and medication handling methods in accordance with the *Recommendations for Reporting Randomized Controlled Trials of Herbal Interventions*.[33] In addition, the development of this protocol was guided by the Consolidated Standards of Reporting Trials 2017 (CONSORT 2017)[34] statement, the recommendations for intervention trials (SPIRIT) and guidance for protocols of clinical trials.[25] The results of this study are expected to provide further evidence for the safety and efficacy

of the Chinese herbal formula HJIG in the treatment of mild to moderate ED and to observe whether there is a simultaneous improvement in erectile function and traditional Chinese medicine syndromes in ED patients.

Ethics Approval and Consent to Participate

This study conformed to the ethics principles set forth by the Declaration of Helsinki. All patients were required to sign an informed consent form before being enrolled in the study. This study was approved by the Ethics Committee of the Second Affiliated Hospital, Zhejiang Chinese Medical University, with the identifier 2019-KL-012-01(S1 proof of ethics approval). Any revisions of the study protocol will be submitted to the ethics committee.

Acknowledgments

We would like to thank Dr Chunbao Mo (School of Medicine, Southern University of Science and Technology, Shenzhen, Guangdong, China) for providing professional statistical advice during the design of this study and for providing support in the subsequent data analysis.

We would like to thank Professor Ferguson D for creating the Sexual Encounter Profile (SEP) questionnaire, which has been pivotal for sexual dysfunction research. We have been unable to find the original literature or copyright information for SEP and only have your name from the eprovide website. If there are any requirements for the use of SEP, including any fees or permissions, please contact us.

The findings will be presented at relevant research conferences, seminars, and academic meetings. They will also be published in a peer-reviewed international journal from the cognitive sciences sector.

Funding

Zhejiang Province Traditional Chinese Medicine Prevention and Control Major Disease Research Plan (No.2018ZY007); National Natural Science Foundation of China (No.82174376); The Zhejiang Provincial Administration of Traditional Chinese Medicine solely conducts peer review of the project process and provides project funding after approval (S1 proof of external funding). Foundation provides research funds and evaluates the research results, but is not involved study design, data collection, analysis, the decision to publish or preparation of the manuscript.

Disclosure

The authors report no conflicts of interest in this work.

References

1. Hatzimouratidis K, Amar E, Eardley I, et al. Guidelines on male sexual dysfunction: erectile dysfunction and premature ejaculation. *Eur Urol*. 2010;57(5):804–814. doi:10.1016/j.eururo.2010.02.020
2. Costa P, Potempa KJ. Intraurethral alprostadil for erectile dysfunction: a review of the literature. *Drugs*. 2012;72(17):2243–2254. doi:10.2165/11641380-000000000-00000
3. Johannes CB, Araujo AB, Feldman HA, et al. Incidence of erectile dysfunction in men 40 to 69 years old: longitudinal results from the Massachusetts male aging study. *J Urol*. 2000;163(2):460–463. doi:10.1016/S0022-5347(05)67900-1
4. Wang W, Fan J, Huang G, et al. Meta-analysis of prevalence of erectile dysfunction in mainland china: evidence based on epidemiological surveys. *Sex Med*. 2017;5(1):e19–e30. doi:10.1016/j.esxm.2016.10.001
5. Romano L, Pellegrino R, Sciorio C, et al. Erectile and sexual dysfunction in male and female patients with celiac disease: a cross-sectional observational study. *Andrology*. 2022;10(5):910–918. doi:10.1111/andr.13186
6. Romano L, Zagari RM, Arcaniolo D, et al. Sexual dysfunction in gastroenterological patients: do gastroenterologists care enough? A nationwide survey from the Italian Society of Gastroenterology (SIGE). *Digest Liver Dise*. 2022;54(11):1494–1501. doi:10.1016/j.dld.2022.05.016
7. Malavige LS, Levy JC. Erectile dysfunction in diabetes mellitus. *J Sex Med*. 2009;6(5):1232–1247. doi:10.1111/j.1743-6109.2008.01168.x
8. Nguyen HMT, Gabrielson AT, Hellstrom WJ. Erectile dysfunction in young men—a review of the prevalence and risk factors. *Sexl Med Rev*. 2017;5(4):508–520.
9. Kendirci M, Tanriverdi O, Trost L, et al. Management of sildenafil treatment failures. *Curr Opin Urol*. 2006;16(6):449–459. doi:10.1097/01.mou.0000250286.60237.a6
10. Matthew AG, Goldman A, Trachtenberg J, et al. Sexual dysfunction after radical prostatectomy: prevalence, treatments, restricted use of treatments and distress. *J Urol*. 2005;174(6):2105–2110. doi:10.1097/01.ju.0000181206.16447.e2
11. Dubbelman YD, Dohle GR, Schroder FH. Sexual function before and after radical retropubic prostatectomy: a systematic review of prognostic indicators for a successful outcome. *Europ urol*. 2006;50(4):711–720.
12. Travison TG, Hall SA, Fisher WA, et al. Correlates of PDE5i use among subjects with erectile dysfunction in two population-based surveys. *J Sex Med*. 2011;8(11):3051–3057.

13. Ma WG, Jia JM. The effects and prospects of the integration of traditional Chinese medicine and Western medicine on andrology in China. *Asian J Androl*. 2011;13(4):592–595. doi:10.1038/aja.2010.127
14. Wang H, Yan B, Zhao M, et al. Efficacy of acupuncture in the treatment of psychogenic erectile dysfunction: study protocol for a randomized control trial. *J Men Health*. 2023;19(7):31–38.
15. Wang Y, Geng L, He C, et al. Chinese herbal medicine combined with tadalafil for erectile dysfunction: a systematic review and meta-analysis. *Andrology*. 2020;8(2):268–276.
16. Deadman P. The treatment of erectile dysfunction by acupuncture. *J Chn Med*. 2007;2007:85.
17. Xudong DY, Wang JS, Zuo G, et al. Traditional Chinese medicine on treating diabetic mellitus erectile dysfunction: protocol for a systematic review and meta-analysis. *Medicine*. 2019;98(13):1.
18. Gaoyue Z, Jianfeng Z, Junfeng Y, et al. Research of Tonifying Qi and activating blood combined with tadalafil for moderate erectile dysfunction by down stairs. *J Liaoning Uni Trad Chn Med*. 2017;2017:1.
19. Ping Z, Wenzhi W, Zhongming D, et al. The therapeutic effect of Hongjing No.1 prescription combined with Tadalafil therapy in erectile dysfunction patients by CT corpus cavernosum. *Chn J Human Sex*. 2021;30(9):4–9.
20. Ma K, Zhao F, Ye MY, et al. Neuroprotective effect of Hongjing I granules on erectile dysfunction in a rat model of bilateral cavernous nerve injury. *Biomed Pharmacother*. 2020;130:110405. doi:10.1016/j.biopha.2020.110405
21. Xiaojun H, Bodong L, Gang C, et al. Protective effects of Hongjing Yihao (HJYHF) on phenotypic modulation of corpus cavernosum smooth muscle cells. *Chin Arch Tradit Chin Med*. 2012;30(9):2027–2029.
22. Ye MY, Zhao F, Ma K, et al.. Effect of HongJing I in treating erectile function and regulating RhoA pathway in a rat model of bilateral cavernous nerve injury. *Evid Based Complement Alternat Med*. 2019:1083737. doi:10.1155/2019/1083737
23. Gaoyue Z, Jianfeng Z, Junfeng Y, et al. Discussion on the treatment of mild and moderate erectile dysfunction with combination of yiqihuoxue method and low-dose tadalafil descending stairs. *Liaoning Univ Tradit Chin Med*. 2017;19(08):77–80.
24. Zhang X, Tian R, Zhao C, et al. The use of pattern differentiation in WHO-registered traditional Chinese medicine trials–A systematic review. *Eur J Integ Med*. 2019;30:100945.
25. Chan AW, Tetzlaff JM, Altman DG, et al. SPIRIT 2013 statement: defining standard protocol items for clinical trials. *Ann Intern Med*. 2013;158 (3):200–207. doi:10.7326/0003-4819-158-3-201302050-00583
26. Association W M. World medical association declaration of helsinki: ethical principles for medical research involving human subjects. *JAMA*. 2013;310(20):2191–2194.
27. Ponholzer A, Temml C, Mock K, et al. Prevalence and risk factors for erectile dysfunction in 2869 men using a validated questionnaire. *Eur Urol*. 2005;47(1):80–85. doi:10.1016/j.eururo.2004.08.017
28. Porst H, Giuliano F, Glina S, et al. Evaluation of the efficacy and safety of once-A-day dosing of tadalafil 5 mg and 10 mg in the treatment of erectile dysfunction: results of a multicenter, randomized, double-blind, placebo-controlled trial. *Europ urol*. 2006;50(2):351–359.
29. Wang Q. Individualized medicine, health medicine, and constitutional theory in Chinese medicine. *Front Med*. 2012;6(1):1–7. doi:10.1007/s11684-012-0173-y
30. Leisegang K, Finelli R. Alternative medicine and herbal remedies in the treatment of erectile dysfunction: a systematic review. *Arab J Urol*. 2021;19(3):323–339. doi:10.1080/2090598X.2021.1926753
31. Rosen RC, Riley A, Wagner G, et al. The international index of erectile function (IIEF): a multidimensional scale for assessment of erectile dysfunction. *Urology*. 1997;49(6):822–830. doi:10.1016/s0090-4295(97)00238-0
32. Rosenx RC, Cappelleri JC, Smith MD, et al. Development and evaluation of an abridged, 5-item version of the International Index of Erectile Function (IIEF-5) as a diagnostic tool for erectile dysfunction. *Int J Impot Res*. 1999;11(6):319–326. doi:10.1038/sj.ijir.3900472
33. Cheng CW, Wu TX, Shang HC, et al. CONSORT extension for Chinese herbal medicine formulas 2017: recommendations, explanation, and elaboration. *Ann Intern Med*. 2017;167(2):112–121. doi:10.7326/M16-2977
34. Moher D, Hopewell S, Schulz KF, et al. CONSORT 2010 explanation and elaboration: updated guidelines for reporting parallel group randomised trials. *Int J Surg*. 2012;10(1):28–55. doi:10.1016/j.ijsu.2011.10.001

International Journal of General Medicine Dovepress

Publish your work in this journal

The International Journal of General Medicine is an international, peer-reviewed open-access journal that focuses on general and internal medicine, pathogenesis, epidemiology, diagnosis, monitoring and treatment protocols. The journal is characterized by the rapid reporting of reviews, original research and clinical studies across all disease areas. The manuscript management system is completely online and includes a very quick and fair peer-review system, which is all easy to use. Visit http://www.dovepress.com/testimonials.php to read real quotes from published authors.

Submit your manuscript here: https://www.dovepress.com/international-journal-of-general-medicine-journal
